# Physical activity interventions to improve physical function in temporarily non-ambulant older persons: a scoping review

**DOI:** 10.3389/fragi.2026.1816647

**Published:** 2026-04-15

**Authors:** Elma van Garderen, Mandy Visser, Wilco P. Achterberg

**Affiliations:** 1 Department of Public Health and Primary care, Leiden University Medical Center, Leiden, Netherlands; 2 Research Department, Topaz, Leiden, Netherlands

**Keywords:** inactivity, non-ambulant, non-weight bearing, older persons, physical activities, rehabilitation

## Abstract

**Introduction:**

Older persons who are temporarily non-ambulant are often confronted with the adverse health effects of physical inactivity. These adverse health effects include sarcopenia, reduced strength, reduced health-related quality of life and increased mortality. To counteract those negative effects of immobility, it is important to know how older persons can stay physically active when they are temporarily unable to ambulate. Therefore, the aim of this scoping review was to provide an overview of physical activity interventions that can be performed by older persons who are temporarily non-ambulant.

**Method:**

A literature search was performed through PubMed, EMCARE, EMBASE, CINAHL, Web of Science, Cochrane Library, PEDro, Academic search premier and Epistemonikos in August 2023 and updated in October 2024 and December 2025. Included were full-text, English-language articles, describing physical activity interventions for older persons who were temporarily non-ambulant.

**Results:**

Ten studies met the inclusion criteria. The physical activity interventions described in the studies were wheelchair mobilization, lower extremity strength training, (seated) physical activity programmes and neuromuscular electric stimulation. The effects of these interventions on physical fitness include; the ability to ambulate and walk, increase in muscle mass and power, slower decline in physical activity and a decrease in fear of falling.

**Conclusion and implications:**

Studies regarding physical activity interventions that can be performed by older persons who are temporarily non-ambulant are scarce, even though being temporarily non-ambulant is common among older persons. This review highlights the need for future studies on how we can help temporarily non-ambulant older persons to remain physically active. We recommend to conduct future studies for the development of a hospital and in-patient geriatric rehabilitation guideline for those older persons.

## Introduction

As life expectancy increases, so does the number of older persons that are admitted to hospitals. The medical conditions that lead to hospitalization (such as fractures, pneumonia, heart attack and stroke) and interventions during hospital stay, frequently causes result in temporary loss of mobility and ambulation. As a result, older patients are often confronted with the negative effects of physical inactivity (Brown et al., 2021). A period of bed rest as short as 5 days already leads to reduced leg lean mass and strength (Tanner et al., 2015). Longer periods of immobility leads to the loss of muscle mass, strength, muscle volume, muscle respiratory capacity and neuronal motor function (Breen et al., 2013; Psatha et al., 2012; Gram et al., 2014; Rejc et al., 2018; Suetta et al., 2009). Other associated complications of physical inactivity include an increased risk of pressure ulcers and pneumonia, reduced health-related quality of life and increased mortality (Wu et al., 2018). In addition, hospitalization, disease related immobility, and prolonged bedrest has been associated with the development of sarcopenia (Cruz-Jentoft et al., 2019; Wan et al., 2023; Welch et al., 2018). Sarcopenia is associated with increased risk of adverse outcomes including falls, fractures, physical disability and mortality (Cruz-Jentoft et al., 2019).

Especially in older persons prolonged bed rest impacts function. It is associated with an increased risk of loss of independence after hospital discharge, poorer function 2 months post-surgery and poorer 6-month survival compared with older persons who are able to ambulate at the start of the rehabilitation (Grill et al., 2007; Siu et al., 2006). The impact of immobility on loss of muscle mass and neuromuscular response is greater in older persons than in young persons (Rejc et al., 2018; Suetta et al., 2009). Additionally, compared to the retraining of younger persons, the response of older persons to retraining is less effective. Which indicates that older persons need a longer period with more training sessions to recover from a period of immobility (Rejc et al., 2018; Suetta et al., 2009). Thus, not only are older persons more negatively impacted during the period of immobility, they also have more difficulty recovering from immobility compared to younger persons.

To reduce the risk in getting complications from physical inactivity, many hospitals encourage early mobilization. For example, The Royal Dutch Society for Physical Therapy has developed a course and guideline called ‘movement hospitals’ to help physiotherapists promote physical activity in the hospitals where they work (KNGF Standpunt Beweegziekenhuizen, 2021). However, early ambulation is not always possible. Factors such as pain, weight-bearing restrictions, comorbidities or cognitive impairment are associated with a delay in ambulation (Dubljanin-Raspopović et al., 2013; Ekegren et al., 2021; Mariconda et al., 2016; Morri et al., 2018). It is unclear if and how older persons can maintain physical activity during periods of temporary immobility, in order to counteract its negative effects. Therefore the aim of this scoping review was to provide an overview of physical activity interventions that can be performed by older persons who are temporarily non-ambulant, and of the effects of those interventions on physical function.

## Methods

### Review methodology

This scoping review allowed for a comprehensive coverage of the available literature, including different types of physical activity interventions, outcomes, and reasons for which older persons are temporarily non-ambulant (Arksey and O'Malley, 2005; Maggio et al., 2021). By taking a broad view of the existing literature, we were also able to identify research gaps (Arksey and O'Malley, 2005; Maggio et al., 2021). We used the PCC (population, concept and context) framework for constructing the research question, 1) population: Older persons who are temporarily non-ambulant, 2) Concept: Physical activity interventions, and 3) context: Effects on physical function (Pollock et al., 2023). Arksey and O’Malley’s methodological framework was used to conduct the scoping review (Arksey and O'Malley, 2005). We followed the PRISMA Extension for Scoping Reviews reporting guideline (Tricco et al., 2018). A comprehensive description of the methodology protocol of this scoping review has been published on the Open Science Framework platform (Van Garderen, 2025).

### Inclusion and exclusion criteria

Studies were included if they met the following criteria:

*Participants:* The study included persons aged 60 years and older who were temporarily unable to ambulate (with a Functional Ambulation Category score of 0 or 1 (Universitair Netwerk Ouderenzorg and De Backer, 2018)) due to medical conditions (e.g., injuries, surgeries, acute illness), or were restricted in their ambulation due to the protocol of the study. Studies in which different age groups were examined, and the results for the older persons’ group aged 60 years and older was reported separately, were also eligible for inclusion.

Definition mobilization and ambulation: The International Classification of Functioning, Disability and Health (ICF) framework of the World Health Organization (WHO) defines mobility as “*moving by changing body position or location or by transferring from one place to another, by carrying, moving or manipulating objects, by walking, running or climbing, and by using various forms of transportation*.” (World Health, 2001). The term mobility is widely used in different contexts with different meanings, including travelling, going from one place to another place, but it can also refer to moving parts of the body while staying in one place (Metz, 2000). In this review, mobility refers to a person’s ability to change and control their body position, which requires sufficient muscle strength and energy, along with adequate skeletal stability, joint function, and neuromuscular synchronization (Ernstmeyer, 2021). Ambulation, or the ability to walk, is a form of mobilization (Ernstmeyer, 2021). In this study we look at mobilization in older persons who are temporarily non-ambulant, meaning they are temporarily wheelchair or bed-bound. We deemed studies with healthy older persons who were experimental non-ambulant as relevant, since a period of bed rest as short as 5 days already leads to reduced leg lean mass and strength in healthy older persons (Tanner et al., 2015).

*Interventions:* The study examined or observed physical activity interventions. Studies in which physical activity was reported as part of the intervention were also considered for inclusion.

*Outcomes:* The study describes outcomes of the physical activity interventions regarding physical function, which may be reflected in mobility, independence in activities of daily living (ADL), returning home (after admission to hospital or rehabilitation facility), or quality of life. Additional outcomes were facilitating factors and barriers to performing physical activity, and effects of the interventions on function and activity levels including strength, condition, balance, and the ability to walk.

*Text availability:* The full text of the articles was available, and the articles were written in the English language.

Simple case reports and reviews were excluded.

### Search strategy

**FIGURE 1:**
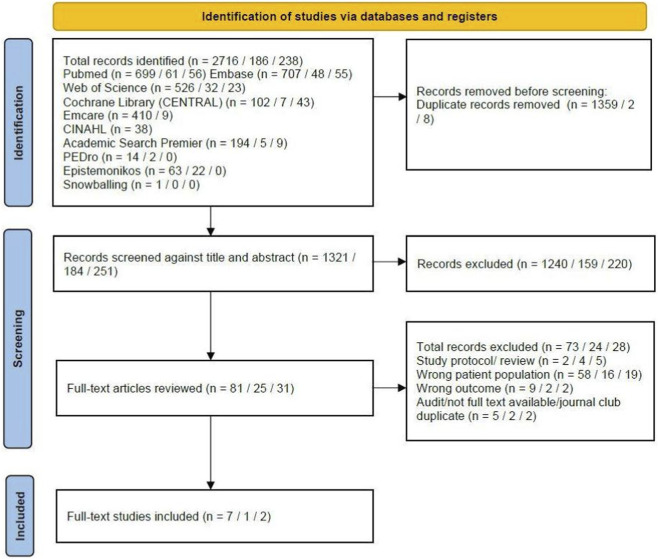
PRISMA flow diagram of the selection and inclusion of studies. Results are presented in n in 2023/n in 2024/n in 2025.

The first literature search was conducted on 3 August 2023, through PubMed, EMCARE, EMBASE, Web of Science, Cochrane Library, PEDro, Academic search premier and Epistemonikos and updated on 31 October 2024 (Figure 1). A second update was conducted on 16 December 2025 through the same databases except for EMCARE which was replaced by CINAHL (Figure 1). One researcher (EvG) and one librarian (JS) performed the searches. The search strategy consisted of a combination of Medical Subject Headings (MeSH) terms and relevant keywords. Those MeSH terms and keywords included and related to: “wheelchair”, “immobility”, “disuse”, “acutely ill”, “aged”, “elderly”, “physical activity” and “exercise”. See Supplementary Appendix 1 for the full electronic search strategy. The reference lists of relevant articles were screened to find additional relevant studies–i.e., backward snowballing.

### Study selection and data extraction

Figure 1 summarizes the search and selection process used in this review. The process was performed in exactly the same way during the first and the additional literature searches. Before screening the researchers performed a series of calibration exercises screening a small number of titles and abstracts. After duplicates were removed, two researchers (EvG and MV) independently screened all 2716 (first search), 186 (second search) and 251 (third search) titles and abstracts for eligibility of inclusion, using the online software Rayyan (Ouzzani et al., 2016). Articles that were considered potentially eligible by one or both researchers were read in full by one researcher (EvG), and screened for eligibility based on the inclusion and exclusion criteria. The full articles and results of this screening were discussed with the second researcher (MV) and studies that did not meet the inclusion criteria were excluded. Data extracted included: author; year of publication; study design; country; study setting; participants and reason for being non-ambulatory; minimal duration of non-ambulation; age; gender; sample size; intervention(s); outcome(s) of interest; other key findings relevant to the research question of this scoping review. The extracted data, including demographics, physical activity interventions, outcomes of interest and other key findings related to the research aim are summarized in Tables 1, 2, and in the results section below.

**TABLE 1:** Characteristics of the included studies.

First author (Year)	Study design	Country	Study setting	Participants and reasons for being non-ambulatory	Minimal duration of non-ambulation	Age	Gender	Sample size
Blower et al. (1995)	Observational study	England	Hospital	Stroke patients with hemiplegia unable to walk >3 weeks after onset	21 days	83% > 65	40% male	N = 52
Devries et al. (2015)	Experimental design	Canada	At home with tests and interventions at the hospital	Healthy men on step reduction	14 days	70 ± 1	100% male	N = 30
Fleury et al. (2025)	Pilot randomised controlled trial	Australian	Hospital	Patients with a lower limb fracture for which the orthopaedic surgical team have ordered NWB or TWB restriction	Unknown	IG High Freq 75.5 (69.0, 85.8) IG NMES 80.0 (66.5,83.0) IG Low Freq 74.0 (67.8,78.5)	37.5% male	N = 24
Hansen et al. (2024)	Experimental design	Denmark	Hospital	Healthy persons on bed rest	5 days	71.2 ± 3.3	50% male	N = 16
Hansen et al. (2025)	Experimental design	Denmark	Hospital	Healthy persons on bed rest	5 days	70.6 ± 3.0	57% male	N = 14
Hegerová et al. (2015)	Prospective randomized controlled study	Czech Republic	Hospital	Acutely ill patients during hospitalization	4 days	IG 83.6 ± 3.8 CG 83.2 ± 3.8	n/a	IG N = 100 CG N = 100
Lim and Kwek (2018)	Retrospective design	Singapore	Hospital	Patients with nonsurgically managed hip fractures during hospitalization	13 days	84 ± 1.89	25% male	N = 87
Moore et al. (2018)	Retrospective design	Canada	At home with tests and interventions at the hospital	Healthy men on step reduction	14 days	IG = 71 ± 5 CG = 69 ± 3	100% male	IG N = 14 CG N = 7
Nicholson et al. (1997)	Quasi-experimental, non-randomised control group pre-test/post-test design	South Africa	Hospital	IG: Patients admitted to inpatient rehabilitation CG: Patients discharged to their own residences	Unknown	IG 80 ± 6.2 CG 79.4 ± 7.8	0% male	IG = 20 CG = 10
Norheim et al. (2017)	Experimental design	Denmark	Hospital	Acutely ill patients during hospitalization	6 days	84.8 ± 1.9	50% male	N = 16

IG, Intervention group; CG, control group.

**TABLE 2:** Summary of physical activity interventions and frequency examined in the included studies.

First author (year)	Intervention and frequency
Blower et al. (1995)	IG: Wheelchair propulsion. Frequency was not described
Devries et al. (2015)	IG (L): Step reduction and leg press and leg extension using compressed air resistance strength machines. 3 days a week, 3 sets CG (L): Step reduction
Fleury et al. (2025)	Low frequency physiotherapy (Standard Care): Both intervention and control groups received routine care. Individualised lower-limb exercise program to be performed independently 3 times a day, plus weekly guided physiotherapy/allied health assistant session program (in lying and seated positions). Exercises: 3 sets of 10–15 RM on the unaffected leg with progressive loading; affected leg exercises modified with no loading to follow fracture-healing principles High frequency physiotherapy: Low frequency physiotherapy plus an additional three physiotherapist/AHA contacts, so participants received supervised exercises for 4 sessions, in total per week Neuromuscular electric stimulation: Low frequency physiotherapy plus four supervised NMES per week (delivered over 4 days) to both affected and unaffected quadriceps for 25 min each leg
Hansen et al. (2024)	IG (L): Bed rest and neuromuscular electric stimulation. 3 times daily. Comprising 2 min warm-up phase and 30 min work phase CG (L): Bed rest
Hansen et al. (2025)	IG (L): Bed rest and neuromuscular electric stimulation. 3 times daily. Comprising 2 min warm-up phase and 30 min work phase CG (L): Bed rest
Hegerová et al. (2015)	IG: Physical training focused on increase of proprioception, maintaining joint flexibility, training neuromuscular coordination, support of respiration, training capability to maintain balance, and gait training twice a day for 15 min + twice a day 5 min aerobic exercises on the bicycle ergometer CG: According to the standard protocol in the department, physical training 10–15 min 5 days a week without aerobic exercise
Lim and Kwek (2018)	IG: Early wheelchair mobilization protocol, mobilization with assistance as soon as possible after admission. Frequency was not described
Moore et al. (2018)	IG (L): Step reduction and leg press and leg extension using compressed air resistance strength machines. 3 days a week 3 sets CG (L): Step reduction
Nicholson et al. (1997)	IG: Chair exercise. 50 min with 10 min warm-up. The emphasis was on muscle endurance and range of movement. 4 days a week CG: NA: Patients who were discharged to their own residences
Norheim et al. (2017)	IG (L): Leg press machine. 3 to 6 sets of 10–12 repetitions 7 days a week CG (L): No intervention

IG, intervention group; CG, control group, (L) = leg.

## Results

### Study selection and study characteristics

In total, 3140 articles were retrieved, and after removing duplicates, 1756 titles and abstracts were reviewed by two independent reviewers. After screening, 137 articles were selected for full-text review. A total of ten studies were found to be eligible and were included in the analyses (Figure 1). Nine of these studies were found through a literature search in various online databases (Blower et al., 1995; Hegerová et al., 2015; Lim and Kwek, 2018; Moore et al., 2018; Nicholson et al., 1997; Norheim et al., 2017; Hansen et al., 2024; Fleury et al., 2025; Hansen et al., 2025). By searching the reference list of these nine studies (i.e., backward snowballing), one additional study was identified (Devries et al., 2015). The characteristics of the included studies are summarized in Table 1. The studies were published between 1995 and 2025 and conducted in the continents Afrika, Europe, North America, Asia and Oceania. The study-designs used were; observational design (Brown et al., 2021), (quasi) experimental design (Rejc et al., 2018), prospective randomized controlled design (Brown et al., 2021) and retrospective design (Tanner et al., 2015). Four studies included healthy older persons who were experimental non-ambulant, and were asked to reduce their steps to <1500 for 14 days or to 0 for 5 days (Moore et al., 2018; Hansen et al., 2024; Hansen et al., 2025; Devries et al., 2015). The other studies included patients who were temporarily unable to ambulate due to one of the following medical conditions: lower limb fracture, hip fracture, stroke or not specified acutely ill or not acutely ill but hospitalized.

### Physical activity interventions and its effects on physical function and activity levels

Most studies included some form of strength training for the lower or upper extremities (Table 2). Two studies only focused on wheelchair mobilization (Blower et al., 1995; Lim and Kwek, 2018). Wheelchair mobilization includes sitting up in a wheelchair and being able to self manually propel a wheelchair. Of the ten studies, five studies compared legs within the participants as one leg received the intervention (intervention leg) and the other leg not (control leg), three compared an intervention with an control group and two observed one group for a period of time.

We found four main types of physical activity interventions for older persons who are temporarily non-ambulant;

1. Sitting up and wheelchair mobilization;
2. Strength training of the lower extremity;
3. (seated) physical activity programme including multiple types of physical activity.
4. Neuromuscular electric stimulation

#### Sitting up andhla wheelchair mobilization (two studies)

Blower et al. (1995) found that the ability to propel a wheelchair is related to the ability to walk post-stroke in older persons (Blower et al., 1995). The study by Lim and Kwek (2018) showed that fewer days required to sit up in bed was significantly associated with the ability to ambulate (Lim and Kwek, 2018). Moreover, pain was not a barrier to achieving ambulation milestones after non-surgical management of hip fracture in older persons (Lim and Kwek, 2018).

#### Strength training of the lower extremity (three studies)

Three studies examined the effect of strength training on one lower extremity compared to the other lower extremity while participants were non-ambulant. In the study of Devries et al. (2015) and Moore et al. (2018) healthy participants were asked to be temporarily non-ambulant for purpose of the study, whereas in Norheim et al. (2017) the participants were temporarily non-ambulant due to acute illnesses (Moore et al., 2018; Norheim et al., 2017; Devries et al., 2015). Devries et al. (2015) and Norheim et al. (2017) found no effect of strength training on isometric knee extension (Norheim et al., 2017; Devries et al., 2015). Additionally, Norheim et al. (2017) reported no effect on physical function, as measured by the 30-s chair stand test and the 4-m gait speed test, or physical activity (Norheim et al., 2017). However, both studies did find positive effects, including an increase in one repetition maximum, a main effect of time on leg extension power, and a increased training load. Muscle mass also increased in the intervention leg compared to the control leg, and isotonic knee extensor strength improved in both the intervention and control legs (Norheim et al., 2017; Devries et al., 2015). Furthermore, Moore et al. (2018) found a significant increase in muscle fibre types I and II in the intervention leg compared to the control leg (Moore et al., 2018).

#### (Seated) physical activity programme including multiple types of physical activity (two studies)

In the study by Heragová et al. (2015), both intervention and control groups showed a decline in physical activity, as measured by the Barthel index. However, the decline occurred after 3 months in the control group, whereas in the intervention group, it took more than 12 months for a decline to occur (Hegerová et al., 2015). No changes were found in anthropometric measurements, such as body weight, body mass index, and lean body mass (Hegerová et al., 2015).

There was an improvement in fear of falling, as measured by the Falls Efficacy Scale, in both the intervention and control group observed in the study by Nicholson et al. (1997). However, even though both groups showed an improvement between the baseline measurement and 6 weeks alter, there were no differences in fear of falling between the two groups (Nicholson et al., 1997). Additionally, there were no changes in strength, range of motion, or lung function between the two groups. Differences in anthropometry, such as mass, waist circumference and calf skinfold, were observed in this study post-intervention. However, differences in anthropometry between the groups already existed pre-intervention, which may have obscured the effect of the exercise intervention (Nicholson et al., 1997). Compliance in the experimental group was high and this was believed to be due to possible social benefits of the group format as well as possible mood-elevating effect of the exercise (Nicholson et al., 1997).

#### Neuromuscular electric stimulation (three studies)

Across the included studies, NMES showed potential to attenuate muscle loss during periods of immobility. The two studies of Hansen et al. (2024) included healthy older persons who were experimental non-ambulant, whereas the study of Fleury et al. (2025) included Patients with a lower limb fracture for which the orthopaedic surgical team have ordered NWB or TWB restriction (Hansen et al., 2024; Fleury et al., 2025; Hansen et al., 2025). Hansen et al. (2024) demonstrated that 5 days of NMES during bed rest increased vastus lateralis muscle thickness in the stimulated leg, while thickness declined in the non-stimulated leg; no effects were found on muscle strength (Hansen et al., 2024). Similar findings were reported in another study by Hansen et al. (2025), where NMES preserved voluntary muscle activation but did not prevent declines in maximal voluntary strength or evoked twitch forces during bed rest (Hansen et al., 2025). No signs of neuromuscular junction instability or muscle damage resulting from bedrest or NMES were identified (Hansen et al., 2025).

Therapists participating in the study of Fleury et al. (2025) delivering the intervention found NMES easy to administer after training, although staffing shortages and clinical workload made consistent delivery of the interventions challenging (Fleury et al., 2025). Although the study by Fleury et al. (2025) was not powered to detect significant differences, findings suggest that NMES and high-frequency physiotherapy may support the maintenance of muscle strength in older persons during periods of restricted weight bearing after a lower-limb fracture (Fleury et al., 2025). In addition, the study reported that both interventions are feasible to deliver in this population.

### Safety

The results reported in the included studies suggest that engaging in physical activity appears to be safe for older persons who are temporarily clinically non-ambulant. Only Nicholson et al. (1997) reported that a participant withdrew from a single exercise session due to vertigo (Nicholson et al., 1997). Lim and Kwek (2018) found that participants who reached ambulation milestones earlier took longer to become pain-free. Which, as they reported in their study, could have been caused by the therapy sessions and ambulation (Lim and Kwek, 2018).

## Discussion

This scoping review was conducted to provide an overview of physical activity interventions for older persons who are temporarily non-ambulant, and of the effects of those interventions on physical function. This review indicates that physical activity in a sitting or lying position has positive effects on strength and is associated with the ability to walk again. These effects can be achieved through small interventions such as sitting up, but can also include strength training of the lower extremity, neuromuscular electrical stimulation or seated aerobic or balance exercises. However, no improvements were found in physical function at the level of activities of daily living. In clinical practice this does mean stimulating physical activity as early as possible–even sitting up is better as lying down–ideally in a group format and if possible supplemented with neuromuscular electrical stimulation. Additionally, there are only a small number of studies focusing on physical activities for these temporarily non-ambulant older persons. This is remarkable, as being temporarily non-ambulant is prevalent and multiple studies have shown that temporary immobility has adverse health effects.

One could discuss whether small interventions such as sitting up can be described as “being physical active”. The World Health Organization guidelines on physical activity and sedentary behaviour (2020) defines physical activity as “A*ny bodily movement produced by skeletal muscles that requires energy expenditure*.”, and sedentary behaviour as “A*ny waking behaviour characterized by an energy expenditure of 1.5 metabolic equivalent or lower while sitting, reclining, or lying*.*”*. However, for someone who has a poor condition, for instance due to disease related immobility and prolonged bedrest, small interventions like sitting up might exceed the 1.5 metabolic equivalent (Verschuren et al., 2016). Therefore it could be argued that whether an activity qualifies as physical activity depends on the individual’s condition. Another point of discussion could be whether using NMES falls under being physical active. However, neuromuscular electric stimulation (NMES) can produce a muscle contraction of 20%–40% of a maximum voluntary contraction. Which fulfils the American College of Sports Medicine’s definition of exercise: “*A planned, structured and repetitive bodily movement done to improve or maintain one or more components of physical fitness*.” (Jones et al., 2016; Maffiuletti, 2010).

Studies in younger persons with temporary restrictions in weight bearing on their lower extremity, have shown that early non-weight-bearing mobilization and strength training of the lower extremity can prevent muscle atrophy and weakness, and improve earlier functional outcomes (Henkelmann et al., 2021; Jansen et al., 2018; Siddique et al., 2005; Taufik et al., 2019; Vioreanu et al., 2007). Seated physical activity programmes for older persons who are permanently non-ambulant, such as cycling, elastic band exercise programmes or Tai Chi, have been shown to significantly improve quality of life. Additionally, these programmes have a positive impact on aerobic capacity, depression, mobility, strength, overall functional fitness and sleep quality (Chen et al., 2015; Chen et al., 2016; Fitzsimmons, 2001; Fu et al., 2022; Miura et al., 2012; Mulrow et al., 1994; Wang et al., 2021; Wang et al., 2016; Wołoszyn et al., 2021). These studies show that physical activity positively impacts not only physical but also mental function, in both younger individuals with temporary immobility and permanently non-ambulant older persons. However, lessons learned in younger persons cannot be assumed to work similarly for older persons as older persons react differently to immobility and retraining (Rejc et al., 2018; Suetta et al., 2009). Additionally, permanent non-ambulant older persons may react differently to physical activity than temporarily non-ambulant older persons. The condition between permanent, and temporarily non-ambulant older persons who until recently have been ambulant, might be different and not comparable at the start of the intervention. Although the results of these studies may not be directly transferable to older persons who are temporarily non-ambulant, their methods and physical activity interventions can offer valuable insights for developing physical activity guidelines for this population. Additionally, results from this study indicate that both older persons suffering from acute illness as healthy older persons react similarly on strength training of the lower extremity during a period of non-ambulation as shown by the similarities in outcomes between the study of Devries et al. (2015) and Norheim et al. (2017) (Norheim et al., 2017; Devries et al., 2015).

This scoping review revealed that only a limited number of studies specifically addressed physical activity interventions for older persons who are temporarily non-ambulant. The limited evidence base, experimental disuse models, and relatively small sample sizes in the included studies highlight the need for further research with larger, well-defined cohorts. Such studies would allow for more robust conclusions and improve generalizability. The scoping review approach enabled comprehensive coverage of the available literature, including different types of physical activity interventions, outcomes, and reasons for which older persons are temporarily non-ambulant and was appropriate given the limited number of studies. However, this resulted in heterogeneity in both interventions and outcome measures, which limits comparability. We included both older persons who were temporarily clinically or experimental non-ambulant, even though the results between both groups showed similarities, including studies with healthy older persons might limit generalizability of the results to clinical populations. We deliberately excluded studies including persons under 60 years, as their physiological response to immobility and retraining differs substantially from older persons. While this ensured relevance to our target population, it may have resulted in overlooking potentially useful insights from related research (Fossat et al., 2018). Future research should therefore focus on high-quality designs and larger participant groups to strengthen the evidence base for physical activity interventions in this vulnerable population.

This review highlights the need for future research into how we can improve physical activity in temporarily non-ambulant older persons. Based on the findings, we recommend promoting physical activity as early as possible, even in sitting or lying position, to increase strength and facilitate ambulation, ideally in a group format. To prevent adverse health effects of immobility, further research is needed to identify which activities older persons who are temporarily non-ambulant can and are motivated to perform, and how these activities influence physical function and rehabilitation outcomes.
