# Supplementary material for: Physical activity interventions to improve physical function in temporarily non-ambulant older persons: a scoping review
Source: Front Aging. 2026 Apr 15;7:1816647. doi: 10.3389/fragi.2026.1816647 (PMC13125031; doi:10.3389/fragi.2026.1816647)
Supplement: Supplementary file 1 [file Table1.docx]

**Appendix 1:**

**Search strategy**

The literature search was conducted through PubMed, EMCARE, EMBASE, Web of Science, Cochrane Library, PEDro, Academic search premier, Epistemonikos and CINAHL.

- Text availability: The full text of the articles was available, and the articles were written in the English language.
- Simple case reports and reviews were excluded.

Wheelchairs AND physical activity AND Lower extremity Fractures AND acutely ill AND disuse AND immobility AND **elderly**

**PubMed**

**(**(("Wheelchairs"[Mesh] OR "Wheelchairs"[tw] OR "Wheelchair"[tw] OR "Wheelchair*"[tw] OR "Wheel chairs"[tw] OR "Wheel chair"[tw] OR "Wheel chair*"[tw] OR "Mobility Scooter"[tw] OR "Mobility Scooters"[tw]) AND ("Exercise"[Mesh] OR "Exercise"[tw] OR "Exercises"[tw] OR "Exercising"[tw] OR "Exercis*"[tw] OR "physical activity"[tw] OR "physical activities"[tw] OR "Endurance Training"[tw] OR "Exergaming"[tw] OR "Gymnastics"[tw] OR "Gymnastic"[tw] OR "Interval Training"[tw] OR "Jogging"[tw] OR "Motor Activity"[tw] OR "Movement"[tw] OR "Muscle Stretching "[tw] OR "Physical Conditioning"[tw] OR "Resistance Training"[tw] OR "Running"[tw] OR "Stair Climbing"[tw] OR "Swimming"[tw] OR "Training"[tw] OR "Walking"[tw] OR "Exercise Therapy"[mesh] OR "Physical Exertion"[mesh] OR "Physical Exertion"[tw] OR "Physical Exertions"[tw] OR "Physical Effort"[tw] OR "Physical Efforts"[tw] OR "Sports"[mesh] OR "Sports"[tw] OR "Sport"[tw] OR "Athletic Performance"[tw] OR "Baseball"[tw] OR "Basketball"[tw] OR "Bicycling"[tw] OR "Boxing"[tw] OR "Cardiorespiratory Fitness"[tw] OR "Cardiorespiratory Fitness"[tw] OR "Cricket Sport"[tw] OR "Diving"[tw] OR "Football"[tw] OR "Golf"[tw] OR "Gymnastics"[tw] OR "Hockey"[tw] OR "Jogging"[tw] OR "Marathon Running"[tw] OR "Martial Arts"[tw] OR "Mountaineering"[tw] OR "Nordic Walking"[tw] OR "Physical Endurance"[tw] OR "Physical Fitness"[tw] OR "Racquet Sports"[tw] OR "Return to Sport"[tw] OR "Rugby"[tw] OR "Running"[tw] OR "Skating"[tw] OR "Skiing"[tw] OR "Snow Sports"[tw] OR "Soccer"[tw] OR "Swimming"[tw] OR "Tai Ji"[tw] OR "Team Sports"[tw] OR "Tennis"[tw] OR "Track and Field"[tw] OR "Volleyball"[tw] OR "Walking"[tw] OR "Water Sports"[tw] OR "Weight Lifting"[tw] OR "Wrestling"[tw] OR "Youth Sports"[tw] OR "Exercise Movement Techniques"[mesh] OR "Qigong"[tw] OR "Dance Therapy"[tw] OR "Tai Ji"[tw] OR "Yoga"[tw] OR "Physical Therapy Modalities"[Mesh:noexp] OR "Physical Therapy"[tw] OR "physiotherapy"[tw] OR "physiotherapy*"[tw] OR "Dry Needling"[mesh] OR "Electric Stimulation Therapy"[mesh] OR "Extracorporeal Shockwave Therapy"[mesh] OR "Hydrotherapy"[mesh] OR "Musculoskeletal Manipulations"[mesh] OR "Dry Needling"[tw] OR "Electric Stimulation Therapy"[tw] OR "Electroacupuncture"[tw] OR "Pulsed Radiofrequency Treatment"[tw] OR "Spinal Cord Stimulation"[tw] OR "Transcutaneous Electric Nerve Stimulation"[tw] OR "Extracorporeal Shockwave Therapy"[tw] OR "Hydrotherapy"[tw] OR "Aquatic Therapy"[tw] OR "Therapeutic Irrigation"[tw] OR "Musculoskeletal Manipulations"[tw] OR "Applied Kinesiology"[tw] OR "Orthopedic Manipulation"[tw] OR "Orthopaedic Manipulation"[tw] OR "Osteopathic Manipulation"[tw] OR "Spinal Manipulation"[tw] OR "Continuous Passive Motion Therapy"[tw] OR "Soft Tissue Therapy"[tw] OR "Acupressure"[tw] OR "Massage"[tw] OR "Manual Lymphatic Drainage"[tw] OR "Myofascial Release Therapy"[tw] OR "mobilization"[tw] OR "mobilisation"[tw] OR "mobilizing"[tw] OR "mobilising"[tw] OR "mobilize"[tw] OR "mobilise"[tw] OR "mobilized"[tw] OR "mobilised"[tw] OR "mobility"[tw]) AND ("Ankle Fractures"[mesh] OR "Femoral Fractures"[mesh] OR "Fibula Fractures"[mesh] OR "Knee Fractures"[mesh] OR "Tibial Fractures"[mesh] OR "Ankle Fractures"[tw] OR "Femoral Fractures"[tw] OR "Hip Fractures"[tw] OR "Femoral Neck Fractures"[tw] OR "Proximal Femoral Fractures"[tw] OR "Hoffa Fracture"[tw] OR "Proximal Femoral Fractures"[tw] OR "Fibula Fractures"[tw] OR "Knee Fractures"[tw] OR "Hoffa Fracture"[tw] OR "Patella Fracture"[tw] OR "Tibial Plateau Fractures"[tw] OR "Tibial Fractures"[tw] OR "Tibial Plateau Fractures"[tw] OR "Ankle Fracture"[tw] OR "Femoral Fracture"[tw] OR "Hip Fracture"[tw] OR "Femoral Neck Fracture"[tw] OR "Proximal Femoral Fracture"[tw] OR "Hoffa Fractures"[tw] OR "Proximal Femoral Fracture"[tw] OR "Fibula Fracture"[tw] OR "Knee Fracture"[tw] OR "Hoffa Fractures"[tw] OR "Patella Fractures"[tw] OR "Tibial Plateau Fracture"[tw] OR "Tibial Fracture"[tw] OR "Tibial Plateau Fracture"[tw] OR (("Lower Extremity"[Mesh] OR "lower limb"[tw] OR "lower limbs"[tw] OR "lower extremity"[tw] OR "lower extremities"[tw] OR "Ankle"[tw] OR "Foot"[tw] OR "Heel"[tw] OR "Hip"[tw] OR "Knee"[tw] OR "Leg"[tw] OR "Ankles"[tw] OR "Feet"[tw] OR "Heels"[tw] OR "Hips"[tw] OR "Knees"[tw] OR "Legs"[tw]) AND ("Fractures, Bone"[Mesh] OR "fracture"[tw] OR "fractures"[tw] OR "fractur*"[tw])) OR "immobility"[tw] OR "immobile"[tw] OR "immobil*"[tw] OR "temporary"[tw] OR "non weight bearing"[tw] OR "nonweight bearing"[tw] OR "non weight bear*"[tw] OR "nonweight bear*"[tw])**) OR** (("Wheelchairs"[majr] OR "Wheelchairs"[ti] OR "Wheelchair"[ti] OR "Wheelchair*"[ti] OR "Wheel chairs"[ti] OR "Wheel chair"[ti] OR "Wheel chair*"[ti] OR "Mobility Scooter"[ti] OR "Mobility Scooters"[ti]) AND ("Exercise"[majr] OR "Exercise"[ti] OR "Exercises"[ti] OR "Exercising"[ti] OR "Exercis*"[ti] OR "physical activity"[ti] OR "physical activities"[ti] OR "Endurance Training"[ti] OR "Exergaming"[ti] OR "Gymnastics"[ti] OR "Gymnastic"[ti] OR "Interval Training"[ti] OR "Jogging"[ti] OR "Motor Activity"[ti] OR "Movement"[ti] OR "Muscle Stretching "[ti] OR "Physical Conditioning"[ti] OR "Resistance Training"[ti] OR "Running"[ti] OR "Stair Climbing"[ti] OR "Swimming"[ti] OR "Training"[ti] OR "Walking"[ti] OR "Exercise Therapy"[majr] OR "Physical Exertion"[majr] OR "Physical Exertion"[ti] OR "Physical Exertions"[ti] OR "Physical Effort"[ti] OR "Physical Efforts"[ti] OR "Sports"[majr] OR "Sports"[ti] OR "Sport"[ti] OR "Athletic Performance"[ti] OR "Baseball"[ti] OR "Basketball"[ti] OR "Bicycling"[ti] OR "Boxing"[ti] OR "Cardiorespiratory Fitness"[ti] OR "Cardiorespiratory Fitness"[ti] OR "Cricket Sport"[ti] OR "Diving"[ti] OR "Football"[ti] OR "Golf"[ti] OR "Gymnastics"[ti] OR "Hockey"[ti] OR "Jogging"[ti] OR "Marathon Running"[ti] OR "Martial Arts"[ti] OR "Mountaineering"[ti] OR "Nordic Walking"[ti] OR "Physical Endurance"[ti] OR "Physical Fitness"[ti] OR "Racquet Sports"[ti] OR "Return to Sport"[ti] OR "Rugby"[ti] OR "Running"[ti] OR "Skating"[ti] OR "Skiing"[ti] OR "Snow Sports"[ti] OR "Soccer"[ti] OR "Swimming"[ti] OR "Tai Ji"[ti] OR "Team Sports"[ti] OR "Tennis"[ti] OR "Track and Field"[ti] OR "Volleyball"[ti] OR "Walking"[ti] OR "Water Sports"[ti] OR "Weight Lifting"[ti] OR "Wrestling"[ti] OR "Youth Sports"[ti] OR "Exercise Movement Techniques"[majr] OR "Qigong"[ti] OR "Dance Therapy"[ti] OR "Tai Ji"[ti] OR "Yoga"[ti] OR "Physical Therapy Modalities"[majr:noexp] OR "Physical Therapy"[ti] OR "physiotherapy"[ti] OR "physiotherapy*"[ti] OR "Dry Needling"[majr] OR "Electric Stimulation Therapy"[majr] OR "Extracorporeal Shockwave Therapy"[majr] OR "Hydrotherapy"[majr] OR "Musculoskeletal Manipulations"[majr] OR "Dry Needling"[ti] OR "Electric Stimulation Therapy"[ti] OR "Electroacupuncture"[ti] OR "Pulsed Radiofrequency Treatment"[ti] OR "Spinal Cord Stimulation"[ti] OR "Transcutaneous Electric Nerve Stimulation"[ti] OR "Extracorporeal Shockwave Therapy"[ti] OR "Hydrotherapy"[ti] OR "Aquatic Therapy"[ti] OR "Therapeutic Irrigation"[ti] OR "Musculoskeletal Manipulations"[ti] OR "Applied Kinesiology"[ti] OR "Orthopedic Manipulation"[ti] OR "Orthopaedic Manipulation"[ti] OR "Osteopathic Manipulation"[ti] OR "Spinal Manipulation"[ti] OR "Continuous Passive Motion Therapy"[ti] OR "Soft Tissue Therapy"[ti] OR "Acupressure"[ti] OR "Massage"[ti] OR "Manual Lymphatic Drainage"[ti] OR "Myofascial Release Therapy"[ti] OR "mobilization"[ti] OR "mobilisation"[ti] OR "mobilizing"[ti] OR "mobilising"[ti] OR "mobilize"[ti] OR "mobilise"[ti] OR "mobilized"[ti] OR "mobilised"[ti] OR "mobility"[ti]) AND **("Aged"[mesh] OR "elderly"[all fields] OR "elder"[all fields] OR "elders"[all fields] OR "geriatr*"[all fields] OR "Homes for the Aged"[mesh] OR "Health Services for the Aged"[mesh] OR "Senior Centers"[mesh] OR "Nonagenarians"[all fields] OR "Nonagenarian"[all fields] OR "Octogenarians"[all fields] OR "Octogenarian"[all fields] OR "Centenarians"[all fields] OR "Centenarian"[all fields] OR "septuagenarian"[all fields] OR "septuagenarians"[all fields] OR "Aging"[mesh] OR "aging"[all fields] OR "ageing"[all fields] OR "geront*"[all fields] OR "older person"[all fields] OR "old person"[all fields] OR "older patient"[all fields] OR "old patient"[all fields] OR "older persons"[all fields] OR "old persons"[all fields] OR "older patients"[all fields] OR "old patients"[all fields] OR "older women"[all fields] OR "old women"[all fields] OR "older men"[all fields] OR "old men"[all fields] OR "old adult"[all fields] OR "older adult"[all fields] OR "old adults"[all fields] OR "older adults"[all fields] OR "Older individual"[all fields] OR "Older individuals"[all fields] OR "old people"[all fields] OR "older people"[all fields] OR "Oldest Old"[all fields] OR "older population"[all fields] OR "aging population"[all fields] OR "aging population"[all fields] OR "old aged"[all fields] OR "old age"[all fields] OR "older person"[title/abstract:~3] OR "old person"[title/abstract:~3] OR "older patient"[title/abstract:~3] OR "old patient"[title/abstract:~3] OR "older persons"[title/abstract:~3] OR "old persons"[title/abstract:~3] OR "older patients"[title/abstract:~3] OR "old patients"[title/abstract:~3] OR "older women"[title/abstract:~3] OR "old women"[title/abstract:~3] OR "older men"[title/abstract:~3] OR "old men"[title/abstract:~3] OR "old adult"[title/abstract:~3] OR "older adult"[title/abstract:~3] OR "old adults"[title/abstract:~3] OR "older adults"[title/abstract:~3] OR "Older individual"[title/abstract:~3] OR "Older individuals"[title/abstract:~3] OR "old people"[title/abstract:~3] OR "older people"[title/abstract:~3] OR "Oldest Old"[title/abstract:~3] OR "older population"[title/abstract:~3] OR "aging population"[title/abstract:~3] OR "aging population"[title/abstract:~3] OR "old aged"[title/abstract:~3] OR "old age"[title/abstract:~3] OR** "60 year old"[title/abstract:~3] OR "61 year old"[title/abstract:~3] OR "62 year old"[title/abstract:~3] OR "63 year old"[title/abstract:~3] OR "64 year old"[title/abstract:~3] OR "65 year old"[title/abstract:~3] OR "66 year old"[title/abstract:~3] OR "67 year old"[title/abstract:~3] OR "68 year old"[title/abstract:~3] OR "69 year old"[title/abstract:~3] OR "60 years old"[title/abstract:~3] OR "61 years old"[title/abstract:~3] OR "62 years old"[title/abstract:~3] OR "63 years old"[title/abstract:~3] OR "64 years old"[title/abstract:~3] OR "65 years old"[title/abstract:~3] OR "66 years old"[title/abstract:~3] OR "67 years old"[title/abstract:~3] OR "68 years old"[title/abstract:~3] OR "69 years old"[title/abstract:~3] OR "70 year old"[title/abstract:~3] OR "71 year old"[title/abstract:~3] OR "72 year old"[title/abstract:~3] OR "73 year old"[title/abstract:~3] OR "74 year old"[title/abstract:~3] OR "75 year old"[title/abstract:~3] OR "76 year old"[title/abstract:~3] OR "77 year old"[title/abstract:~3] OR "78 year old"[title/abstract:~3] OR "79 year old"[title/abstract:~3] OR "70 years old"[title/abstract:~3] OR "71 years old"[title/abstract:~3] OR "72 years old"[title/abstract:~3] OR "73 years old"[title/abstract:~3] OR "74 years old"[title/abstract:~3] OR "75 years old"[title/abstract:~3] OR "76 years old"[title/abstract:~3] OR "77 years old"[title/abstract:~3] OR "78 years old"[title/abstract:~3] OR "79 years old"[title/abstract:~3] OR "80 year old"[title/abstract:~3] OR "81 year old"[title/abstract:~3] OR "82 year old"[title/abstract:~3] OR "83 year old"[title/abstract:~3] OR "84 year old"[title/abstract:~3] OR "85 year old"[title/abstract:~3] OR "86 year old"[title/abstract:~3] OR "87 year old"[title/abstract:~3] OR "88 year old"[title/abstract:~3] OR "89 year old"[title/abstract:~3] OR "80 years old"[title/abstract:~3] OR "81 years old"[title/abstract:~3] OR "82 years old"[title/abstract:~3] OR "83 years old"[title/abstract:~3] OR "84 years old"[title/abstract:~3] OR "85 years old"[title/abstract:~3] OR "86 years old"[title/abstract:~3] OR "87 years old"[title/abstract:~3] OR "88 years old"[title/abstract:~3] OR "89 years old"[title/abstract:~3] OR "90 year old"[title/abstract:~3] OR "91 year old"[title/abstract:~3] OR "92 year old"[title/abstract:~3] OR "93 year old"[title/abstract:~3] OR "94 year old"[title/abstract:~3] OR "95 year old"[title/abstract:~3] OR "96 year old"[title/abstract:~3] OR "97 year old"[title/abstract:~3] OR "98 year old"[title/abstract:~3] OR "99 year old"[title/abstract:~3] OR "90 years old"[title/abstract:~3] OR "91 years old"[title/abstract:~3] OR "92 years old"[title/abstract:~3] OR "93 years old"[title/abstract:~3] OR "94 years old"[title/abstract:~3] OR "95 years old"[title/abstract:~3] OR "96 years old"[title/abstract:~3] OR "97 years old"[title/abstract:~3] OR "98 years old"[title/abstract:~3] OR "99 years old"[title/abstract:~3] OR "100 year old"[title/abstract:~3] OR "101 year old"[title/abstract:~3] OR "102 year old"[title/abstract:~3] OR "103 year old"[title/abstract:~3] OR "104 year old"[title/abstract:~3] OR "105 year old"[title/abstract:~3] OR "106 year old"[title/abstract:~3] OR "107 year old"[title/abstract:~3] OR "108 year old"[title/abstract:~3] OR "109 year old"[title/abstract:~3] OR "100 years old"[title/abstract:~3] OR "101 years old"[title/abstract:~3] OR "102 years old"[title/abstract:~3] OR "103 years old"[title/abstract:~3] OR "104 years old"[title/abstract:~3] OR "105 years old"[title/abstract:~3] OR "106 years old"[title/abstract:~3] OR "107 years old"[title/abstract:~3] OR "108 years old"[title/abstract:~3] OR "109 years old"[title/abstract:~3]**)) OR** (("Exercise"[majr] OR "Exercise"[tiab] OR "Exercises"[tiab] OR "Exercising"[tiab] OR "Exercis*"[tiab] OR "physical activity"[tiab] OR "physical activities"[tiab] OR "Endurance Training"[tiab] OR "Exergaming"[tiab] OR "Gymnastics"[tiab] OR "Gymnastic"[tiab] OR "Interval Training"[tiab] OR "Jogging"[tiab] OR "Motor Activity"[tiab] OR "Movement"[tiab] OR "Muscle Stretching "[tiab] OR "Physical Conditioning"[tiab] OR "Resistance Training"[tiab] OR "Running"[tiab] OR "Stair Climbing"[tiab] OR "Swimming"[tiab] OR "Training"[tiab] OR "Walking"[tiab] OR "Exercise Therapy"[majr] OR "Physical Exertion"[majr] OR "Physical Exertion"[tiab] OR "Physical Exertions"[tiab] OR "Physical Effort"[tiab] OR "Physical Efforts"[tiab] OR "Sports"[majr] OR "Sports"[tiab] OR "Sport"[tiab] OR "Athletic Performance"[tiab] OR "Baseball"[tiab] OR "Basketball"[tiab] OR "Bicycling"[tiab] OR "Boxing"[tiab] OR "Cardiorespiratory Fitness"[tiab] OR "Cardiorespiratory Fitness"[tiab] OR "Cricket Sport"[tiab] OR "Diving"[tiab] OR "Football"[tiab] OR "Golf"[tiab] OR "Gymnastics"[tiab] OR "Hockey"[tiab] OR "Jogging"[tiab] OR "Marathon Running"[tiab] OR "Martial Arts"[tiab] OR "Mountaineering"[tiab] OR "Nordic Walking"[tiab] OR "Physical Endurance"[tiab] OR "Physical Fitness"[tiab] OR "Racquet Sports"[tiab] OR "Return to Sport"[tiab] OR "Rugby"[tiab] OR "Running"[tiab] OR "Skating"[tiab] OR "Skiing"[tiab] OR "Snow Sports"[tiab] OR "Soccer"[tiab] OR "Swimming"[tiab] OR "Tai Ji"[tiab] OR "Team Sports"[tiab] OR "Tennis"[tiab] OR "Track and Field"[tiab] OR "Volleyball"[tiab] OR "Walking"[tiab] OR "Water Sports"[tiab] OR "Weight Lifting"[tiab] OR "Wrestling"[tiab] OR "Youth Sports"[tiab] OR "Exercise Movement Techniques"[majr] OR "Qigong"[tiab] OR "Dance Therapy"[tiab] OR "Tai Ji"[tiab] OR "Yoga"[tiab] OR "Physical Therapy Modalities"[majr:noexp] OR "Physical Therapy"[tiab] OR "physiotherapy"[tiab] OR "physiotherapy*"[tiab] OR "Dry Needling"[majr] OR "Electric Stimulation Therapy"[majr] OR "Extracorporeal Shockwave Therapy"[majr] OR "Hydrotherapy"[majr] OR "Musculoskeletal Manipulations"[majr] OR "Dry Needling"[tiab] OR "Electric Stimulation Therapy"[tiab] OR "Electroacupuncture"[tiab] OR "Pulsed Radiofrequency Treatment"[tiab] OR "Spinal Cord Stimulation"[tiab] OR "Transcutaneous Electric Nerve Stimulation"[tiab] OR "Extracorporeal Shockwave Therapy"[tiab] OR "Hydrotherapy"[tiab] OR "Aquatic Therapy"[tiab] OR "Therapeutic Irrigation"[tiab] OR "Musculoskeletal Manipulations"[tiab] OR "Applied Kinesiology"[tiab] OR "Orthopedic Manipulation"[tiab] OR "Orthopaedic Manipulation"[tiab] OR "Osteopathic Manipulation"[tiab] OR "Spinal Manipulation"[tiab] OR "Continuous Passive Motion Therapy"[tiab] OR "Soft Tissue Therapy"[tiab] OR "Acupressure"[tiab] OR "Massage"[tiab] OR "Manual Lymphatic Drainage"[tiab] OR "Myofascial Release Therapy"[tiab] OR "mobilization"[tiab] OR "mobilisation"[tiab] OR "mobilizing"[tiab] OR "mobilising"[tiab] OR "mobilize"[tiab] OR "mobilise"[tiab] OR "mobilized"[tiab] OR "mobilised"[tiab] OR "mobility"[tiab]) AND ("Ankle Fractures"[majr] OR "Femoral Fractures"[majr] OR "Fibula Fractures"[majr] OR "Knee Fractures"[majr] OR "Tibial Fractures"[majr] OR "Ankle Fractures"[ti] OR "Femoral Fractures"[ti] OR "Hip Fractures"[ti] OR "Femoral Neck Fractures"[ti] OR "Proximal Femoral Fractures"[ti] OR "Hoffa Fracture"[ti] OR "Proximal Femoral Fractures"[ti] OR "Fibula Fractures"[ti] OR "Knee Fractures"[ti] OR "Hoffa Fracture"[ti] OR "Patella Fracture"[ti] OR "Tibial Plateau Fractures"[ti] OR "Tibial Fractures"[ti] OR "Tibial Plateau Fractures"[ti] OR "Ankle Fracture"[ti] OR "Femoral Fracture"[ti] OR "Hip Fracture"[ti] OR "Femoral Neck Fracture"[ti] OR "Proximal Femoral Fracture"[ti] OR "Hoffa Fractures"[ti] OR "Proximal Femoral Fracture"[ti] OR "Fibula Fracture"[ti] OR "Knee Fracture"[ti] OR "Hoffa Fractures"[ti] OR "Patella Fractures"[ti] OR "Tibial Plateau Fracture"[ti] OR "Tibial Fracture"[ti] OR "Tibial Plateau Fracture"[ti] OR (("Lower Extremity"[majr] OR "lower limb"[ti] OR "lower limbs"[ti] OR "lower extremity"[ti] OR "lower extremities"[ti] OR "Ankle"[ti] OR "Foot"[ti] OR "Heel"[ti] OR "Hip"[ti] OR "Knee"[ti] OR "Leg"[ti] OR "Ankles"[ti] OR "Feet"[ti] OR "Heels"[ti] OR "Hips"[ti] OR "Knees"[ti] OR "Legs"[ti]) AND ("Fractures, Bone"[majr] OR "fracture"[ti] OR "fractures"[ti] OR "fractur*"[ti])) OR **"acutely ill"[ti]**) AND ("immobility"[tw] OR "immobile"[tw] OR "immobil*"[tw] OR "temporary"[tw] OR "non weight bearing"[tw] OR "nonweight bearing"[tw] OR "non weight bear*"[tw] OR "nonweight bear*"[tw] OR "physically dependent"[tw] OR "physically disabled"[tw] OR **"physically dependent"[tw] OR "physically disabled"[tw] OR "disuse"[tw] OR "Bed Rest"[Mesh] OR "bedrest"[tw] OR "bed rest"[tw] OR "leg immobilization"[tw] OR "leg immobilisation"[tw] OR "Weightlessness Simulation"[mesh] OR "Weightlessness Countermeasures"[Mesh] OR "deconditioning"[tw] OR "permissive weight-bearing"[tw]**)**) OR** (("Exercise"[majr] OR "Exercise"[ti] OR "Exercises"[ti] OR "Exercising"[ti] OR "Exercis*"[ti] OR "physical activity"[ti] OR "physical activities"[ti] OR "Endurance Training"[ti] OR "Exergaming"[ti] OR "Gymnastics"[ti] OR "Gymnastic"[ti] OR "Interval Training"[ti] OR "Jogging"[ti] OR "Motor Activity"[ti] OR "Movement"[ti] OR "Muscle Stretching "[ti] OR "Physical Conditioning"[ti] OR "Resistance Training"[ti] OR "Running"[ti] OR "Stair Climbing"[ti] OR "Swimming"[ti] OR "Training"[ti] OR "Walking"[ti] OR "Exercise Therapy"[majr] OR "Physical Exertion"[majr] OR "Physical Exertion"[ti] OR "Physical Exertions"[ti] OR "Physical Effort"[ti] OR "Physical Efforts"[ti] OR "Sports"[majr] OR "Sports"[ti] OR "Sport"[ti] OR "Athletic Performance"[ti] OR "Baseball"[ti] OR "Basketball"[ti] OR "Bicycling"[ti] OR "Boxing"[ti] OR "Cardiorespiratory Fitness"[ti] OR "Cardiorespiratory Fitness"[ti] OR "Cricket Sport"[ti] OR "Diving"[ti] OR "Football"[ti] OR "Golf"[ti] OR "Gymnastics"[ti] OR "Hockey"[ti] OR "Jogging"[ti] OR "Marathon Running"[ti] OR "Martial Arts"[ti] OR "Mountaineering"[ti] OR "Nordic Walking"[ti] OR "Physical Endurance"[ti] OR "Physical Fitness"[ti] OR "Racquet Sports"[ti] OR "Return to Sport"[ti] OR "Rugby"[ti] OR "Running"[ti] OR "Skating"[ti] OR "Skiing"[ti] OR "Snow Sports"[ti] OR "Soccer"[ti] OR "Swimming"[ti] OR "Tai Ji"[ti] OR "Team Sports"[ti] OR "Tennis"[ti] OR "Track and Field"[ti] OR "Volleyball"[ti] OR "Walking"[ti] OR "Water Sports"[ti] OR "Weight Lifting"[ti] OR "Wrestling"[ti] OR "Youth Sports"[ti] OR "Exercise Movement Techniques"[majr] OR "Qigong"[ti] OR "Dance Therapy"[ti] OR "Tai Ji"[ti] OR "Yoga"[ti] OR "Physical Therapy Modalities"[majr:noexp] OR "Physical Therapy"[ti] OR "physiotherapy"[ti] OR "physiotherapy*"[ti] OR "Dry Needling"[majr] OR "Electric Stimulation Therapy"[majr] OR "Extracorporeal Shockwave Therapy"[majr] OR "Hydrotherapy"[majr] OR "Musculoskeletal Manipulations"[majr] OR "Dry Needling"[ti] OR "Electric Stimulation Therapy"[ti] OR "Electroacupuncture"[ti] OR "Pulsed Radiofrequency Treatment"[ti] OR "Spinal Cord Stimulation"[ti] OR "Transcutaneous Electric Nerve Stimulation"[ti] OR "Extracorporeal Shockwave Therapy"[ti] OR "Hydrotherapy"[ti] OR "Aquatic Therapy"[ti] OR "Therapeutic Irrigation"[ti] OR "Musculoskeletal Manipulations"[ti] OR "Applied Kinesiology"[ti] OR "Orthopedic Manipulation"[ti] OR "Orthopaedic Manipulation"[ti] OR "Osteopathic Manipulation"[ti] OR "Spinal Manipulation"[ti] OR "Continuous Passive Motion Therapy"[ti] OR "Soft Tissue Therapy"[ti] OR "Acupressure"[ti] OR "Massage"[ti] OR "Manual Lymphatic Drainage"[ti] OR "Myofascial Release Therapy"[ti] OR "mobilization"[ti] OR "mobilisation"[ti] OR "mobilizing"[ti] OR "mobilising"[ti] OR "mobilize"[ti] OR "mobilise"[ti] OR "mobilized"[ti] OR "mobilised"[ti] OR "mobility"[ti]) AND (**"disuse"[tw]**) AND **("Aged"[mesh] OR "elderly"[all fields] OR "elder"[all fields] OR "elders"[all fields] OR "geriatr*"[all fields] OR "Homes for the Aged"[mesh] OR "Health Services for the Aged"[mesh] OR "Senior Centers"[mesh] OR "Nonagenarians"[all fields] OR "Nonagenarian"[all fields] OR "Octogenarians"[all fields] OR "Octogenarian"[all fields] OR "Centenarians"[all fields] OR "Centenarian"[all fields] OR "septuagenarian"[all fields] OR "septuagenarians"[all fields] OR "Aging"[mesh] OR "aging"[all fields] OR "ageing"[all fields] OR "geront*"[all fields] OR "older person"[all fields] OR "old person"[all fields] OR "older patient"[all fields] OR "old patient"[all fields] OR "older persons"[all fields] OR "old persons"[all fields] OR "older patients"[all fields] OR "old patients"[all fields] OR "older women"[all fields] OR "old women"[all fields] OR "older men"[all fields] OR "old men"[all fields] OR "old adult"[all fields] OR "older adult"[all fields] OR "old adults"[all fields] OR "older adults"[all fields] OR "Older individual"[all fields] OR "Older individuals"[all fields] OR "old people"[all fields] OR "older people"[all fields] OR "Oldest Old"[all fields] OR "older population"[all fields] OR "aging population"[all fields] OR "aging population"[all fields] OR "old aged"[all fields] OR "old age"[all fields] OR "older person"[title/abstract:~3] OR "old person"[title/abstract:~3] OR "older patient"[title/abstract:~3] OR "old patient"[title/abstract:~3] OR "older persons"[title/abstract:~3] OR "old persons"[title/abstract:~3] OR "older patients"[title/abstract:~3] OR "old patients"[title/abstract:~3] OR "older women"[title/abstract:~3] OR "old women"[title/abstract:~3] OR "older men"[title/abstract:~3] OR "old men"[title/abstract:~3] OR "old adult"[title/abstract:~3] OR "older adult"[title/abstract:~3] OR "old adults"[title/abstract:~3] OR "older adults"[title/abstract:~3] OR "Older individual"[title/abstract:~3] OR "Older individuals"[title/abstract:~3] OR "old people"[title/abstract:~3] OR "older people"[title/abstract:~3] OR "Oldest Old"[title/abstract:~3] OR "older population"[title/abstract:~3] OR "aging population"[title/abstract:~3] OR "aging population"[title/abstract:~3] OR "old aged"[title/abstract:~3] OR "old age"[title/abstract:~3] OR** "60 year old"[title/abstract:~3] OR "61 year old"[title/abstract:~3] OR "62 year old"[title/abstract:~3] OR "63 year old"[title/abstract:~3] OR "64 year old"[title/abstract:~3] OR "65 year old"[title/abstract:~3] OR "66 year old"[title/abstract:~3] OR "67 year old"[title/abstract:~3] OR "68 year old"[title/abstract:~3] OR "69 year old"[title/abstract:~3] OR "60 years old"[title/abstract:~3] OR "61 years old"[title/abstract:~3] OR "62 years old"[title/abstract:~3] OR "63 years old"[title/abstract:~3] OR "64 years old"[title/abstract:~3] OR "65 years old"[title/abstract:~3] OR "66 years old"[title/abstract:~3] OR "67 years old"[title/abstract:~3] OR "68 years old"[title/abstract:~3] OR "69 years old"[title/abstract:~3] OR "70 year old"[title/abstract:~3] OR "71 year old"[title/abstract:~3] OR "72 year old"[title/abstract:~3] OR "73 year old"[title/abstract:~3] OR "74 year old"[title/abstract:~3] OR "75 year old"[title/abstract:~3] OR "76 year old"[title/abstract:~3] OR "77 year old"[title/abstract:~3] OR "78 year old"[title/abstract:~3] OR "79 year old"[title/abstract:~3] OR "70 years old"[title/abstract:~3] OR "71 years old"[title/abstract:~3] OR "72 years old"[title/abstract:~3] OR "73 years old"[title/abstract:~3] OR "74 years old"[title/abstract:~3] OR "75 years old"[title/abstract:~3] OR "76 years old"[title/abstract:~3] OR "77 years old"[title/abstract:~3] OR "78 years old"[title/abstract:~3] OR "79 years old"[title/abstract:~3] OR "80 year old"[title/abstract:~3] OR "81 year old"[title/abstract:~3] OR "82 year old"[title/abstract:~3] OR "83 year old"[title/abstract:~3] OR "84 year old"[title/abstract:~3] OR "85 year old"[title/abstract:~3] OR "86 year old"[title/abstract:~3] OR "87 year old"[title/abstract:~3] OR "88 year old"[title/abstract:~3] OR "89 year old"[title/abstract:~3] OR "80 years old"[title/abstract:~3] OR "81 years old"[title/abstract:~3] OR "82 years old"[title/abstract:~3] OR "83 years old"[title/abstract:~3] OR "84 years old"[title/abstract:~3] OR "85 years old"[title/abstract:~3] OR "86 years old"[title/abstract:~3] OR "87 years old"[title/abstract:~3] OR "88 years old"[title/abstract:~3] OR "89 years old"[title/abstract:~3] OR "90 year old"[title/abstract:~3] OR "91 year old"[title/abstract:~3] OR "92 year old"[title/abstract:~3] OR "93 year old"[title/abstract:~3] OR "94 year old"[title/abstract:~3] OR "95 year old"[title/abstract:~3] OR "96 year old"[title/abstract:~3] OR "97 year old"[title/abstract:~3] OR "98 year old"[title/abstract:~3] OR "99 year old"[title/abstract:~3] OR "90 years old"[title/abstract:~3] OR "91 years old"[title/abstract:~3] OR "92 years old"[title/abstract:~3] OR "93 years old"[title/abstract:~3] OR "94 years old"[title/abstract:~3] OR "95 years old"[title/abstract:~3] OR "96 years old"[title/abstract:~3] OR "97 years old"[title/abstract:~3] OR "98 years old"[title/abstract:~3] OR "99 years old"[title/abstract:~3] OR "100 year old"[title/abstract:~3] OR "101 year old"[title/abstract:~3] OR "102 year old"[title/abstract:~3] OR "103 year old"[title/abstract:~3] OR "104 year old"[title/abstract:~3] OR "105 year old"[title/abstract:~3] OR "106 year old"[title/abstract:~3] OR "107 year old"[title/abstract:~3] OR "108 year old"[title/abstract:~3] OR "109 year old"[title/abstract:~3] OR "100 years old"[title/abstract:~3] OR "101 years old"[title/abstract:~3] OR "102 years old"[title/abstract:~3] OR "103 years old"[title/abstract:~3] OR "104 years old"[title/abstract:~3] OR "105 years old"[title/abstract:~3] OR "106 years old"[title/abstract:~3] OR "107 years old"[title/abstract:~3] OR "108 years old"[title/abstract:~3] OR "109 years old"[title/abstract:~3]**))) NOT ("Animals"[mesh] NOT "Humans"[mesh])** NOT (("Case Reports"[ptyp] OR "case report"[ti] OR "case rep"[all fields] OR "Review"[ptyp] OR "review"[ti]) NOT ("Clinical Study"[ptyp] OR "trial"[ti] OR "RCT"[ti] OR "systematic"[sb])) AND english[la]

**Embase**

**(**((exp *"Wheelchair"/ OR "Wheelchairs".ti,ab OR "Wheelchair".ti,ab OR "Wheelchair*".ti,ab OR "Wheel chairs".ti,ab OR "Wheel chair".ti,ab OR "Wheel chair*".ti,ab OR "Mobility Scooter".ti,ab OR "Mobility Scooters".ti,ab) AND (exp *"Physical Activity"/ OR exp *"Exercise"/ OR "Exercise".ti,ab OR "Exercises".ti,ab OR "Exercising".ti,ab OR "Exercis*".ti,ab OR "physical activity".ti,ab OR "physical activities".ti,ab OR "Endurance Training".ti,ab OR "Exergaming".ti,ab OR "Gymnastics".ti,ab OR "Gymnastic".ti,ab OR "Interval Training".ti,ab OR "Jogging".ti,ab OR "Motor Activity".ti,ab OR "Movement".ti,ab OR "Muscle Stretching ".ti,ab OR "Physical Conditioning".ti,ab OR "Resistance Training".ti,ab OR "Running".ti,ab OR "Stair Climbing".ti,ab OR "Swimming".ti,ab OR "Training".ti,ab OR "Walking".ti,ab OR exp *"Kinesiotherapy"/ OR "Physical Exertion".ti,ab OR "Physical Exertions".ti,ab OR "Physical Effort".ti,ab OR "Physical Efforts".ti,ab OR exp *"Sport"/ OR "Sports".ti,ab OR "Sport".ti,ab OR "Athletic Performance".ti,ab OR "Baseball".ti,ab OR "Basketball".ti,ab OR "Bicycling".ti,ab OR "Boxing".ti,ab OR "Cardiorespiratory Fitness".ti,ab OR "Cardiorespiratory Fitness".ti,ab OR "Cricket Sport".ti,ab OR "Diving".ti,ab OR "Football".ti,ab OR "Golf".ti,ab OR "Gymnastics".ti,ab OR "Hockey".ti,ab OR "Jogging".ti,ab OR "Marathon Running".ti,ab OR "Martial Arts".ti,ab OR "Mountaineering".ti,ab OR "Nordic Walking".ti,ab OR "Physical Endurance".ti,ab OR "Physical Fitness".ti,ab OR "Racquet Sports".ti,ab OR "Return to Sport".ti,ab OR "Rugby".ti,ab OR "Running".ti,ab OR "Skating".ti,ab OR "Skiing".ti,ab OR "Snow Sports".ti,ab OR "Soccer".ti,ab OR "Swimming".ti,ab OR "Tai Ji".ti,ab OR "Team Sports".ti,ab OR "Tennis".ti,ab OR "Track and Field".ti,ab OR "Volleyball".ti,ab OR "Walking".ti,ab OR "Water Sports".ti,ab OR "Weight Lifting".ti,ab OR "Wrestling".ti,ab OR "Youth Sports".ti,ab OR "Qigong".ti,ab OR "Dance Therapy".ti,ab OR "Tai Ji".ti,ab OR "Yoga".ti,ab OR exp *"Physiotherapy"/ OR "Physical Therapy".ti,ab OR "physiotherapy".ti,ab OR "physiotherapy*".ti,ab OR exp *"Dry Needling"/ OR exp *"Electrotherapy"/ OR exp *"Shock wave Therapy"/ OR exp *"Balneotherapy"/ OR exp *"Musculoskeletal Manipulation"/ OR "Dry Needling".ti,ab OR "Electric Stimulation Therapy".ti,ab OR "Electroacupuncture".ti,ab OR "Pulsed Radiofrequency Treatment".ti,ab OR "Spinal Cord Stimulation".ti,ab OR "Transcutaneous Electric Nerve Stimulation".ti,ab OR "Extracorporeal Shockwave Therapy".ti,ab OR "Hydrotherapy".ti,ab OR "Aquatic Therapy".ti,ab OR "Therapeutic Irrigation".ti,ab OR "Musculoskeletal Manipulations".ti,ab OR "Applied Kinesiology".ti,ab OR "Orthopedic Manipulation".ti,ab OR "Orthopaedic Manipulation".ti,ab OR "Osteopathic Manipulation".ti,ab OR "Spinal Manipulation".ti,ab OR "Continuous Passive Motion Therapy".ti,ab OR "Soft Tissue Therapy".ti,ab OR "Acupressure".ti,ab OR "Massage".ti,ab OR "Manual Lymphatic Drainage".ti,ab OR "Myofascial Release Therapy".ti,ab OR exp *"mobilization"/ OR "mobilization".ti,ab OR "mobilisation".ti,ab OR "mobilizing".ti,ab OR "mobilising".ti,ab OR "mobilize".ti,ab OR "mobilise".ti,ab OR "mobilized".ti,ab OR "mobilised".ti,ab OR "mobility".ti,ab) AND (exp *"Ankle Fracture"/ OR exp *"Femur Fracture"/ OR exp *"Fibula Fracture"/ OR exp *"Knee Fracture"/ OR exp *"Tibia Fracture"/ OR "Ankle Fractures".ti,ab OR "Femoral Fractures".ti,ab OR "Hip Fractures".ti,ab OR "Femoral Neck Fractures".ti,ab OR "Proximal Femoral Fractures".ti,ab OR "Hoffa Fracture".ti,ab OR "Proximal Femoral Fractures".ti,ab OR "Fibula Fractures".ti,ab OR "Knee Fractures".ti,ab OR "Hoffa Fracture".ti,ab OR "Patella Fracture".ti,ab OR "Tibial Plateau Fractures".ti,ab OR "Tibial Fractures".ti,ab OR "Tibial Plateau Fractures".ti,ab OR "Ankle Fracture".ti,ab OR "Femoral Fracture".ti,ab OR "Hip Fracture".ti,ab OR "Femoral Neck Fracture".ti,ab OR "Proximal Femoral Fracture".ti,ab OR "Hoffa Fractures".ti,ab OR "Proximal Femoral Fracture".ti,ab OR "Fibula Fracture".ti,ab OR "Knee Fracture".ti,ab OR "Hoffa Fractures".ti,ab OR "Patella Fractures".ti,ab OR "Tibial Plateau Fracture".ti,ab OR "Tibial Fracture".ti,ab OR "Tibial Plateau Fracture".ti,ab OR ((exp *"Lower Limb"/ OR "lower limb".ti,ab OR "lower limbs".ti,ab OR "lower extremity".ti,ab OR "lower extremities".ti,ab OR "Ankle".ti,ab OR "Foot".ti,ab OR "Heel".ti,ab OR "Hip".ti,ab OR "Knee".ti,ab OR "Leg".ti,ab OR "Ankles".ti,ab OR "Feet".ti,ab OR "Heels".ti,ab OR "Hips".ti,ab OR "Knees".ti,ab OR "Legs".ti,ab) AND (exp *"Fracture"/ OR "fracture".ti,ab OR "fractures".ti,ab OR "fractur*".ti,ab)) OR exp *"immobility"/ OR "immobility".ti,ab OR "immobile".ti,ab OR "immobil*".ti,ab OR "temporary".ti,ab OR exp *"non weight bearing"/ OR "non weight bearing".ti,ab OR "nonweight bearing".ti,ab OR "non weight bear*".ti,ab OR "nonweight bear*".ti,ab)**) OR** ((exp *"Wheelchair"/ OR "Wheelchairs".ti OR "Wheelchair".ti OR "Wheelchair*".ti OR "Wheel chairs".ti OR "Wheel chair".ti OR "Wheel chair*".ti OR "Mobility Scooter".ti OR "Mobility Scooters".ti) AND (exp *"Physical Activity"/ OR exp *"Exercise"/ OR "Exercise".ti OR "Exercises".ti OR "Exercising".ti OR "Exercis*".ti OR "physical activity".ti OR "physical activities".ti OR "Endurance Training".ti OR "Exergaming".ti OR "Gymnastics".ti OR "Gymnastic".ti OR "Interval Training".ti OR "Jogging".ti OR "Motor Activity".ti OR "Movement".ti OR "Muscle Stretching ".ti OR "Physical Conditioning".ti OR "Resistance Training".ti OR "Running".ti OR "Stair Climbing".ti OR "Swimming".ti OR "Training".ti OR "Walking".ti OR exp *"Kinesiotherapy"/ OR "Physical Exertion".ti OR "Physical Exertions".ti OR "Physical Effort".ti OR "Physical Efforts".ti OR exp *"Sport"/ OR "Sports".ti OR "Sport".ti OR "Athletic Performance".ti OR "Baseball".ti OR "Basketball".ti OR "Bicycling".ti OR "Boxing".ti OR "Cardiorespiratory Fitness".ti OR "Cardiorespiratory Fitness".ti OR "Cricket Sport".ti OR "Diving".ti OR "Football".ti OR "Golf".ti OR "Gymnastics".ti OR "Hockey".ti OR "Jogging".ti OR "Marathon Running".ti OR "Martial Arts".ti OR "Mountaineering".ti OR "Nordic Walking".ti OR "Physical Endurance".ti OR "Physical Fitness".ti OR "Racquet Sports".ti OR "Return to Sport".ti OR "Rugby".ti OR "Running".ti OR "Skating".ti OR "Skiing".ti OR "Snow Sports".ti OR "Soccer".ti OR "Swimming".ti OR "Tai Ji".ti OR "Team Sports".ti OR "Tennis".ti OR "Track and Field".ti OR "Volleyball".ti OR "Walking".ti OR "Water Sports".ti OR "Weight Lifting".ti OR "Wrestling".ti OR "Youth Sports".ti OR "Qigong".ti OR "Dance Therapy".ti OR "Tai Ji".ti OR "Yoga".ti OR exp *"Physiotherapy"/ OR "Physical Therapy".ti OR "physiotherapy".ti OR "physiotherapy*".ti OR exp *"Dry Needling"/ OR exp *"Electrotherapy"/ OR exp *"Shock wave Therapy"/ OR exp *"Balneotherapy"/ OR exp *"Musculoskeletal Manipulation"/ OR "Dry Needling".ti OR "Electric Stimulation Therapy".ti OR "Electroacupuncture".ti OR "Pulsed Radiofrequency Treatment".ti OR "Spinal Cord Stimulation".ti OR "Transcutaneous Electric Nerve Stimulation".ti OR "Extracorporeal Shockwave Therapy".ti OR "Hydrotherapy".ti OR "Aquatic Therapy".ti OR "Therapeutic Irrigation".ti OR "Musculoskeletal Manipulations".ti OR "Applied Kinesiology".ti OR "Orthopedic Manipulation".ti OR "Orthopaedic Manipulation".ti OR "Osteopathic Manipulation".ti OR "Spinal Manipulation".ti OR "Continuous Passive Motion Therapy".ti OR "Soft Tissue Therapy".ti OR "Acupressure".ti OR "Massage".ti OR "Manual Lymphatic Drainage".ti OR "Myofascial Release Therapy".ti OR exp *"mobilization"/ OR "mobilization".ti OR "mobilisation".ti OR "mobilizing".ti OR "mobilising".ti OR "mobilize".ti OR "mobilise".ti OR "mobilized".ti OR "mobilised".ti OR "mobility".ti) AND **(exp "Aged"/ OR "elderly".af OR "elder".af OR "elders".af OR "geriatr*".af OR exp "Home for the Aged"/ OR exp "Elderly care"/ OR exp "Senior Center"/ OR "Nonagenarians".af OR "Nonagenarian".af OR "Octogenarians".af OR "Octogenarian".af OR "Centenarians".af OR "Centenarian".af OR "septuagenarian".af OR "septuagenarians".af OR "geront*".af OR "older person".af OR "old person".af OR "older patient".af OR "old patient".af OR "older persons".af OR "old persons".af OR "older patients".af OR "old patients".af OR "older women".af OR "old women".af OR "older men".af OR "old men".af OR "old adult".af OR "older adult".af OR "old adults".af OR "older adults".af OR "Older individual".af OR "Older individuals".af OR "old people".af OR "older people".af OR "Oldest Old".af OR "older population".af OR "aging population".af OR "aging population".af OR "old aged".af OR "old age".af OR (("older" ADJ3 "person") OR ("old" ADJ3 "person") OR ("older" ADJ3 "patient") OR ("old" ADJ3 "patient") OR ("older" ADJ3 "persons") OR ("old" ADJ3 "persons") OR ("older" ADJ3 "patients") OR ("old" ADJ3 "patients") OR ("older" ADJ3 "women") OR ("old" ADJ3 "women") OR ("older" ADJ3 "men") OR ("old" ADJ3 "men") OR ("old" ADJ3 "adult") OR ("older" ADJ3 "adult") OR ("old" ADJ3 "adults") OR ("older" ADJ3 "adults") OR ("Older" ADJ3 "individual") OR ("Older" ADJ3 "individuals") OR ("old" ADJ3 "people") OR ("older" ADJ3 "people") OR ("Oldest" ADJ3 "Old") OR ("older" ADJ3 "population") OR ("aging" ADJ3 "population") OR ("aging" ADJ3 "population") OR ("old" ADJ3 "aged") OR ("old" ADJ3 "age") OR (**"60" ADJ3 "year" ADJ3 "old") OR ("61" ADJ3 "year" ADJ3 "old") OR ("62" ADJ3 "year" ADJ3 "old") OR ("63" ADJ3 "year" ADJ3 "old") OR ("64" ADJ3 "year" ADJ3 "old") OR ("65" ADJ3 "year" ADJ3 "old") OR ("66" ADJ3 "year" ADJ3 "old") OR ("67" ADJ3 "year" ADJ3 "old") OR ("68" ADJ3 "year" ADJ3 "old") OR ("69" ADJ3 "year" ADJ3 "old") OR ("60" ADJ3 "years" ADJ3 "old") OR ("61" ADJ3 "years" ADJ3 "old") OR ("62" ADJ3 "years" ADJ3 "old") OR ("63" ADJ3 "years" ADJ3 "old") OR ("64" ADJ3 "years" ADJ3 "old") OR ("65" ADJ3 "years" ADJ3 "old") OR ("66" ADJ3 "years" ADJ3 "old") OR ("67" ADJ3 "years" ADJ3 "old") OR ("68" ADJ3 "years" ADJ3 "old") OR ("69" ADJ3 "years" ADJ3 "old") OR ("70" ADJ3 "year" ADJ3 "old") OR ("71" ADJ3 "year" ADJ3 "old") OR ("72" ADJ3 "year" ADJ3 "old") OR ("73" ADJ3 "year" ADJ3 "old") OR ("74" ADJ3 "year" ADJ3 "old") OR ("75" ADJ3 "year" ADJ3 "old") OR ("76" ADJ3 "year" ADJ3 "old") OR ("77" ADJ3 "year" ADJ3 "old") OR ("78" ADJ3 "year" ADJ3 "old") OR ("79" ADJ3 "year" ADJ3 "old") OR ("70" ADJ3 "years" ADJ3 "old") OR ("71" ADJ3 "years" ADJ3 "old") OR ("72" ADJ3 "years" ADJ3 "old") OR ("73" ADJ3 "years" ADJ3 "old") OR ("74" ADJ3 "years" ADJ3 "old") OR ("75" ADJ3 "years" ADJ3 "old") OR ("76" ADJ3 "years" ADJ3 "old") OR ("77" ADJ3 "years" ADJ3 "old") OR ("78" ADJ3 "years" ADJ3 "old") OR ("79" ADJ3 "years" ADJ3 "old") OR ("80" ADJ3 "year" ADJ3 "old") OR ("81" ADJ3 "year" ADJ3 "old") OR ("82" ADJ3 "year" ADJ3 "old") OR ("83" ADJ3 "year" ADJ3 "old") OR ("84" ADJ3 "year" ADJ3 "old") OR ("85" ADJ3 "year" ADJ3 "old") OR ("86" ADJ3 "year" ADJ3 "old") OR ("87" ADJ3 "year" ADJ3 "old") OR ("88" ADJ3 "year" ADJ3 "old") OR ("89" ADJ3 "year" ADJ3 "old") OR ("80" ADJ3 "years" ADJ3 "old") OR ("81" ADJ3 "years" ADJ3 "old") OR ("82" ADJ3 "years" ADJ3 "old") OR ("83" ADJ3 "years" ADJ3 "old") OR ("84" ADJ3 "years" ADJ3 "old") OR ("85" ADJ3 "years" ADJ3 "old") OR ("86" ADJ3 "years" ADJ3 "old") OR ("87" ADJ3 "years" ADJ3 "old") OR ("88" ADJ3 "years" ADJ3 "old") OR ("89" ADJ3 "years" ADJ3 "old") OR ("90" ADJ3 "year" ADJ3 "old") OR ("91" ADJ3 "year" ADJ3 "old") OR ("92" ADJ3 "year" ADJ3 "old") OR ("93" ADJ3 "year" ADJ3 "old") OR ("94" ADJ3 "year" ADJ3 "old") OR ("95" ADJ3 "year" ADJ3 "old") OR ("96" ADJ3 "year" ADJ3 "old") OR ("97" ADJ3 "year" ADJ3 "old") OR ("98" ADJ3 "year" ADJ3 "old") OR ("99" ADJ3 "year" ADJ3 "old") OR ("90" ADJ3 "years" ADJ3 "old") OR ("91" ADJ3 "years" ADJ3 "old") OR ("92" ADJ3 "years" ADJ3 "old") OR ("93" ADJ3 "years" ADJ3 "old") OR ("94" ADJ3 "years" ADJ3 "old") OR ("95" ADJ3 "years" ADJ3 "old") OR ("96" ADJ3 "years" ADJ3 "old") OR ("97" ADJ3 "years" ADJ3 "old") OR ("98" ADJ3 "years" ADJ3 "old") OR ("99" ADJ3 "years" ADJ3 "old") OR ("100" ADJ3 "year" ADJ3 "old") OR ("101" ADJ3 "year" ADJ3 "old") OR ("102" ADJ3 "year" ADJ3 "old") OR ("103" ADJ3 "year" ADJ3 "old") OR ("104" ADJ3 "year" ADJ3 "old") OR ("105" ADJ3 "year" ADJ3 "old") OR ("106" ADJ3 "year" ADJ3 "old") OR ("107" ADJ3 "year" ADJ3 "old") OR ("108" ADJ3 "year" ADJ3 "old") OR ("109" ADJ3 "year" ADJ3 "old") OR ("100" ADJ3 "years" ADJ3 "old") OR ("101" ADJ3 "years" ADJ3 "old") OR ("102" ADJ3 "years" ADJ3 "old") OR ("103" ADJ3 "years" ADJ3 "old") OR ("104" ADJ3 "years" ADJ3 "old") OR ("105" ADJ3 "years" ADJ3 "old") OR ("106" ADJ3 "years" ADJ3 "old") OR ("107" ADJ3 "years" ADJ3 "old") OR ("108" ADJ3 "years" ADJ3 "old") OR ("109" ADJ3 "years" ADJ3 "old"**)).ti,ab)) OR** ((exp *"Physical Activity"/ OR exp *"Exercise"/ OR "Exercise".ti,ab OR "Exercises".ti,ab OR "Exercising".ti,ab OR "Exercis*".ti,ab OR "physical activity".ti,ab OR "physical activities".ti,ab OR "Endurance Training".ti,ab OR "Exergaming".ti,ab OR "Gymnastics".ti,ab OR "Gymnastic".ti,ab OR "Interval Training".ti,ab OR "Jogging".ti,ab OR "Motor Activity".ti,ab OR "Movement".ti,ab OR "Muscle Stretching ".ti,ab OR "Physical Conditioning".ti,ab OR "Resistance Training".ti,ab OR "Running".ti,ab OR "Stair Climbing".ti,ab OR "Swimming".ti,ab OR "Training".ti,ab OR "Walking".ti,ab OR exp *"Kinesiotherapy"/ OR "Physical Exertion".ti,ab OR "Physical Exertions".ti,ab OR "Physical Effort".ti,ab OR "Physical Efforts".ti,ab OR exp *"Sport"/ OR "Sports".ti,ab OR "Sport".ti,ab OR "Athletic Performance".ti,ab OR "Baseball".ti,ab OR "Basketball".ti,ab OR "Bicycling".ti,ab OR "Boxing".ti,ab OR "Cardiorespiratory Fitness".ti,ab OR "Cardiorespiratory Fitness".ti,ab OR "Cricket Sport".ti,ab OR "Diving".ti,ab OR "Football".ti,ab OR "Golf".ti,ab OR "Gymnastics".ti,ab OR "Hockey".ti,ab OR "Jogging".ti,ab OR "Marathon Running".ti,ab OR "Martial Arts".ti,ab OR "Mountaineering".ti,ab OR "Nordic Walking".ti,ab OR "Physical Endurance".ti,ab OR "Physical Fitness".ti,ab OR "Racquet Sports".ti,ab OR "Return to Sport".ti,ab OR "Rugby".ti,ab OR "Running".ti,ab OR "Skating".ti,ab OR "Skiing".ti,ab OR "Snow Sports".ti,ab OR "Soccer".ti,ab OR "Swimming".ti,ab OR "Tai Ji".ti,ab OR "Team Sports".ti,ab OR "Tennis".ti,ab OR "Track and Field".ti,ab OR "Volleyball".ti,ab OR "Walking".ti,ab OR "Water Sports".ti,ab OR "Weight Lifting".ti,ab OR "Wrestling".ti,ab OR "Youth Sports".ti,ab OR "Qigong".ti,ab OR "Dance Therapy".ti,ab OR "Tai Ji".ti,ab OR "Yoga".ti,ab OR exp *"Physiotherapy"/ OR "Physical Therapy".ti,ab OR "physiotherapy".ti,ab OR "physiotherapy*".ti,ab OR exp *"Dry Needling"/ OR exp *"Electrotherapy"/ OR exp *"Shock wave Therapy"/ OR exp *"Balneotherapy"/ OR exp *"Musculoskeletal Manipulation"/ OR "Dry Needling".ti,ab OR "Electric Stimulation Therapy".ti,ab OR "Electroacupuncture".ti,ab OR "Pulsed Radiofrequency Treatment".ti,ab OR "Spinal Cord Stimulation".ti,ab OR "Transcutaneous Electric Nerve Stimulation".ti,ab OR "Extracorporeal Shockwave Therapy".ti,ab OR "Hydrotherapy".ti,ab OR "Aquatic Therapy".ti,ab OR "Therapeutic Irrigation".ti,ab OR "Musculoskeletal Manipulations".ti,ab OR "Applied Kinesiology".ti,ab OR "Orthopedic Manipulation".ti,ab OR "Orthopaedic Manipulation".ti,ab OR "Osteopathic Manipulation".ti,ab OR "Spinal Manipulation".ti,ab OR "Continuous Passive Motion Therapy".ti,ab OR "Soft Tissue Therapy".ti,ab OR "Acupressure".ti,ab OR "Massage".ti,ab OR "Manual Lymphatic Drainage".ti,ab OR "Myofascial Release Therapy".ti,ab OR exp *"mobilization"/ OR "mobilization".ti,ab OR "mobilisation".ti,ab OR "mobilizing".ti,ab OR "mobilising".ti,ab OR "mobilize".ti,ab OR "mobilise".ti,ab OR "mobilized".ti,ab OR "mobilised".ti,ab OR "mobility".ti,ab) AND (exp *"Ankle Fracture"/ OR exp *"Femur Fracture"/ OR exp *"Fibula Fracture"/ OR exp *"Knee Fracture"/ OR exp *"Tibia Fracture"/ OR "Ankle Fractures".ti OR "Femoral Fractures".ti OR "Hip Fractures".ti OR "Femoral Neck Fractures".ti OR "Proximal Femoral Fractures".ti OR "Hoffa Fracture".ti OR "Proximal Femoral Fractures".ti OR "Fibula Fractures".ti OR "Knee Fractures".ti OR "Hoffa Fracture".ti OR "Patella Fracture".ti OR "Tibial Plateau Fractures".ti OR "Tibial Fractures".ti OR "Tibial Plateau Fractures".ti OR "Ankle Fracture".ti OR "Femoral Fracture".ti OR "Hip Fracture".ti OR "Femoral Neck Fracture".ti OR "Proximal Femoral Fracture".ti OR "Hoffa Fractures".ti OR "Proximal Femoral Fracture".ti OR "Fibula Fracture".ti OR "Knee Fracture".ti OR "Hoffa Fractures".ti OR "Patella Fractures".ti OR "Tibial Plateau Fracture".ti OR "Tibial Fracture".ti OR "Tibial Plateau Fracture".ti OR ((exp *"Lower Limb"/ OR "lower limb".ti OR "lower limbs".ti OR "lower extremity".ti OR "lower extremities".ti OR "Ankle".ti OR "Foot".ti OR "Heel".ti OR "Hip".ti OR "Knee".ti OR "Leg".ti OR "Ankles".ti OR "Feet".ti OR "Heels".ti OR "Hips".ti OR "Knees".ti OR "Legs".ti) AND (exp *"Fracture"/ OR "fracture".ti OR "fractures".ti OR "fractur*".ti)) OR **"acutely ill".ti**) AND (exp *"immobility"/ OR "immobility".ti,ab OR "immobile".ti,ab OR "immobil*".ti,ab OR "temporary".ti,ab OR exp *"non weight bearing"/ OR "non weight bearing".ti,ab OR "nonweight bearing".ti,ab OR "non weight bear*".ti,ab OR "nonweight bear*".ti,ab OR "physically dependent".ti,ab OR "physically disabled".ti,ab OR **"physically dependent".ti,ab OR "physically disabled".ti,ab OR "disuse".ti,ab OR exp *"bed rest"/ OR "bed rest".ti,ab OR "bedrest".ti,ab OR "leg immobilization".ti,ab OR "leg immobilisation".ti,ab OR exp *"Weightlessness"/ OR exp *"deconditioning"/ OR "deconditioning".ti,ab OR "permissive weight-bearing".ti,ab**)**) OR** ((exp *"Physical Activity"/ OR exp *"Exercise"/ OR "Exercise".ti OR "Exercises".ti OR "Exercising".ti OR "Exercis*".ti OR "physical activity".ti OR "physical activities".ti OR "Endurance Training".ti OR "Exergaming".ti OR "Gymnastics".ti OR "Gymnastic".ti OR "Interval Training".ti OR "Jogging".ti OR "Motor Activity".ti OR "Movement".ti OR "Muscle Stretching ".ti OR "Physical Conditioning".ti OR "Resistance Training".ti OR "Running".ti OR "Stair Climbing".ti OR "Swimming".ti OR "Training".ti OR "Walking".ti OR exp *"Kinesiotherapy"/ OR "Physical Exertion".ti OR "Physical Exertions".ti OR "Physical Effort".ti OR "Physical Efforts".ti OR exp *"Sport"/ OR "Sports".ti OR "Sport".ti OR "Athletic Performance".ti OR "Baseball".ti OR "Basketball".ti OR "Bicycling".ti OR "Boxing".ti OR "Cardiorespiratory Fitness".ti OR "Cardiorespiratory Fitness".ti OR "Cricket Sport".ti OR "Diving".ti OR "Football".ti OR "Golf".ti OR "Gymnastics".ti OR "Hockey".ti OR "Jogging".ti OR "Marathon Running".ti OR "Martial Arts".ti OR "Mountaineering".ti OR "Nordic Walking".ti OR "Physical Endurance".ti OR "Physical Fitness".ti OR "Racquet Sports".ti OR "Return to Sport".ti OR "Rugby".ti OR "Running".ti OR "Skating".ti OR "Skiing".ti OR "Snow Sports".ti OR "Soccer".ti OR "Swimming".ti OR "Tai Ji".ti OR "Team Sports".ti OR "Tennis".ti OR "Track and Field".ti OR "Volleyball".ti OR "Walking".ti OR "Water Sports".ti OR "Weight Lifting".ti OR "Wrestling".ti OR "Youth Sports".ti OR "Qigong".ti OR "Dance Therapy".ti OR "Tai Ji".ti OR "Yoga".ti OR exp *"Physiotherapy"/ OR "Physical Therapy".ti OR "physiotherapy".ti OR "physiotherapy*".ti OR exp *"Dry Needling"/ OR exp *"Electrotherapy"/ OR exp *"Shock wave Therapy"/ OR exp *"Balneotherapy"/ OR exp *"Musculoskeletal Manipulation"/ OR "Dry Needling".ti OR "Electric Stimulation Therapy".ti OR "Electroacupuncture".ti OR "Pulsed Radiofrequency Treatment".ti OR "Spinal Cord Stimulation".ti OR "Transcutaneous Electric Nerve Stimulation".ti OR "Extracorporeal Shockwave Therapy".ti OR "Hydrotherapy".ti OR "Aquatic Therapy".ti OR "Therapeutic Irrigation".ti OR "Musculoskeletal Manipulations".ti OR "Applied Kinesiology".ti OR "Orthopedic Manipulation".ti OR "Orthopaedic Manipulation".ti OR "Osteopathic Manipulation".ti OR "Spinal Manipulation".ti OR "Continuous Passive Motion Therapy".ti OR "Soft Tissue Therapy".ti OR "Acupressure".ti OR "Massage".ti OR "Manual Lymphatic Drainage".ti OR "Myofascial Release Therapy".ti OR exp *"mobilization"/ OR "mobilization".ti OR "mobilisation".ti OR "mobilizing".ti OR "mobilising".ti OR "mobilize".ti OR "mobilise".ti OR "mobilized".ti OR "mobilised".ti OR "mobility".ti) AND (**"disuse".ti,ab**) AND **(exp "Aged"/ OR "elderly".af OR "elder".af OR "elders".af OR "geriatr*".af OR exp "Home for the Aged"/ OR exp "Elderly care"/ OR exp "Senior Center"/ OR "Nonagenarians".af OR "Nonagenarian".af OR "Octogenarians".af OR "Octogenarian".af OR "Centenarians".af OR "Centenarian".af OR "septuagenarian".af OR "septuagenarians".af OR "geront*".af OR "older person".af OR "old person".af OR "older patient".af OR "old patient".af OR "older persons".af OR "old persons".af OR "older patients".af OR "old patients".af OR "older women".af OR "old women".af OR "older men".af OR "old men".af OR "old adult".af OR "older adult".af OR "old adults".af OR "older adults".af OR "Older individual".af OR "Older individuals".af OR "old people".af OR "older people".af OR "Oldest Old".af OR "older population".af OR "aging population".af OR "aging population".af OR "old aged".af OR "old age".af OR (("older" ADJ3 "person") OR ("old" ADJ3 "person") OR ("older" ADJ3 "patient") OR ("old" ADJ3 "patient") OR ("older" ADJ3 "persons") OR ("old" ADJ3 "persons") OR ("older" ADJ3 "patients") OR ("old" ADJ3 "patients") OR ("older" ADJ3 "women") OR ("old" ADJ3 "women") OR ("older" ADJ3 "men") OR ("old" ADJ3 "men") OR ("old" ADJ3 "adult") OR ("older" ADJ3 "adult") OR ("old" ADJ3 "adults") OR ("older" ADJ3 "adults") OR ("Older" ADJ3 "individual") OR ("Older" ADJ3 "individuals") OR ("old" ADJ3 "people") OR ("older" ADJ3 "people") OR ("Oldest" ADJ3 "Old") OR ("older" ADJ3 "population") OR ("aging" ADJ3 "population") OR ("aging" ADJ3 "population") OR ("old" ADJ3 "aged") OR ("old" ADJ3 "age") OR (**"60" ADJ3 "year" ADJ3 "old") OR ("61" ADJ3 "year" ADJ3 "old") OR ("62" ADJ3 "year" ADJ3 "old") OR ("63" ADJ3 "year" ADJ3 "old") OR ("64" ADJ3 "year" ADJ3 "old") OR ("65" ADJ3 "year" ADJ3 "old") OR ("66" ADJ3 "year" ADJ3 "old") OR ("67" ADJ3 "year" ADJ3 "old") OR ("68" ADJ3 "year" ADJ3 "old") OR ("69" ADJ3 "year" ADJ3 "old") OR ("60" ADJ3 "years" ADJ3 "old") OR ("61" ADJ3 "years" ADJ3 "old") OR ("62" ADJ3 "years" ADJ3 "old") OR ("63" ADJ3 "years" ADJ3 "old") OR ("64" ADJ3 "years" ADJ3 "old") OR ("65" ADJ3 "years" ADJ3 "old") OR ("66" ADJ3 "years" ADJ3 "old") OR ("67" ADJ3 "years" ADJ3 "old") OR ("68" ADJ3 "years" ADJ3 "old") OR ("69" ADJ3 "years" ADJ3 "old") OR ("70" ADJ3 "year" ADJ3 "old") OR ("71" ADJ3 "year" ADJ3 "old") OR ("72" ADJ3 "year" ADJ3 "old") OR ("73" ADJ3 "year" ADJ3 "old") OR ("74" ADJ3 "year" ADJ3 "old") OR ("75" ADJ3 "year" ADJ3 "old") OR ("76" ADJ3 "year" ADJ3 "old") OR ("77" ADJ3 "year" ADJ3 "old") OR ("78" ADJ3 "year" ADJ3 "old") OR ("79" ADJ3 "year" ADJ3 "old") OR ("70" ADJ3 "years" ADJ3 "old") OR ("71" ADJ3 "years" ADJ3 "old") OR ("72" ADJ3 "years" ADJ3 "old") OR ("73" ADJ3 "years" ADJ3 "old") OR ("74" ADJ3 "years" ADJ3 "old") OR ("75" ADJ3 "years" ADJ3 "old") OR ("76" ADJ3 "years" ADJ3 "old") OR ("77" ADJ3 "years" ADJ3 "old") OR ("78" ADJ3 "years" ADJ3 "old") OR ("79" ADJ3 "years" ADJ3 "old") OR ("80" ADJ3 "year" ADJ3 "old") OR ("81" ADJ3 "year" ADJ3 "old") OR ("82" ADJ3 "year" ADJ3 "old") OR ("83" ADJ3 "year" ADJ3 "old") OR ("84" ADJ3 "year" ADJ3 "old") OR ("85" ADJ3 "year" ADJ3 "old") OR ("86" ADJ3 "year" ADJ3 "old") OR ("87" ADJ3 "year" ADJ3 "old") OR ("88" ADJ3 "year" ADJ3 "old") OR ("89" ADJ3 "year" ADJ3 "old") OR ("80" ADJ3 "years" ADJ3 "old") OR ("81" ADJ3 "years" ADJ3 "old") OR ("82" ADJ3 "years" ADJ3 "old") OR ("83" ADJ3 "years" ADJ3 "old") OR ("84" ADJ3 "years" ADJ3 "old") OR ("85" ADJ3 "years" ADJ3 "old") OR ("86" ADJ3 "years" ADJ3 "old") OR ("87" ADJ3 "years" ADJ3 "old") OR ("88" ADJ3 "years" ADJ3 "old") OR ("89" ADJ3 "years" ADJ3 "old") OR ("90" ADJ3 "year" ADJ3 "old") OR ("91" ADJ3 "year" ADJ3 "old") OR ("92" ADJ3 "year" ADJ3 "old") OR ("93" ADJ3 "year" ADJ3 "old") OR ("94" ADJ3 "year" ADJ3 "old") OR ("95" ADJ3 "year" ADJ3 "old") OR ("96" ADJ3 "year" ADJ3 "old") OR ("97" ADJ3 "year" ADJ3 "old") OR ("98" ADJ3 "year" ADJ3 "old") OR ("99" ADJ3 "year" ADJ3 "old") OR ("90" ADJ3 "years" ADJ3 "old") OR ("91" ADJ3 "years" ADJ3 "old") OR ("92" ADJ3 "years" ADJ3 "old") OR ("93" ADJ3 "years" ADJ3 "old") OR ("94" ADJ3 "years" ADJ3 "old") OR ("95" ADJ3 "years" ADJ3 "old") OR ("96" ADJ3 "years" ADJ3 "old") OR ("97" ADJ3 "years" ADJ3 "old") OR ("98" ADJ3 "years" ADJ3 "old") OR ("99" ADJ3 "years" ADJ3 "old") OR ("100" ADJ3 "year" ADJ3 "old") OR ("101" ADJ3 "year" ADJ3 "old") OR ("102" ADJ3 "year" ADJ3 "old") OR ("103" ADJ3 "year" ADJ3 "old") OR ("104" ADJ3 "year" ADJ3 "old") OR ("105" ADJ3 "year" ADJ3 "old") OR ("106" ADJ3 "year" ADJ3 "old") OR ("107" ADJ3 "year" ADJ3 "old") OR ("108" ADJ3 "year" ADJ3 "old") OR ("109" ADJ3 "year" ADJ3 "old") OR ("100" ADJ3 "years" ADJ3 "old") OR ("101" ADJ3 "years" ADJ3 "old") OR ("102" ADJ3 "years" ADJ3 "old") OR ("103" ADJ3 "years" ADJ3 "old") OR ("104" ADJ3 "years" ADJ3 "old") OR ("105" ADJ3 "years" ADJ3 "old") OR ("106" ADJ3 "years" ADJ3 "old") OR ("107" ADJ3 "years" ADJ3 "old") OR ("108" ADJ3 "years" ADJ3 "old") OR ("109" ADJ3 "years" ADJ3 "old"**)).ti,ab))) NOT (exp "Animals"/ NOT exp "Humans"/)** NOT (("Case Report"/ OR "case report".ti OR "case report".af OR "Review"/ OR "review".ti) NOT ("Clinical Study"/ OR exp "Clinical Trial"/ OR "trial".ti OR "RCT".ti OR exp "systematic review"/ OR "systematic review".ti)) AND english.la

**Web of Science**

**(**(TS=("Wheelchair" OR "Wheelchairs" OR "Wheelchair" OR "Wheelchair*" OR "Wheel chairs" OR "Wheel chair" OR "Wheel chair*" OR "Mobility Scooter" OR "Mobility Scooters") AND TS=("Physical Activity" OR "Exercise" OR "Exercise" OR "Exercises" OR "Exercising" OR "Exercis*" OR "physical activity" OR "physical activities" OR "Endurance Training" OR "Exergaming" OR "Gymnastics" OR "Gymnastic" OR "Interval Training" OR "Jogging" OR "Motor Activity" OR "Movement" OR "Muscle Stretching"OR "Physical Conditioning" OR "Resistance Training" OR "Running" OR "Stair Climbing" OR "Swimming" OR "Training" OR "Walking" OR "Kinesiotherapy" OR "Physical Exertion" OR "Physical Exertions" OR "Physical Effort" OR "Physical Efforts" OR "Sport" OR "Sports" OR "Sport" OR "Athletic Performance" OR "Baseball" OR "Basketball" OR "Bicycling" OR "Boxing" OR "Cardiorespiratory Fitness" OR "Cardiorespiratory Fitness" OR "Cricket Sport" OR "Diving" OR "Football" OR "Golf" OR "Gymnastics" OR "Hockey" OR "Jogging" OR "Marathon Running" OR "Martial Arts" OR "Mountaineering" OR "Nordic Walking" OR "Physical Endurance" OR "Physical Fitness" OR "Racquet Sports" OR "Return to Sport" OR "Rugby" OR "Running" OR "Skating" OR "Skiing" OR "Snow Sports" OR "Soccer" OR "Swimming" OR "Tai Ji" OR "Team Sports" OR "Tennis" OR "Track and Field" OR "Volleyball" OR "Walking" OR "Water Sports" OR "Weight Lifting" OR "Wrestling" OR "Youth Sports" OR "Qigong" OR "Dance Therapy" OR "Tai Ji" OR "Yoga" OR "Physiotherapy" OR "Physical Therapy" OR "physiotherapy" OR "physiotherapy*" OR "Dry Needling" OR "Electrotherapy" OR "Shock wave Therapy" OR "Balneotherapy" OR "Musculoskeletal Manipulation" OR "Dry Needling" OR "Electric Stimulation Therapy" OR "Electroacupuncture" OR "Pulsed Radiofrequency Treatment" OR "Spinal Cord Stimulation" OR "Transcutaneous Electric Nerve Stimulation" OR "Extracorporeal Shockwave Therapy" OR "Hydrotherapy" OR "Aquatic Therapy" OR "Therapeutic Irrigation" OR "Musculoskeletal Manipulations" OR "Applied Kinesiology" OR "Orthopedic Manipulation" OR "Orthopaedic Manipulation" OR "Osteopathic Manipulation" OR "Spinal Manipulation" OR "Continuous Passive Motion Therapy" OR "Soft Tissue Therapy" OR "Acupressure" OR "Massage" OR "Manual Lymphatic Drainage" OR "Myofascial Release Therapy" OR "mobilization" OR "mobilization" OR "mobilisation" OR "mobilizing" OR "mobilising" OR "mobilize" OR "mobilise" OR "mobilized" OR "mobilised" OR "mobility") AND TS=("Ankle Fracture" OR "Femur Fracture" OR "Fibula Fracture" OR "Knee Fracture" OR "Tibia Fracture" OR "Ankle Fractures" OR "Femoral Fractures" OR "Hip Fractures" OR "Femoral Neck Fractures" OR "Proximal Femoral Fractures" OR "Hoffa Fracture" OR "Proximal Femoral Fractures" OR "Fibula Fractures" OR "Knee Fractures" OR "Hoffa Fracture" OR "Patella Fracture" OR "Tibial Plateau Fractures" OR "Tibial Fractures" OR "Tibial Plateau Fractures" OR "Ankle Fracture" OR "Femoral Fracture" OR "Hip Fracture" OR "Femoral Neck Fracture" OR "Proximal Femoral Fracture" OR "Hoffa Fractures" OR "Proximal Femoral Fracture" OR "Fibula Fracture" OR "Knee Fracture" OR "Hoffa Fractures" OR "Patella Fractures" OR "Tibial Plateau Fracture" OR "Tibial Fracture" OR "Tibial Plateau Fracture" OR (("Lower Limb" OR "lower limb" OR "lower limbs" OR "lower extremity" OR "lower extremities" OR "Ankle" OR "Foot" OR "Heel" OR "Hip" OR "Knee" OR "Leg" OR "Ankles" OR "Feet" OR "Heels" OR "Hips" OR "Knees" OR "Legs") AND ("Fracture" OR "fracture" OR "fractures" OR "fractur*")) OR "immobility" OR "immobility" OR "immobile" OR "immobil*" OR "temporary" OR "non weight bearing" OR "non weight bearing" OR "nonweight bearing" OR "non weight bear*" OR "nonweight bear*")**) OR** (TI=("Wheelchair" OR "Wheelchairs" OR "Wheelchair" OR "Wheelchair*" OR "Wheel chairs" OR "Wheel chair" OR "Wheel chair*" OR "Mobility Scooter" OR "Mobility Scooters") AND TI=("Physical Activity" OR "Exercise" OR "Exercise" OR "Exercises" OR "Exercising" OR "Exercis*" OR "physical activity" OR "physical activities" OR "Endurance Training" OR "Exergaming" OR "Gymnastics" OR "Gymnastic" OR "Interval Training" OR "Jogging" OR "Motor Activity" OR "Movement" OR "Muscle Stretching"OR "Physical Conditioning" OR "Resistance Training" OR "Running" OR "Stair Climbing" OR "Swimming" OR "Training" OR "Walking" OR "Kinesiotherapy" OR "Physical Exertion" OR "Physical Exertions" OR "Physical Effort" OR "Physical Efforts" OR "Sport" OR "Sports" OR "Sport" OR "Athletic Performance" OR "Baseball" OR "Basketball" OR "Bicycling" OR "Boxing" OR "Cardiorespiratory Fitness" OR "Cardiorespiratory Fitness" OR "Cricket Sport" OR "Diving" OR "Football" OR "Golf" OR "Gymnastics" OR "Hockey" OR "Jogging" OR "Marathon Running" OR "Martial Arts" OR "Mountaineering" OR "Nordic Walking" OR "Physical Endurance" OR "Physical Fitness" OR "Racquet Sports" OR "Return to Sport" OR "Rugby" OR "Running" OR "Skating" OR "Skiing" OR "Snow Sports" OR "Soccer" OR "Swimming" OR "Tai Ji" OR "Team Sports" OR "Tennis" OR "Track and Field" OR "Volleyball" OR "Walking" OR "Water Sports" OR "Weight Lifting" OR "Wrestling" OR "Youth Sports" OR "Qigong" OR "Dance Therapy" OR "Tai Ji" OR "Yoga" OR "Physiotherapy" OR "Physical Therapy" OR "physiotherapy" OR "physiotherapy*" OR "Dry Needling" OR "Electrotherapy" OR "Shock wave Therapy" OR "Balneotherapy" OR "Musculoskeletal Manipulation" OR "Dry Needling" OR "Electric Stimulation Therapy" OR "Electroacupuncture" OR "Pulsed Radiofrequency Treatment" OR "Spinal Cord Stimulation" OR "Transcutaneous Electric Nerve Stimulation" OR "Extracorporeal Shockwave Therapy" OR "Hydrotherapy" OR "Aquatic Therapy" OR "Therapeutic Irrigation" OR "Musculoskeletal Manipulations" OR "Applied Kinesiology" OR "Orthopedic Manipulation" OR "Orthopaedic Manipulation" OR "Osteopathic Manipulation" OR "Spinal Manipulation" OR "Continuous Passive Motion Therapy" OR "Soft Tissue Therapy" OR "Acupressure" OR "Massage" OR "Manual Lymphatic Drainage" OR "Myofascial Release Therapy" OR "mobilization" OR "mobilization" OR "mobilisation" OR "mobilizing" OR "mobilising" OR "mobilize" OR "mobilise" OR "mobilized" OR "mobilised" OR "mobility") AND TS=**("elderly" OR "elder" OR "elders" OR "geriatr*" OR "Home for the Aged" OR "Elderly care" OR "Senior Center" OR "Nonagenarians" OR "Nonagenarian" OR "Octogenarians" OR "Octogenarian" OR "Centenarians" OR "Centenarian" OR "septuagenarian" OR "septuagenarians" OR "geront*" OR "older person" OR "old person" OR "older patient" OR "old patient" OR "older persons" OR "old persons" OR "older patients" OR "old patients" OR "older women" OR "old women" OR "older men" OR "old men" OR "old adult" OR "older adult" OR "old adults" OR "older adults" OR "Older individual" OR "Older individuals" OR "old people" OR "older people" OR "Oldest Old" OR "older population" OR "aging population" OR "aging population" OR "old aged" OR "old age" OR (("older" NEAR/3 "person") OR ("old" NEAR/3 "person") OR ("older" NEAR/3 "patient") OR ("old" NEAR/3 "patient") OR ("older" NEAR/3 "persons") OR ("old" NEAR/3 "persons") OR ("older" NEAR/3 "patients") OR ("old" NEAR/3 "patients") OR ("older" NEAR/3 "women") OR ("old" NEAR/3 "women") OR ("older" NEAR/3 "men") OR ("old" NEAR/3 "men") OR ("old" NEAR/3 "adult") OR ("older" NEAR/3 "adult") OR ("old" NEAR/3 "adults") OR ("older" NEAR/3 "adults") OR ("Older" NEAR/3 "individual") OR ("Older" NEAR/3 "individuals") OR ("old" NEAR/3 "people") OR ("older" NEAR/3 "people") OR ("Oldest" NEAR/3 "Old") OR ("older" NEAR/3 "population") OR ("aging" NEAR/3 "population") OR ("aging" NEAR/3 "population") OR ("old" NEAR/3 "aged") OR ("old" NEAR/3 "age") OR (**"60" NEAR/3 "year" NEAR/3 "old") OR ("61" NEAR/3 "year" NEAR/3 "old") OR ("62" NEAR/3 "year" NEAR/3 "old") OR ("63" NEAR/3 "year" NEAR/3 "old") OR ("64" NEAR/3 "year" NEAR/3 "old") OR ("65" NEAR/3 "year" NEAR/3 "old") OR ("66" NEAR/3 "year" NEAR/3 "old") OR ("67" NEAR/3 "year" NEAR/3 "old") OR ("68" NEAR/3 "year" NEAR/3 "old") OR ("69" NEAR/3 "year" NEAR/3 "old") OR ("60" NEAR/3 "years" NEAR/3 "old") OR ("61" NEAR/3 "years" NEAR/3 "old") OR ("62" NEAR/3 "years" NEAR/3 "old") OR ("63" NEAR/3 "years" NEAR/3 "old") OR ("64" NEAR/3 "years" NEAR/3 "old") OR ("65" NEAR/3 "years" NEAR/3 "old") OR ("66" NEAR/3 "years" NEAR/3 "old") OR ("67" NEAR/3 "years" NEAR/3 "old") OR ("68" NEAR/3 "years" NEAR/3 "old") OR ("69" NEAR/3 "years" NEAR/3 "old") OR ("70" NEAR/3 "year" NEAR/3 "old") OR ("71" NEAR/3 "year" NEAR/3 "old") OR ("72" NEAR/3 "year" NEAR/3 "old") OR ("73" NEAR/3 "year" NEAR/3 "old") OR ("74" NEAR/3 "year" NEAR/3 "old") OR ("75" NEAR/3 "year" NEAR/3 "old") OR ("76" NEAR/3 "year" NEAR/3 "old") OR ("77" NEAR/3 "year" NEAR/3 "old") OR ("78" NEAR/3 "year" NEAR/3 "old") OR ("79" NEAR/3 "year" NEAR/3 "old") OR ("70" NEAR/3 "years" NEAR/3 "old") OR ("71" NEAR/3 "years" NEAR/3 "old") OR ("72" NEAR/3 "years" NEAR/3 "old") OR ("73" NEAR/3 "years" NEAR/3 "old") OR ("74" NEAR/3 "years" NEAR/3 "old") OR ("75" NEAR/3 "years" NEAR/3 "old") OR ("76" NEAR/3 "years" NEAR/3 "old") OR ("77" NEAR/3 "years" NEAR/3 "old") OR ("78" NEAR/3 "years" NEAR/3 "old") OR ("79" NEAR/3 "years" NEAR/3 "old") OR ("80" NEAR/3 "year" NEAR/3 "old") OR ("81" NEAR/3 "year" NEAR/3 "old") OR ("82" NEAR/3 "year" NEAR/3 "old") OR ("83" NEAR/3 "year" NEAR/3 "old") OR ("84" NEAR/3 "year" NEAR/3 "old") OR ("85" NEAR/3 "year" NEAR/3 "old") OR ("86" NEAR/3 "year" NEAR/3 "old") OR ("87" NEAR/3 "year" NEAR/3 "old") OR ("88" NEAR/3 "year" NEAR/3 "old") OR ("89" NEAR/3 "year" NEAR/3 "old") OR ("80" NEAR/3 "years" NEAR/3 "old") OR ("81" NEAR/3 "years" NEAR/3 "old") OR ("82" NEAR/3 "years" NEAR/3 "old") OR ("83" NEAR/3 "years" NEAR/3 "old") OR ("84" NEAR/3 "years" NEAR/3 "old") OR ("85" NEAR/3 "years" NEAR/3 "old") OR ("86" NEAR/3 "years" NEAR/3 "old") OR ("87" NEAR/3 "years" NEAR/3 "old") OR ("88" NEAR/3 "years" NEAR/3 "old") OR ("89" NEAR/3 "years" NEAR/3 "old") OR ("90" NEAR/3 "year" NEAR/3 "old") OR ("91" NEAR/3 "year" NEAR/3 "old") OR ("92" NEAR/3 "year" NEAR/3 "old") OR ("93" NEAR/3 "year" NEAR/3 "old") OR ("94" NEAR/3 "year" NEAR/3 "old") OR ("95" NEAR/3 "year" NEAR/3 "old") OR ("96" NEAR/3 "year" NEAR/3 "old") OR ("97" NEAR/3 "year" NEAR/3 "old") OR ("98" NEAR/3 "year" NEAR/3 "old") OR ("99" NEAR/3 "year" NEAR/3 "old") OR ("90" NEAR/3 "years" NEAR/3 "old") OR ("91" NEAR/3 "years" NEAR/3 "old") OR ("92" NEAR/3 "years" NEAR/3 "old") OR ("93" NEAR/3 "years" NEAR/3 "old") OR ("94" NEAR/3 "years" NEAR/3 "old") OR ("95" NEAR/3 "years" NEAR/3 "old") OR ("96" NEAR/3 "years" NEAR/3 "old") OR ("97" NEAR/3 "years" NEAR/3 "old") OR ("98" NEAR/3 "years" NEAR/3 "old") OR ("99" NEAR/3 "years" NEAR/3 "old") OR ("100" NEAR/3 "year" NEAR/3 "old") OR ("101" NEAR/3 "year" NEAR/3 "old") OR ("102" NEAR/3 "year" NEAR/3 "old") OR ("103" NEAR/3 "year" NEAR/3 "old") OR ("104" NEAR/3 "year" NEAR/3 "old") OR ("105" NEAR/3 "year" NEAR/3 "old") OR ("106" NEAR/3 "year" NEAR/3 "old") OR ("107" NEAR/3 "year" NEAR/3 "old") OR ("108" NEAR/3 "year" NEAR/3 "old") OR ("109" NEAR/3 "year" NEAR/3 "old") OR ("100" NEAR/3 "years" NEAR/3 "old") OR ("101" NEAR/3 "years" NEAR/3 "old") OR ("102" NEAR/3 "years" NEAR/3 "old") OR ("103" NEAR/3 "years" NEAR/3 "old") OR ("104" NEAR/3 "years" NEAR/3 "old") OR ("105" NEAR/3 "years" NEAR/3 "old") OR ("106" NEAR/3 "years" NEAR/3 "old") OR ("107" NEAR/3 "years" NEAR/3 "old") OR ("108" NEAR/3 "years" NEAR/3 "old") OR ("109" NEAR/3 "years" NEAR/3 "old"**)))) OR** (TS=("Physical Activity" OR "Exercise" OR "Exercise" OR "Exercises" OR "Exercising" OR "Exercis*" OR "physical activity" OR "physical activities" OR "Endurance Training" OR "Exergaming" OR "Gymnastics" OR "Gymnastic" OR "Interval Training" OR "Jogging" OR "Motor Activity" OR "Movement" OR "Muscle Stretching"OR "Physical Conditioning" OR "Resistance Training" OR "Running" OR "Stair Climbing" OR "Swimming" OR "Training" OR "Walking" OR "Kinesiotherapy" OR "Physical Exertion" OR "Physical Exertions" OR "Physical Effort" OR "Physical Efforts" OR "Sport" OR "Sports" OR "Sport" OR "Athletic Performance" OR "Baseball" OR "Basketball" OR "Bicycling" OR "Boxing" OR "Cardiorespiratory Fitness" OR "Cardiorespiratory Fitness" OR "Cricket Sport" OR "Diving" OR "Football" OR "Golf" OR "Gymnastics" OR "Hockey" OR "Jogging" OR "Marathon Running" OR "Martial Arts" OR "Mountaineering" OR "Nordic Walking" OR "Physical Endurance" OR "Physical Fitness" OR "Racquet Sports" OR "Return to Sport" OR "Rugby" OR "Running" OR "Skating" OR "Skiing" OR "Snow Sports" OR "Soccer" OR "Swimming" OR "Tai Ji" OR "Team Sports" OR "Tennis" OR "Track and Field" OR "Volleyball" OR "Walking" OR "Water Sports" OR "Weight Lifting" OR "Wrestling" OR "Youth Sports" OR "Qigong" OR "Dance Therapy" OR "Tai Ji" OR "Yoga" OR "Physiotherapy" OR "Physical Therapy" OR "physiotherapy" OR "physiotherapy*" OR "Dry Needling" OR "Electrotherapy" OR "Shock wave Therapy" OR "Balneotherapy" OR "Musculoskeletal Manipulation" OR "Dry Needling" OR "Electric Stimulation Therapy" OR "Electroacupuncture" OR "Pulsed Radiofrequency Treatment" OR "Spinal Cord Stimulation" OR "Transcutaneous Electric Nerve Stimulation" OR "Extracorporeal Shockwave Therapy" OR "Hydrotherapy" OR "Aquatic Therapy" OR "Therapeutic Irrigation" OR "Musculoskeletal Manipulations" OR "Applied Kinesiology" OR "Orthopedic Manipulation" OR "Orthopaedic Manipulation" OR "Osteopathic Manipulation" OR "Spinal Manipulation" OR "Continuous Passive Motion Therapy" OR "Soft Tissue Therapy" OR "Acupressure" OR "Massage" OR "Manual Lymphatic Drainage" OR "Myofascial Release Therapy" OR "mobilization" OR "mobilization" OR "mobilisation" OR "mobilizing" OR "mobilising" OR "mobilize" OR "mobilise" OR "mobilized" OR "mobilised" OR "mobility") AND TI=("Ankle Fracture" OR "Femur Fracture" OR "Fibula Fracture" OR "Knee Fracture" OR "Tibia Fracture" OR "Ankle Fractures" OR "Femoral Fractures" OR "Hip Fractures" OR "Femoral Neck Fractures" OR "Proximal Femoral Fractures" OR "Hoffa Fracture" OR "Proximal Femoral Fractures" OR "Fibula Fractures" OR "Knee Fractures" OR "Hoffa Fracture" OR "Patella Fracture" OR "Tibial Plateau Fractures" OR "Tibial Fractures" OR "Tibial Plateau Fractures" OR "Ankle Fracture" OR "Femoral Fracture" OR "Hip Fracture" OR "Femoral Neck Fracture" OR "Proximal Femoral Fracture" OR "Hoffa Fractures" OR "Proximal Femoral Fracture" OR "Fibula Fracture" OR "Knee Fracture" OR "Hoffa Fractures" OR "Patella Fractures" OR "Tibial Plateau Fracture" OR "Tibial Fracture" OR "Tibial Plateau Fracture" OR (("Lower Limb" OR "lower limb" OR "lower limbs" OR "lower extremity" OR "lower extremities" OR "Ankle" OR "Foot" OR "Heel" OR "Hip" OR "Knee" OR "Leg" OR "Ankles" OR "Feet" OR "Heels" OR "Hips" OR "Knees" OR "Legs") AND ("Fracture" OR "fracture" OR "fractures" OR "fractur*")) OR **"acutely ill"**) AND TS=("immobility" OR "immobility" OR "immobile" OR "immobil*" OR "temporary" OR "non weight bearing" OR "non weight bearing" OR "nonweight bearing" OR "non weight bear*" OR "nonweight bear*" OR "physically dependent" OR "physically disabled" OR **"physically dependent" OR "physically disabled" OR "disuse" OR "bed rest" OR "bed rest" OR "bedrest" OR "leg immobilization" OR "leg immobilisation" OR "Weightlessness" OR "deconditioning" OR "deconditioning" OR "permissive weight-bearing"**)**) OR** (TI=("Physical Activity" OR "Exercise" OR "Exercise" OR "Exercises" OR "Exercising" OR "Exercis*" OR "physical activity" OR "physical activities" OR "Endurance Training" OR "Exergaming" OR "Gymnastics" OR "Gymnastic" OR "Interval Training" OR "Jogging" OR "Motor Activity" OR "Movement" OR "Muscle Stretching"OR "Physical Conditioning" OR "Resistance Training" OR "Running" OR "Stair Climbing" OR "Swimming" OR "Training" OR "Walking" OR "Kinesiotherapy" OR "Physical Exertion" OR "Physical Exertions" OR "Physical Effort" OR "Physical Efforts" OR "Sport" OR "Sports" OR "Sport" OR "Athletic Performance" OR "Baseball" OR "Basketball" OR "Bicycling" OR "Boxing" OR "Cardiorespiratory Fitness" OR "Cardiorespiratory Fitness" OR "Cricket Sport" OR "Diving" OR "Football" OR "Golf" OR "Gymnastics" OR "Hockey" OR "Jogging" OR "Marathon Running" OR "Martial Arts" OR "Mountaineering" OR "Nordic Walking" OR "Physical Endurance" OR "Physical Fitness" OR "Racquet Sports" OR "Return to Sport" OR "Rugby" OR "Running" OR "Skating" OR "Skiing" OR "Snow Sports" OR "Soccer" OR "Swimming" OR "Tai Ji" OR "Team Sports" OR "Tennis" OR "Track and Field" OR "Volleyball" OR "Walking" OR "Water Sports" OR "Weight Lifting" OR "Wrestling" OR "Youth Sports" OR "Qigong" OR "Dance Therapy" OR "Tai Ji" OR "Yoga" OR "Physiotherapy" OR "Physical Therapy" OR "physiotherapy" OR "physiotherapy*" OR "Dry Needling" OR "Electrotherapy" OR "Shock wave Therapy" OR "Balneotherapy" OR "Musculoskeletal Manipulation" OR "Dry Needling" OR "Electric Stimulation Therapy" OR "Electroacupuncture" OR "Pulsed Radiofrequency Treatment" OR "Spinal Cord Stimulation" OR "Transcutaneous Electric Nerve Stimulation" OR "Extracorporeal Shockwave Therapy" OR "Hydrotherapy" OR "Aquatic Therapy" OR "Therapeutic Irrigation" OR "Musculoskeletal Manipulations" OR "Applied Kinesiology" OR "Orthopedic Manipulation" OR "Orthopaedic Manipulation" OR "Osteopathic Manipulation" OR "Spinal Manipulation" OR "Continuous Passive Motion Therapy" OR "Soft Tissue Therapy" OR "Acupressure" OR "Massage" OR "Manual Lymphatic Drainage" OR "Myofascial Release Therapy" OR "mobilization" OR "mobilization" OR "mobilisation" OR "mobilizing" OR "mobilising" OR "mobilize" OR "mobilise" OR "mobilized" OR "mobilised" OR "mobility") AND TS=(**"disuse"**) AND TS=**("elderly" OR "elder" OR "elders" OR "geriatr*" OR "Home for the Aged" OR "Elderly care" OR "Senior Center" OR "Nonagenarians" OR "Nonagenarian" OR "Octogenarians" OR "Octogenarian" OR "Centenarians" OR "Centenarian" OR "septuagenarian" OR "septuagenarians" OR "geront*" OR "older person" OR "old person" OR "older patient" OR "old patient" OR "older persons" OR "old persons" OR "older patients" OR "old patients" OR "older women" OR "old women" OR "older men" OR "old men" OR "old adult" OR "older adult" OR "old adults" OR "older adults" OR "Older individual" OR "Older individuals" OR "old people" OR "older people" OR "Oldest Old" OR "older population" OR "aging population" OR "aging population" OR "old aged" OR "old age" OR (("older" NEAR/3 "person") OR ("old" NEAR/3 "person") OR ("older" NEAR/3 "patient") OR ("old" NEAR/3 "patient") OR ("older" NEAR/3 "persons") OR ("old" NEAR/3 "persons") OR ("older" NEAR/3 "patients") OR ("old" NEAR/3 "patients") OR ("older" NEAR/3 "women") OR ("old" NEAR/3 "women") OR ("older" NEAR/3 "men") OR ("old" NEAR/3 "men") OR ("old" NEAR/3 "adult") OR ("older" NEAR/3 "adult") OR ("old" NEAR/3 "adults") OR ("older" NEAR/3 "adults") OR ("Older" NEAR/3 "individual") OR ("Older" NEAR/3 "individuals") OR ("old" NEAR/3 "people") OR ("older" NEAR/3 "people") OR ("Oldest" NEAR/3 "Old") OR ("older" NEAR/3 "population") OR ("aging" NEAR/3 "population") OR ("aging" NEAR/3 "population") OR ("old" NEAR/3 "aged") OR ("old" NEAR/3 "age") OR (**"60" NEAR/3 "year" NEAR/3 "old") OR ("61" NEAR/3 "year" NEAR/3 "old") OR ("62" NEAR/3 "year" NEAR/3 "old") OR ("63" NEAR/3 "year" NEAR/3 "old") OR ("64" NEAR/3 "year" NEAR/3 "old") OR ("65" NEAR/3 "year" NEAR/3 "old") OR ("66" NEAR/3 "year" NEAR/3 "old") OR ("67" NEAR/3 "year" NEAR/3 "old") OR ("68" NEAR/3 "year" NEAR/3 "old") OR ("69" NEAR/3 "year" NEAR/3 "old") OR ("60" NEAR/3 "years" NEAR/3 "old") OR ("61" NEAR/3 "years" NEAR/3 "old") OR ("62" NEAR/3 "years" NEAR/3 "old") OR ("63" NEAR/3 "years" NEAR/3 "old") OR ("64" NEAR/3 "years" NEAR/3 "old") OR ("65" NEAR/3 "years" NEAR/3 "old") OR ("66" NEAR/3 "years" NEAR/3 "old") OR ("67" NEAR/3 "years" NEAR/3 "old") OR ("68" NEAR/3 "years" NEAR/3 "old") OR ("69" NEAR/3 "years" NEAR/3 "old") OR ("70" NEAR/3 "year" NEAR/3 "old") OR ("71" NEAR/3 "year" NEAR/3 "old") OR ("72" NEAR/3 "year" NEAR/3 "old") OR ("73" NEAR/3 "year" NEAR/3 "old") OR ("74" NEAR/3 "year" NEAR/3 "old") OR ("75" NEAR/3 "year" NEAR/3 "old") OR ("76" NEAR/3 "year" NEAR/3 "old") OR ("77" NEAR/3 "year" NEAR/3 "old") OR ("78" NEAR/3 "year" NEAR/3 "old") OR ("79" NEAR/3 "year" NEAR/3 "old") OR ("70" NEAR/3 "years" NEAR/3 "old") OR ("71" NEAR/3 "years" NEAR/3 "old") OR ("72" NEAR/3 "years" NEAR/3 "old") OR ("73" NEAR/3 "years" NEAR/3 "old") OR ("74" NEAR/3 "years" NEAR/3 "old") OR ("75" NEAR/3 "years" NEAR/3 "old") OR ("76" NEAR/3 "years" NEAR/3 "old") OR ("77" NEAR/3 "years" NEAR/3 "old") OR ("78" NEAR/3 "years" NEAR/3 "old") OR ("79" NEAR/3 "years" NEAR/3 "old") OR ("80" NEAR/3 "year" NEAR/3 "old") OR ("81" NEAR/3 "year" NEAR/3 "old") OR ("82" NEAR/3 "year" NEAR/3 "old") OR ("83" NEAR/3 "year" NEAR/3 "old") OR ("84" NEAR/3 "year" NEAR/3 "old") OR ("85" NEAR/3 "year" NEAR/3 "old") OR ("86" NEAR/3 "year" NEAR/3 "old") OR ("87" NEAR/3 "year" NEAR/3 "old") OR ("88" NEAR/3 "year" NEAR/3 "old") OR ("89" NEAR/3 "year" NEAR/3 "old") OR ("80" NEAR/3 "years" NEAR/3 "old") OR ("81" NEAR/3 "years" NEAR/3 "old") OR ("82" NEAR/3 "years" NEAR/3 "old") OR ("83" NEAR/3 "years" NEAR/3 "old") OR ("84" NEAR/3 "years" NEAR/3 "old") OR ("85" NEAR/3 "years" NEAR/3 "old") OR ("86" NEAR/3 "years" NEAR/3 "old") OR ("87" NEAR/3 "years" NEAR/3 "old") OR ("88" NEAR/3 "years" NEAR/3 "old") OR ("89" NEAR/3 "years" NEAR/3 "old") OR ("90" NEAR/3 "year" NEAR/3 "old") OR ("91" NEAR/3 "year" NEAR/3 "old") OR ("92" NEAR/3 "year" NEAR/3 "old") OR ("93" NEAR/3 "year" NEAR/3 "old") OR ("94" NEAR/3 "year" NEAR/3 "old") OR ("95" NEAR/3 "year" NEAR/3 "old") OR ("96" NEAR/3 "year" NEAR/3 "old") OR ("97" NEAR/3 "year" NEAR/3 "old") OR ("98" NEAR/3 "year" NEAR/3 "old") OR ("99" NEAR/3 "year" NEAR/3 "old") OR ("90" NEAR/3 "years" NEAR/3 "old") OR ("91" NEAR/3 "years" NEAR/3 "old") OR ("92" NEAR/3 "years" NEAR/3 "old") OR ("93" NEAR/3 "years" NEAR/3 "old") OR ("94" NEAR/3 "years" NEAR/3 "old") OR ("95" NEAR/3 "years" NEAR/3 "old") OR ("96" NEAR/3 "years" NEAR/3 "old") OR ("97" NEAR/3 "years" NEAR/3 "old") OR ("98" NEAR/3 "years" NEAR/3 "old") OR ("99" NEAR/3 "years" NEAR/3 "old") OR ("100" NEAR/3 "year" NEAR/3 "old") OR ("101" NEAR/3 "year" NEAR/3 "old") OR ("102" NEAR/3 "year" NEAR/3 "old") OR ("103" NEAR/3 "year" NEAR/3 "old") OR ("104" NEAR/3 "year" NEAR/3 "old") OR ("105" NEAR/3 "year" NEAR/3 "old") OR ("106" NEAR/3 "year" NEAR/3 "old") OR ("107" NEAR/3 "year" NEAR/3 "old") OR ("108" NEAR/3 "year" NEAR/3 "old") OR ("109" NEAR/3 "year" NEAR/3 "old") OR ("100" NEAR/3 "years" NEAR/3 "old") OR ("101" NEAR/3 "years" NEAR/3 "old") OR ("102" NEAR/3 "years" NEAR/3 "old") OR ("103" NEAR/3 "years" NEAR/3 "old") OR ("104" NEAR/3 "years" NEAR/3 "old") OR ("105" NEAR/3 "years" NEAR/3 "old") OR ("106" NEAR/3 "years" NEAR/3 "old") OR ("107" NEAR/3 "years" NEAR/3 "old") OR ("108" NEAR/3 "years" NEAR/3 "old") OR ("109" NEAR/3 "years" NEAR/3 "old"**))))) NOT TI=("veterinary" OR "rabbit" OR "rabbits" OR "animal" OR "animals" OR "mouse" OR "mice" OR "rodent" OR "rodents" OR "rat" OR "rats" OR "pig" OR "pigs" OR "porcine" OR "horse" OR "horses" OR "equine" OR "cow" OR "cows" OR "bovine" OR "goat" OR "goats" OR "sheep" OR "ovine" OR "canine" OR "dog" OR "dogs" OR "feline" OR "cat" OR "cats")** NOT TS=(("Case Report" OR "case report" OR "case report" OR "Review" OR "review") NOT ("Clinical Study" OR "Clinical Trial" OR "trial" OR "RCT" OR "systematic review" OR "systematic review")) AND LA=english

**Cochrane**

Process the four permutations separately

Manual check of English language

**(**

(("Wheelchair" OR "Wheelchairs" OR "Wheelchair" OR "Wheelchair*" OR "Wheel chairs" OR "Wheel chair" OR "Wheel chair*" OR "Mobility Scooter" OR "Mobility Scooters")**:ti,ab,kw** AND ("Physical Activity" OR "Exercise" OR "Exercise" OR "Exercises" OR "Exercising" OR "Exercis*" OR "physical activity" OR "physical activities" OR "Endurance Training" OR "Exergaming" OR "Gymnastics" OR "Gymnastic" OR "Interval Training" OR "Jogging" OR "Motor Activity" OR "Movement" OR "Muscle Stretching"OR "Physical Conditioning" OR "Resistance Training" OR "Running" OR "Stair Climbing" OR "Swimming" OR "Training" OR "Walking" OR "Kinesiotherapy" OR "Physical Exertion" OR "Physical Exertions" OR "Physical Effort" OR "Physical Efforts" OR "Sport" OR "Sports" OR "Sport" OR "Athletic Performance" OR "Baseball" OR "Basketball" OR "Bicycling" OR "Boxing" OR "Cardiorespiratory Fitness" OR "Cardiorespiratory Fitness" OR "Cricket Sport" OR "Diving" OR "Football" OR "Golf" OR "Gymnastics" OR "Hockey" OR "Jogging" OR "Marathon Running" OR "Martial Arts" OR "Mountaineering" OR "Nordic Walking" OR "Physical Endurance" OR "Physical Fitness" OR "Racquet Sports" OR "Return to Sport" OR "Rugby" OR "Running" OR "Skating" OR "Skiing" OR "Snow Sports" OR "Soccer" OR "Swimming" OR "Tai Ji" OR "Team Sports" OR "Tennis" OR "Track and Field" OR "Volleyball" OR "Walking" OR "Water Sports" OR "Weight Lifting" OR "Wrestling" OR "Youth Sports" OR "Qigong" OR "Dance Therapy" OR "Tai Ji" OR "Yoga" OR "Physiotherapy" OR "Physical Therapy" OR "physiotherapy" OR "physiotherapy*" OR "Dry Needling" OR "Electrotherapy" OR "Shock wave Therapy" OR "Balneotherapy" OR "Musculoskeletal Manipulation" OR "Dry Needling" OR "Electric Stimulation Therapy" OR "Electroacupuncture" OR "Pulsed Radiofrequency Treatment" OR "Spinal Cord Stimulation" OR "Transcutaneous Electric Nerve Stimulation" OR "Extracorporeal Shockwave Therapy" OR "Hydrotherapy" OR "Aquatic Therapy" OR "Therapeutic Irrigation" OR "Musculoskeletal Manipulations" OR "Applied Kinesiology" OR "Orthopedic Manipulation" OR "Orthopaedic Manipulation" OR "Osteopathic Manipulation" OR "Spinal Manipulation" OR "Continuous Passive Motion Therapy" OR "Soft Tissue Therapy" OR "Acupressure" OR "Massage" OR "Manual Lymphatic Drainage" OR "Myofascial Release Therapy" OR "mobilization" OR "mobilization" OR "mobilisation" OR "mobilizing" OR "mobilising" OR "mobilize" OR "mobilise" OR "mobilized" OR "mobilised" OR "mobility")**:ti,ab,kw** AND ("Ankle Fracture" OR "Femur Fracture" OR "Fibula Fracture" OR "Knee Fracture" OR "Tibia Fracture" OR "Ankle Fractures" OR "Femoral Fractures" OR "Hip Fractures" OR "Femoral Neck Fractures" OR "Proximal Femoral Fractures" OR "Hoffa Fracture" OR "Proximal Femoral Fractures" OR "Fibula Fractures" OR "Knee Fractures" OR "Hoffa Fracture" OR "Patella Fracture" OR "Tibial Plateau Fractures" OR "Tibial Fractures" OR "Tibial Plateau Fractures" OR "Ankle Fracture" OR "Femoral Fracture" OR "Hip Fracture" OR "Femoral Neck Fracture" OR "Proximal Femoral Fracture" OR "Hoffa Fractures" OR "Proximal Femoral Fracture" OR "Fibula Fracture" OR "Knee Fracture" OR "Hoffa Fractures" OR "Patella Fractures" OR "Tibial Plateau Fracture" OR "Tibial Fracture" OR "Tibial Plateau Fracture" OR (("Lower Limb" OR "lower limb" OR "lower limbs" OR "lower extremity" OR "lower extremities" OR "Ankle" OR "Foot" OR "Heel" OR "Hip" OR "Knee" OR "Leg" OR "Ankles" OR "Feet" OR "Heels" OR "Hips" OR "Knees" OR "Legs") AND ("Fracture" OR "fracture" OR "fractures" OR "fractur*")) OR "immobility" OR "immobility" OR "immobile" OR "immobil*" OR "temporary" OR "non weight bearing" OR "non weight bearing" OR "nonweight bearing" OR "non weight bear*" OR "nonweight bear*")**:ti,ab,kw)**

**OR**

(("Wheelchair" OR "Wheelchairs" OR "Wheelchair" OR "Wheelchair*" OR "Wheel chairs" OR "Wheel chair" OR "Wheel chair*" OR "Mobility Scooter" OR "Mobility Scooters")**:ti** AND ("Physical Activity" OR "Exercise" OR "Exercise" OR "Exercises" OR "Exercising" OR "Exercis*" OR "physical activity" OR "physical activities" OR "Endurance Training" OR "Exergaming" OR "Gymnastics" OR "Gymnastic" OR "Interval Training" OR "Jogging" OR "Motor Activity" OR "Movement" OR "Muscle Stretching"OR "Physical Conditioning" OR "Resistance Training" OR "Running" OR "Stair Climbing" OR "Swimming" OR "Training" OR "Walking" OR "Kinesiotherapy" OR "Physical Exertion" OR "Physical Exertions" OR "Physical Effort" OR "Physical Efforts" OR "Sport" OR "Sports" OR "Sport" OR "Athletic Performance" OR "Baseball" OR "Basketball" OR "Bicycling" OR "Boxing" OR "Cardiorespiratory Fitness" OR "Cardiorespiratory Fitness" OR "Cricket Sport" OR "Diving" OR "Football" OR "Golf" OR "Gymnastics" OR "Hockey" OR "Jogging" OR "Marathon Running" OR "Martial Arts" OR "Mountaineering" OR "Nordic Walking" OR "Physical Endurance" OR "Physical Fitness" OR "Racquet Sports" OR "Return to Sport" OR "Rugby" OR "Running" OR "Skating" OR "Skiing" OR "Snow Sports" OR "Soccer" OR "Swimming" OR "Tai Ji" OR "Team Sports" OR "Tennis" OR "Track and Field" OR "Volleyball" OR "Walking" OR "Water Sports" OR "Weight Lifting" OR "Wrestling" OR "Youth Sports" OR "Qigong" OR "Dance Therapy" OR "Tai Ji" OR "Yoga" OR "Physiotherapy" OR "Physical Therapy" OR "physiotherapy" OR "physiotherapy*" OR "Dry Needling" OR "Electrotherapy" OR "Shock wave Therapy" OR "Balneotherapy" OR "Musculoskeletal Manipulation" OR "Dry Needling" OR "Electric Stimulation Therapy" OR "Electroacupuncture" OR "Pulsed Radiofrequency Treatment" OR "Spinal Cord Stimulation" OR "Transcutaneous Electric Nerve Stimulation" OR "Extracorporeal Shockwave Therapy" OR "Hydrotherapy" OR "Aquatic Therapy" OR "Therapeutic Irrigation" OR "Musculoskeletal Manipulations" OR "Applied Kinesiology" OR "Orthopedic Manipulation" OR "Orthopaedic Manipulation" OR "Osteopathic Manipulation" OR "Spinal Manipulation" OR "Continuous Passive Motion Therapy" OR "Soft Tissue Therapy" OR "Acupressure" OR "Massage" OR "Manual Lymphatic Drainage" OR "Myofascial Release Therapy" OR "mobilization" OR "mobilization" OR "mobilisation" OR "mobilizing" OR "mobilising" OR "mobilize" OR "mobilise" OR "mobilized" OR "mobilised" OR "mobility")**:ti** AND **("elderly" OR "elder" OR "elders" OR "geriatr*" OR "Home for the Aged" OR "Elderly care" OR "Senior Center" OR "Nonagenarians" OR "Nonagenarian" OR "Octogenarians" OR "Octogenarian" OR "Centenarians" OR "Centenarian" OR "septuagenarian" OR "septuagenarians" OR "geront*" OR "older person" OR "old person" OR "older patient" OR "old patient" OR "older persons" OR "old persons" OR "older patients" OR "old patients" OR "older women" OR "old women" OR "older men" OR "old men" OR "old adult" OR "older adult" OR "old adults" OR "older adults" OR "Older individual" OR "Older individuals" OR "old people" OR "older people" OR "Oldest Old" OR "older population" OR "aging population" OR "aging population" OR "old aged" OR "old age" OR (("older" NEAR/3 "person") OR ("old" NEAR/3 "person") OR ("older" NEAR/3 "patient") OR ("old" NEAR/3 "patient") OR ("older" NEAR/3 "persons") OR ("old" NEAR/3 "persons") OR ("older" NEAR/3 "patients") OR ("old" NEAR/3 "patients") OR ("older" NEAR/3 "women") OR ("old" NEAR/3 "women") OR ("older" NEAR/3 "men") OR ("old" NEAR/3 "men") OR ("old" NEAR/3 "adult") OR ("older" NEAR/3 "adult") OR ("old" NEAR/3 "adults") OR ("older" NEAR/3 "adults") OR ("Older" NEAR/3 "individual") OR ("Older" NEAR/3 "individuals") OR ("old" NEAR/3 "people") OR ("older" NEAR/3 "people") OR ("Oldest" NEAR/3 "Old") OR ("older" NEAR/3 "population") OR ("aging" NEAR/3 "population") OR ("aging" NEAR/3 "population") OR ("old" NEAR/3 "aged") OR ("old" NEAR/3 "age") OR (**"60" NEAR/3 "year" NEAR/3 "old") OR ("61" NEAR/3 "year" NEAR/3 "old") OR ("62" NEAR/3 "year" NEAR/3 "old") OR ("63" NEAR/3 "year" NEAR/3 "old") OR ("64" NEAR/3 "year" NEAR/3 "old") OR ("65" NEAR/3 "year" NEAR/3 "old") OR ("66" NEAR/3 "year" NEAR/3 "old") OR ("67" NEAR/3 "year" NEAR/3 "old") OR ("68" NEAR/3 "year" NEAR/3 "old") OR ("69" NEAR/3 "year" NEAR/3 "old") OR ("60" NEAR/3 "years" NEAR/3 "old") OR ("61" NEAR/3 "years" NEAR/3 "old") OR ("62" NEAR/3 "years" NEAR/3 "old") OR ("63" NEAR/3 "years" NEAR/3 "old") OR ("64" NEAR/3 "years" NEAR/3 "old") OR ("65" NEAR/3 "years" NEAR/3 "old") OR ("66" NEAR/3 "years" NEAR/3 "old") OR ("67" NEAR/3 "years" NEAR/3 "old") OR ("68" NEAR/3 "years" NEAR/3 "old") OR ("69" NEAR/3 "years" NEAR/3 "old") OR ("70" NEAR/3 "year" NEAR/3 "old") OR ("71" NEAR/3 "year" NEAR/3 "old") OR ("72" NEAR/3 "year" NEAR/3 "old") OR ("73" NEAR/3 "year" NEAR/3 "old") OR ("74" NEAR/3 "year" NEAR/3 "old") OR ("75" NEAR/3 "year" NEAR/3 "old") OR ("76" NEAR/3 "year" NEAR/3 "old") OR ("77" NEAR/3 "year" NEAR/3 "old") OR ("78" NEAR/3 "year" NEAR/3 "old") OR ("79" NEAR/3 "year" NEAR/3 "old") OR ("70" NEAR/3 "years" NEAR/3 "old") OR ("71" NEAR/3 "years" NEAR/3 "old") OR ("72" NEAR/3 "years" NEAR/3 "old") OR ("73" NEAR/3 "years" NEAR/3 "old") OR ("74" NEAR/3 "years" NEAR/3 "old") OR ("75" NEAR/3 "years" NEAR/3 "old") OR ("76" NEAR/3 "years" NEAR/3 "old") OR ("77" NEAR/3 "years" NEAR/3 "old") OR ("78" NEAR/3 "years" NEAR/3 "old") OR ("79" NEAR/3 "years" NEAR/3 "old") OR ("80" NEAR/3 "year" NEAR/3 "old") OR ("81" NEAR/3 "year" NEAR/3 "old") OR ("82" NEAR/3 "year" NEAR/3 "old") OR ("83" NEAR/3 "year" NEAR/3 "old") OR ("84" NEAR/3 "year" NEAR/3 "old") OR ("85" NEAR/3 "year" NEAR/3 "old") OR ("86" NEAR/3 "year" NEAR/3 "old") OR ("87" NEAR/3 "year" NEAR/3 "old") OR ("88" NEAR/3 "year" NEAR/3 "old") OR ("89" NEAR/3 "year" NEAR/3 "old") OR ("80" NEAR/3 "years" NEAR/3 "old") OR ("81" NEAR/3 "years" NEAR/3 "old") OR ("82" NEAR/3 "years" NEAR/3 "old") OR ("83" NEAR/3 "years" NEAR/3 "old") OR ("84" NEAR/3 "years" NEAR/3 "old") OR ("85" NEAR/3 "years" NEAR/3 "old") OR ("86" NEAR/3 "years" NEAR/3 "old") OR ("87" NEAR/3 "years" NEAR/3 "old") OR ("88" NEAR/3 "years" NEAR/3 "old") OR ("89" NEAR/3 "years" NEAR/3 "old") OR ("90" NEAR/3 "year" NEAR/3 "old") OR ("91" NEAR/3 "year" NEAR/3 "old") OR ("92" NEAR/3 "year" NEAR/3 "old") OR ("93" NEAR/3 "year" NEAR/3 "old") OR ("94" NEAR/3 "year" NEAR/3 "old") OR ("95" NEAR/3 "year" NEAR/3 "old") OR ("96" NEAR/3 "year" NEAR/3 "old") OR ("97" NEAR/3 "year" NEAR/3 "old") OR ("98" NEAR/3 "year" NEAR/3 "old") OR ("99" NEAR/3 "year" NEAR/3 "old") OR ("90" NEAR/3 "years" NEAR/3 "old") OR ("91" NEAR/3 "years" NEAR/3 "old") OR ("92" NEAR/3 "years" NEAR/3 "old") OR ("93" NEAR/3 "years" NEAR/3 "old") OR ("94" NEAR/3 "years" NEAR/3 "old") OR ("95" NEAR/3 "years" NEAR/3 "old") OR ("96" NEAR/3 "years" NEAR/3 "old") OR ("97" NEAR/3 "years" NEAR/3 "old") OR ("98" NEAR/3 "years" NEAR/3 "old") OR ("99" NEAR/3 "years" NEAR/3 "old") OR ("100" NEAR/3 "year" NEAR/3 "old") OR ("101" NEAR/3 "year" NEAR/3 "old") OR ("102" NEAR/3 "year" NEAR/3 "old") OR ("103" NEAR/3 "year" NEAR/3 "old") OR ("104" NEAR/3 "year" NEAR/3 "old") OR ("105" NEAR/3 "year" NEAR/3 "old") OR ("106" NEAR/3 "year" NEAR/3 "old") OR ("107" NEAR/3 "year" NEAR/3 "old") OR ("108" NEAR/3 "year" NEAR/3 "old") OR ("109" NEAR/3 "year" NEAR/3 "old") OR ("100" NEAR/3 "years" NEAR/3 "old") OR ("101" NEAR/3 "years" NEAR/3 "old") OR ("102" NEAR/3 "years" NEAR/3 "old") OR ("103" NEAR/3 "years" NEAR/3 "old") OR ("104" NEAR/3 "years" NEAR/3 "old") OR ("105" NEAR/3 "years" NEAR/3 "old") OR ("106" NEAR/3 "years" NEAR/3 "old") OR ("107" NEAR/3 "years" NEAR/3 "old") OR ("108" NEAR/3 "years" NEAR/3 "old") OR ("109" NEAR/3 "years" NEAR/3 "old"**))):ti,ab,kw)**

**OR**

(("Physical Activity" OR "Exercise" OR "Exercise" OR "Exercises" OR "Exercising" OR "Exercis*" OR "physical activity" OR "physical activities" OR "Endurance Training" OR "Exergaming" OR "Gymnastics" OR "Gymnastic" OR "Interval Training" OR "Jogging" OR "Motor Activity" OR "Movement" OR "Muscle Stretching"OR "Physical Conditioning" OR "Resistance Training" OR "Running" OR "Stair Climbing" OR "Swimming" OR "Training" OR "Walking" OR "Kinesiotherapy" OR "Physical Exertion" OR "Physical Exertions" OR "Physical Effort" OR "Physical Efforts" OR "Sport" OR "Sports" OR "Sport" OR "Athletic Performance" OR "Baseball" OR "Basketball" OR "Bicycling" OR "Boxing" OR "Cardiorespiratory Fitness" OR "Cardiorespiratory Fitness" OR "Cricket Sport" OR "Diving" OR "Football" OR "Golf" OR "Gymnastics" OR "Hockey" OR "Jogging" OR "Marathon Running" OR "Martial Arts" OR "Mountaineering" OR "Nordic Walking" OR "Physical Endurance" OR "Physical Fitness" OR "Racquet Sports" OR "Return to Sport" OR "Rugby" OR "Running" OR "Skating" OR "Skiing" OR "Snow Sports" OR "Soccer" OR "Swimming" OR "Tai Ji" OR "Team Sports" OR "Tennis" OR "Track and Field" OR "Volleyball" OR "Walking" OR "Water Sports" OR "Weight Lifting" OR "Wrestling" OR "Youth Sports" OR "Qigong" OR "Dance Therapy" OR "Tai Ji" OR "Yoga" OR "Physiotherapy" OR "Physical Therapy" OR "physiotherapy" OR "physiotherapy*" OR "Dry Needling" OR "Electrotherapy" OR "Shock wave Therapy" OR "Balneotherapy" OR "Musculoskeletal Manipulation" OR "Dry Needling" OR "Electric Stimulation Therapy" OR "Electroacupuncture" OR "Pulsed Radiofrequency Treatment" OR "Spinal Cord Stimulation" OR "Transcutaneous Electric Nerve Stimulation" OR "Extracorporeal Shockwave Therapy" OR "Hydrotherapy" OR "Aquatic Therapy" OR "Therapeutic Irrigation" OR "Musculoskeletal Manipulations" OR "Applied Kinesiology" OR "Orthopedic Manipulation" OR "Orthopaedic Manipulation" OR "Osteopathic Manipulation" OR "Spinal Manipulation" OR "Continuous Passive Motion Therapy" OR "Soft Tissue Therapy" OR "Acupressure" OR "Massage" OR "Manual Lymphatic Drainage" OR "Myofascial Release Therapy" OR "mobilization" OR "mobilization" OR "mobilisation" OR "mobilizing" OR "mobilising" OR "mobilize" OR "mobilise" OR "mobilized" OR "mobilised" OR "mobility")**:ti,ab,kw** AND ("Ankle Fracture" OR "Femur Fracture" OR "Fibula Fracture" OR "Knee Fracture" OR "Tibia Fracture" OR "Ankle Fractures" OR "Femoral Fractures" OR "Hip Fractures" OR "Femoral Neck Fractures" OR "Proximal Femoral Fractures" OR "Hoffa Fracture" OR "Proximal Femoral Fractures" OR "Fibula Fractures" OR "Knee Fractures" OR "Hoffa Fracture" OR "Patella Fracture" OR "Tibial Plateau Fractures" OR "Tibial Fractures" OR "Tibial Plateau Fractures" OR "Ankle Fracture" OR "Femoral Fracture" OR "Hip Fracture" OR "Femoral Neck Fracture" OR "Proximal Femoral Fracture" OR "Hoffa Fractures" OR "Proximal Femoral Fracture" OR "Fibula Fracture" OR "Knee Fracture" OR "Hoffa Fractures" OR "Patella Fractures" OR "Tibial Plateau Fracture" OR "Tibial Fracture" OR "Tibial Plateau Fracture" OR (("Lower Limb" OR "lower limb" OR "lower limbs" OR "lower extremity" OR "lower extremities" OR "Ankle" OR "Foot" OR "Heel" OR "Hip" OR "Knee" OR "Leg" OR "Ankles" OR "Feet" OR "Heels" OR "Hips" OR "Knees" OR "Legs") AND ("Fracture" OR "fracture" OR "fractures" OR "fractur*")) OR **"acutely ill"**)**:ti** AND ("immobility" OR "immobility" OR "immobile" OR "immobil*" OR "temporary" OR "non weight bearing" OR "non weight bearing" OR "nonweight bearing" OR "non weight bear*" OR "nonweight bear*" OR "physically dependent" OR "physically disabled" OR **"physically dependent" OR "physically disabled" OR "disuse" OR "bed rest" OR "bed rest" OR "bedrest" OR "leg immobilization" OR "leg immobilisation" OR "Weightlessness" OR "deconditioning" OR "deconditioning" OR "permissive weight-bearing"**)**:ti,ab,kw)**

**OR**

(("Physical Activity" OR "Exercise" OR "Exercise" OR "Exercises" OR "Exercising" OR "Exercis*" OR "physical activity" OR "physical activities" OR "Endurance Training" OR "Exergaming" OR "Gymnastics" OR "Gymnastic" OR "Interval Training" OR "Jogging" OR "Motor Activity" OR "Movement" OR "Muscle Stretching"OR "Physical Conditioning" OR "Resistance Training" OR "Running" OR "Stair Climbing" OR "Swimming" OR "Training" OR "Walking" OR "Kinesiotherapy" OR "Physical Exertion" OR "Physical Exertions" OR "Physical Effort" OR "Physical Efforts" OR "Sport" OR "Sports" OR "Sport" OR "Athletic Performance" OR "Baseball" OR "Basketball" OR "Bicycling" OR "Boxing" OR "Cardiorespiratory Fitness" OR "Cardiorespiratory Fitness" OR "Cricket Sport" OR "Diving" OR "Football" OR "Golf" OR "Gymnastics" OR "Hockey" OR "Jogging" OR "Marathon Running" OR "Martial Arts" OR "Mountaineering" OR "Nordic Walking" OR "Physical Endurance" OR "Physical Fitness" OR "Racquet Sports" OR "Return to Sport" OR "Rugby" OR "Running" OR "Skating" OR "Skiing" OR "Snow Sports" OR "Soccer" OR "Swimming" OR "Tai Ji" OR "Team Sports" OR "Tennis" OR "Track and Field" OR "Volleyball" OR "Walking" OR "Water Sports" OR "Weight Lifting" OR "Wrestling" OR "Youth Sports" OR "Qigong" OR "Dance Therapy" OR "Tai Ji" OR "Yoga" OR "Physiotherapy" OR "Physical Therapy" OR "physiotherapy" OR "physiotherapy*" OR "Dry Needling" OR "Electrotherapy" OR "Shock wave Therapy" OR "Balneotherapy" OR "Musculoskeletal Manipulation" OR "Dry Needling" OR "Electric Stimulation Therapy" OR "Electroacupuncture" OR "Pulsed Radiofrequency Treatment" OR "Spinal Cord Stimulation" OR "Transcutaneous Electric Nerve Stimulation" OR "Extracorporeal Shockwave Therapy" OR "Hydrotherapy" OR "Aquatic Therapy" OR "Therapeutic Irrigation" OR "Musculoskeletal Manipulations" OR "Applied Kinesiology" OR "Orthopedic Manipulation" OR "Orthopaedic Manipulation" OR "Osteopathic Manipulation" OR "Spinal Manipulation" OR "Continuous Passive Motion Therapy" OR "Soft Tissue Therapy" OR "Acupressure" OR "Massage" OR "Manual Lymphatic Drainage" OR "Myofascial Release Therapy" OR "mobilization" OR "mobilization" OR "mobilisation" OR "mobilizing" OR "mobilising" OR "mobilize" OR "mobilise" OR "mobilized" OR "mobilised" OR "mobility")**:ti** AND (**"disuse"**)**:ti,ab,kw** AND **("elderly" OR "elder" OR "elders" OR "geriatr*" OR "Home for the Aged" OR "Elderly care" OR "Senior Center" OR "Nonagenarians" OR "Nonagenarian" OR "Octogenarians" OR "Octogenarian" OR "Centenarians" OR "Centenarian" OR "septuagenarian" OR "septuagenarians" OR "geront*" OR "older person" OR "old person" OR "older patient" OR "old patient" OR "older persons" OR "old persons" OR "older patients" OR "old patients" OR "older women" OR "old women" OR "older men" OR "old men" OR "old adult" OR "older adult" OR "old adults" OR "older adults" OR "Older individual" OR "Older individuals" OR "old people" OR "older people" OR "Oldest Old" OR "older population" OR "aging population" OR "aging population" OR "old aged" OR "old age" OR (("older" NEAR/3 "person") OR ("old" NEAR/3 "person") OR ("older" NEAR/3 "patient") OR ("old" NEAR/3 "patient") OR ("older" NEAR/3 "persons") OR ("old" NEAR/3 "persons") OR ("older" NEAR/3 "patients") OR ("old" NEAR/3 "patients") OR ("older" NEAR/3 "women") OR ("old" NEAR/3 "women") OR ("older" NEAR/3 "men") OR ("old" NEAR/3 "men") OR ("old" NEAR/3 "adult") OR ("older" NEAR/3 "adult") OR ("old" NEAR/3 "adults") OR ("older" NEAR/3 "adults") OR ("Older" NEAR/3 "individual") OR ("Older" NEAR/3 "individuals") OR ("old" NEAR/3 "people") OR ("older" NEAR/3 "people") OR ("Oldest" NEAR/3 "Old") OR ("older" NEAR/3 "population") OR ("aging" NEAR/3 "population") OR ("aging" NEAR/3 "population") OR ("old" NEAR/3 "aged") OR ("old" NEAR/3 "age") OR (**"60" NEAR/3 "year" NEAR/3 "old") OR ("61" NEAR/3 "year" NEAR/3 "old") OR ("62" NEAR/3 "year" NEAR/3 "old") OR ("63" NEAR/3 "year" NEAR/3 "old") OR ("64" NEAR/3 "year" NEAR/3 "old") OR ("65" NEAR/3 "year" NEAR/3 "old") OR ("66" NEAR/3 "year" NEAR/3 "old") OR ("67" NEAR/3 "year" NEAR/3 "old") OR ("68" NEAR/3 "year" NEAR/3 "old") OR ("69" NEAR/3 "year" NEAR/3 "old") OR ("60" NEAR/3 "years" NEAR/3 "old") OR ("61" NEAR/3 "years" NEAR/3 "old") OR ("62" NEAR/3 "years" NEAR/3 "old") OR ("63" NEAR/3 "years" NEAR/3 "old") OR ("64" NEAR/3 "years" NEAR/3 "old") OR ("65" NEAR/3 "years" NEAR/3 "old") OR ("66" NEAR/3 "years" NEAR/3 "old") OR ("67" NEAR/3 "years" NEAR/3 "old") OR ("68" NEAR/3 "years" NEAR/3 "old") OR ("69" NEAR/3 "years" NEAR/3 "old") OR ("70" NEAR/3 "year" NEAR/3 "old") OR ("71" NEAR/3 "year" NEAR/3 "old") OR ("72" NEAR/3 "year" NEAR/3 "old") OR ("73" NEAR/3 "year" NEAR/3 "old") OR ("74" NEAR/3 "year" NEAR/3 "old") OR ("75" NEAR/3 "year" NEAR/3 "old") OR ("76" NEAR/3 "year" NEAR/3 "old") OR ("77" NEAR/3 "year" NEAR/3 "old") OR ("78" NEAR/3 "year" NEAR/3 "old") OR ("79" NEAR/3 "year" NEAR/3 "old") OR ("70" NEAR/3 "years" NEAR/3 "old") OR ("71" NEAR/3 "years" NEAR/3 "old") OR ("72" NEAR/3 "years" NEAR/3 "old") OR ("73" NEAR/3 "years" NEAR/3 "old") OR ("74" NEAR/3 "years" NEAR/3 "old") OR ("75" NEAR/3 "years" NEAR/3 "old") OR ("76" NEAR/3 "years" NEAR/3 "old") OR ("77" NEAR/3 "years" NEAR/3 "old") OR ("78" NEAR/3 "years" NEAR/3 "old") OR ("79" NEAR/3 "years" NEAR/3 "old") OR ("80" NEAR/3 "year" NEAR/3 "old") OR ("81" NEAR/3 "year" NEAR/3 "old") OR ("82" NEAR/3 "year" NEAR/3 "old") OR ("83" NEAR/3 "year" NEAR/3 "old") OR ("84" NEAR/3 "year" NEAR/3 "old") OR ("85" NEAR/3 "year" NEAR/3 "old") OR ("86" NEAR/3 "year" NEAR/3 "old") OR ("87" NEAR/3 "year" NEAR/3 "old") OR ("88" NEAR/3 "year" NEAR/3 "old") OR ("89" NEAR/3 "year" NEAR/3 "old") OR ("80" NEAR/3 "years" NEAR/3 "old") OR ("81" NEAR/3 "years" NEAR/3 "old") OR ("82" NEAR/3 "years" NEAR/3 "old") OR ("83" NEAR/3 "years" NEAR/3 "old") OR ("84" NEAR/3 "years" NEAR/3 "old") OR ("85" NEAR/3 "years" NEAR/3 "old") OR ("86" NEAR/3 "years" NEAR/3 "old") OR ("87" NEAR/3 "years" NEAR/3 "old") OR ("88" NEAR/3 "years" NEAR/3 "old") OR ("89" NEAR/3 "years" NEAR/3 "old") OR ("90" NEAR/3 "year" NEAR/3 "old") OR ("91" NEAR/3 "year" NEAR/3 "old") OR ("92" NEAR/3 "year" NEAR/3 "old") OR ("93" NEAR/3 "year" NEAR/3 "old") OR ("94" NEAR/3 "year" NEAR/3 "old") OR ("95" NEAR/3 "year" NEAR/3 "old") OR ("96" NEAR/3 "year" NEAR/3 "old") OR ("97" NEAR/3 "year" NEAR/3 "old") OR ("98" NEAR/3 "year" NEAR/3 "old") OR ("99" NEAR/3 "year" NEAR/3 "old") OR ("90" NEAR/3 "years" NEAR/3 "old") OR ("91" NEAR/3 "years" NEAR/3 "old") OR ("92" NEAR/3 "years" NEAR/3 "old") OR ("93" NEAR/3 "years" NEAR/3 "old") OR ("94" NEAR/3 "years" NEAR/3 "old") OR ("95" NEAR/3 "years" NEAR/3 "old") OR ("96" NEAR/3 "years" NEAR/3 "old") OR ("97" NEAR/3 "years" NEAR/3 "old") OR ("98" NEAR/3 "years" NEAR/3 "old") OR ("99" NEAR/3 "years" NEAR/3 "old") OR ("100" NEAR/3 "year" NEAR/3 "old") OR ("101" NEAR/3 "year" NEAR/3 "old") OR ("102" NEAR/3 "year" NEAR/3 "old") OR ("103" NEAR/3 "year" NEAR/3 "old") OR ("104" NEAR/3 "year" NEAR/3 "old") OR ("105" NEAR/3 "year" NEAR/3 "old") OR ("106" NEAR/3 "year" NEAR/3 "old") OR ("107" NEAR/3 "year" NEAR/3 "old") OR ("108" NEAR/3 "year" NEAR/3 "old") OR ("109" NEAR/3 "year" NEAR/3 "old") OR ("100" NEAR/3 "years" NEAR/3 "old") OR ("101" NEAR/3 "years" NEAR/3 "old") OR ("102" NEAR/3 "years" NEAR/3 "old") OR ("103" NEAR/3 "years" NEAR/3 "old") OR ("104" NEAR/3 "years" NEAR/3 "old") OR ("105" NEAR/3 "years" NEAR/3 "old") OR ("106" NEAR/3 "years" NEAR/3 "old") OR ("107" NEAR/3 "years" NEAR/3 "old") OR ("108" NEAR/3 "years" NEAR/3 "old") OR ("109" NEAR/3 "years" NEAR/3 "old"**))):ti,ab,kw)**

**)**

**Emcare**

**(**((exp *"Wheelchair"/ OR "Wheelchairs".ti,ab OR "Wheelchair".ti,ab OR "Wheelchair*".ti,ab OR "Wheel chairs".ti,ab OR "Wheel chair".ti,ab OR "Wheel chair*".ti,ab OR "Mobility Scooter".ti,ab OR "Mobility Scooters".ti,ab) AND (exp *"Physical Activity"/ OR exp *"Exercise"/ OR "Exercise".ti,ab OR "Exercises".ti,ab OR "Exercising".ti,ab OR "Exercis*".ti,ab OR "physical activity".ti,ab OR "physical activities".ti,ab OR "Endurance Training".ti,ab OR "Exergaming".ti,ab OR "Gymnastics".ti,ab OR "Gymnastic".ti,ab OR "Interval Training".ti,ab OR "Jogging".ti,ab OR "Motor Activity".ti,ab OR "Movement".ti,ab OR "Muscle Stretching ".ti,ab OR "Physical Conditioning".ti,ab OR "Resistance Training".ti,ab OR "Running".ti,ab OR "Stair Climbing".ti,ab OR "Swimming".ti,ab OR "Training".ti,ab OR "Walking".ti,ab OR exp *"Kinesiotherapy"/ OR "Physical Exertion".ti,ab OR "Physical Exertions".ti,ab OR "Physical Effort".ti,ab OR "Physical Efforts".ti,ab OR exp *"Sport"/ OR "Sports".ti,ab OR "Sport".ti,ab OR "Athletic Performance".ti,ab OR "Baseball".ti,ab OR "Basketball".ti,ab OR "Bicycling".ti,ab OR "Boxing".ti,ab OR "Cardiorespiratory Fitness".ti,ab OR "Cardiorespiratory Fitness".ti,ab OR "Cricket Sport".ti,ab OR "Diving".ti,ab OR "Football".ti,ab OR "Golf".ti,ab OR "Gymnastics".ti,ab OR "Hockey".ti,ab OR "Jogging".ti,ab OR "Marathon Running".ti,ab OR "Martial Arts".ti,ab OR "Mountaineering".ti,ab OR "Nordic Walking".ti,ab OR "Physical Endurance".ti,ab OR "Physical Fitness".ti,ab OR "Racquet Sports".ti,ab OR "Return to Sport".ti,ab OR "Rugby".ti,ab OR "Running".ti,ab OR "Skating".ti,ab OR "Skiing".ti,ab OR "Snow Sports".ti,ab OR "Soccer".ti,ab OR "Swimming".ti,ab OR "Tai Ji".ti,ab OR "Team Sports".ti,ab OR "Tennis".ti,ab OR "Track and Field".ti,ab OR "Volleyball".ti,ab OR "Walking".ti,ab OR "Water Sports".ti,ab OR "Weight Lifting".ti,ab OR "Wrestling".ti,ab OR "Youth Sports".ti,ab OR "Qigong".ti,ab OR "Dance Therapy".ti,ab OR "Tai Ji".ti,ab OR "Yoga".ti,ab OR exp *"Physiotherapy"/ OR "Physical Therapy".ti,ab OR "physiotherapy".ti,ab OR "physiotherapy*".ti,ab OR exp *"Dry Needling"/ OR exp *"Electrotherapy"/ OR exp *"Shock wave Therapy"/ OR exp *"Balneotherapy"/ OR exp *"Musculoskeletal Manipulation"/ OR "Dry Needling".ti,ab OR "Electric Stimulation Therapy".ti,ab OR "Electroacupuncture".ti,ab OR "Pulsed Radiofrequency Treatment".ti,ab OR "Spinal Cord Stimulation".ti,ab OR "Transcutaneous Electric Nerve Stimulation".ti,ab OR "Extracorporeal Shockwave Therapy".ti,ab OR "Hydrotherapy".ti,ab OR "Aquatic Therapy".ti,ab OR "Therapeutic Irrigation".ti,ab OR "Musculoskeletal Manipulations".ti,ab OR "Applied Kinesiology".ti,ab OR "Orthopedic Manipulation".ti,ab OR "Orthopaedic Manipulation".ti,ab OR "Osteopathic Manipulation".ti,ab OR "Spinal Manipulation".ti,ab OR "Continuous Passive Motion Therapy".ti,ab OR "Soft Tissue Therapy".ti,ab OR "Acupressure".ti,ab OR "Massage".ti,ab OR "Manual Lymphatic Drainage".ti,ab OR "Myofascial Release Therapy".ti,ab OR exp *"mobilization"/ OR "mobilization".ti,ab OR "mobilisation".ti,ab OR "mobilizing".ti,ab OR "mobilising".ti,ab OR "mobilize".ti,ab OR "mobilise".ti,ab OR "mobilized".ti,ab OR "mobilised".ti,ab OR "mobility".ti,ab) AND (exp *"Ankle Fracture"/ OR exp *"Femur Fracture"/ OR exp *"Fibula Fracture"/ OR exp *"Knee Fracture"/ OR exp *"Tibia Fracture"/ OR "Ankle Fractures".ti,ab OR "Femoral Fractures".ti,ab OR "Hip Fractures".ti,ab OR "Femoral Neck Fractures".ti,ab OR "Proximal Femoral Fractures".ti,ab OR "Hoffa Fracture".ti,ab OR "Proximal Femoral Fractures".ti,ab OR "Fibula Fractures".ti,ab OR "Knee Fractures".ti,ab OR "Hoffa Fracture".ti,ab OR "Patella Fracture".ti,ab OR "Tibial Plateau Fractures".ti,ab OR "Tibial Fractures".ti,ab OR "Tibial Plateau Fractures".ti,ab OR "Ankle Fracture".ti,ab OR "Femoral Fracture".ti,ab OR "Hip Fracture".ti,ab OR "Femoral Neck Fracture".ti,ab OR "Proximal Femoral Fracture".ti,ab OR "Hoffa Fractures".ti,ab OR "Proximal Femoral Fracture".ti,ab OR "Fibula Fracture".ti,ab OR "Knee Fracture".ti,ab OR "Hoffa Fractures".ti,ab OR "Patella Fractures".ti,ab OR "Tibial Plateau Fracture".ti,ab OR "Tibial Fracture".ti,ab OR "Tibial Plateau Fracture".ti,ab OR ((exp *"Lower Limb"/ OR "lower limb".ti,ab OR "lower limbs".ti,ab OR "lower extremity".ti,ab OR "lower extremities".ti,ab OR "Ankle".ti,ab OR "Foot".ti,ab OR "Heel".ti,ab OR "Hip".ti,ab OR "Knee".ti,ab OR "Leg".ti,ab OR "Ankles".ti,ab OR "Feet".ti,ab OR "Heels".ti,ab OR "Hips".ti,ab OR "Knees".ti,ab OR "Legs".ti,ab) AND (exp *"Fracture"/ OR "fracture".ti,ab OR "fractures".ti,ab OR "fractur*".ti,ab)) OR exp *"immobility"/ OR "immobility".ti,ab OR "immobile".ti,ab OR "immobil*".ti,ab OR "temporary".ti,ab OR exp *"non weight bearing"/ OR "non weight bearing".ti,ab OR "nonweight bearing".ti,ab OR "non weight bear*".ti,ab OR "nonweight bear*".ti,ab)**) OR** ((exp *"Wheelchair"/ OR "Wheelchairs".ti OR "Wheelchair".ti OR "Wheelchair*".ti OR "Wheel chairs".ti OR "Wheel chair".ti OR "Wheel chair*".ti OR "Mobility Scooter".ti OR "Mobility Scooters".ti) AND (exp *"Physical Activity"/ OR exp *"Exercise"/ OR "Exercise".ti OR "Exercises".ti OR "Exercising".ti OR "Exercis*".ti OR "physical activity".ti OR "physical activities".ti OR "Endurance Training".ti OR "Exergaming".ti OR "Gymnastics".ti OR "Gymnastic".ti OR "Interval Training".ti OR "Jogging".ti OR "Motor Activity".ti OR "Movement".ti OR "Muscle Stretching ".ti OR "Physical Conditioning".ti OR "Resistance Training".ti OR "Running".ti OR "Stair Climbing".ti OR "Swimming".ti OR "Training".ti OR "Walking".ti OR exp *"Kinesiotherapy"/ OR "Physical Exertion".ti OR "Physical Exertions".ti OR "Physical Effort".ti OR "Physical Efforts".ti OR exp *"Sport"/ OR "Sports".ti OR "Sport".ti OR "Athletic Performance".ti OR "Baseball".ti OR "Basketball".ti OR "Bicycling".ti OR "Boxing".ti OR "Cardiorespiratory Fitness".ti OR "Cardiorespiratory Fitness".ti OR "Cricket Sport".ti OR "Diving".ti OR "Football".ti OR "Golf".ti OR "Gymnastics".ti OR "Hockey".ti OR "Jogging".ti OR "Marathon Running".ti OR "Martial Arts".ti OR "Mountaineering".ti OR "Nordic Walking".ti OR "Physical Endurance".ti OR "Physical Fitness".ti OR "Racquet Sports".ti OR "Return to Sport".ti OR "Rugby".ti OR "Running".ti OR "Skating".ti OR "Skiing".ti OR "Snow Sports".ti OR "Soccer".ti OR "Swimming".ti OR "Tai Ji".ti OR "Team Sports".ti OR "Tennis".ti OR "Track and Field".ti OR "Volleyball".ti OR "Walking".ti OR "Water Sports".ti OR "Weight Lifting".ti OR "Wrestling".ti OR "Youth Sports".ti OR "Qigong".ti OR "Dance Therapy".ti OR "Tai Ji".ti OR "Yoga".ti OR exp *"Physiotherapy"/ OR "Physical Therapy".ti OR "physiotherapy".ti OR "physiotherapy*".ti OR exp *"Dry Needling"/ OR exp *"Electrotherapy"/ OR exp *"Shock wave Therapy"/ OR exp *"Balneotherapy"/ OR exp *"Musculoskeletal Manipulation"/ OR "Dry Needling".ti OR "Electric Stimulation Therapy".ti OR "Electroacupuncture".ti OR "Pulsed Radiofrequency Treatment".ti OR "Spinal Cord Stimulation".ti OR "Transcutaneous Electric Nerve Stimulation".ti OR "Extracorporeal Shockwave Therapy".ti OR "Hydrotherapy".ti OR "Aquatic Therapy".ti OR "Therapeutic Irrigation".ti OR "Musculoskeletal Manipulations".ti OR "Applied Kinesiology".ti OR "Orthopedic Manipulation".ti OR "Orthopaedic Manipulation".ti OR "Osteopathic Manipulation".ti OR "Spinal Manipulation".ti OR "Continuous Passive Motion Therapy".ti OR "Soft Tissue Therapy".ti OR "Acupressure".ti OR "Massage".ti OR "Manual Lymphatic Drainage".ti OR "Myofascial Release Therapy".ti OR exp *"mobilization"/ OR "mobilization".ti OR "mobilisation".ti OR "mobilizing".ti OR "mobilising".ti OR "mobilize".ti OR "mobilise".ti OR "mobilized".ti OR "mobilised".ti OR "mobility".ti) AND **(exp "Aged"/ OR "elderly".af OR "elder".af OR "elders".af OR "geriatr*".af OR exp "Home for the Aged"/ OR exp "Elderly care"/ OR exp "Senior Center"/ OR "Nonagenarians".af OR "Nonagenarian".af OR "Octogenarians".af OR "Octogenarian".af OR "Centenarians".af OR "Centenarian".af OR "septuagenarian".af OR "septuagenarians".af OR "geront*".af OR "older person".af OR "old person".af OR "older patient".af OR "old patient".af OR "older persons".af OR "old persons".af OR "older patients".af OR "old patients".af OR "older women".af OR "old women".af OR "older men".af OR "old men".af OR "old adult".af OR "older adult".af OR "old adults".af OR "older adults".af OR "Older individual".af OR "Older individuals".af OR "old people".af OR "older people".af OR "Oldest Old".af OR "older population".af OR "aging population".af OR "aging population".af OR "old aged".af OR "old age".af OR (("older" ADJ3 "person") OR ("old" ADJ3 "person") OR ("older" ADJ3 "patient") OR ("old" ADJ3 "patient") OR ("older" ADJ3 "persons") OR ("old" ADJ3 "persons") OR ("older" ADJ3 "patients") OR ("old" ADJ3 "patients") OR ("older" ADJ3 "women") OR ("old" ADJ3 "women") OR ("older" ADJ3 "men") OR ("old" ADJ3 "men") OR ("old" ADJ3 "adult") OR ("older" ADJ3 "adult") OR ("old" ADJ3 "adults") OR ("older" ADJ3 "adults") OR ("Older" ADJ3 "individual") OR ("Older" ADJ3 "individuals") OR ("old" ADJ3 "people") OR ("older" ADJ3 "people") OR ("Oldest" ADJ3 "Old") OR ("older" ADJ3 "population") OR ("aging" ADJ3 "population") OR ("aging" ADJ3 "population") OR ("old" ADJ3 "aged") OR ("old" ADJ3 "age") OR (**"60" ADJ3 "year" ADJ3 "old") OR ("61" ADJ3 "year" ADJ3 "old") OR ("62" ADJ3 "year" ADJ3 "old") OR ("63" ADJ3 "year" ADJ3 "old") OR ("64" ADJ3 "year" ADJ3 "old") OR ("65" ADJ3 "year" ADJ3 "old") OR ("66" ADJ3 "year" ADJ3 "old") OR ("67" ADJ3 "year" ADJ3 "old") OR ("68" ADJ3 "year" ADJ3 "old") OR ("69" ADJ3 "year" ADJ3 "old") OR ("60" ADJ3 "years" ADJ3 "old") OR ("61" ADJ3 "years" ADJ3 "old") OR ("62" ADJ3 "years" ADJ3 "old") OR ("63" ADJ3 "years" ADJ3 "old") OR ("64" ADJ3 "years" ADJ3 "old") OR ("65" ADJ3 "years" ADJ3 "old") OR ("66" ADJ3 "years" ADJ3 "old") OR ("67" ADJ3 "years" ADJ3 "old") OR ("68" ADJ3 "years" ADJ3 "old") OR ("69" ADJ3 "years" ADJ3 "old") OR ("70" ADJ3 "year" ADJ3 "old") OR ("71" ADJ3 "year" ADJ3 "old") OR ("72" ADJ3 "year" ADJ3 "old") OR ("73" ADJ3 "year" ADJ3 "old") OR ("74" ADJ3 "year" ADJ3 "old") OR ("75" ADJ3 "year" ADJ3 "old") OR ("76" ADJ3 "year" ADJ3 "old") OR ("77" ADJ3 "year" ADJ3 "old") OR ("78" ADJ3 "year" ADJ3 "old") OR ("79" ADJ3 "year" ADJ3 "old") OR ("70" ADJ3 "years" ADJ3 "old") OR ("71" ADJ3 "years" ADJ3 "old") OR ("72" ADJ3 "years" ADJ3 "old") OR ("73" ADJ3 "years" ADJ3 "old") OR ("74" ADJ3 "years" ADJ3 "old") OR ("75" ADJ3 "years" ADJ3 "old") OR ("76" ADJ3 "years" ADJ3 "old") OR ("77" ADJ3 "years" ADJ3 "old") OR ("78" ADJ3 "years" ADJ3 "old") OR ("79" ADJ3 "years" ADJ3 "old") OR ("80" ADJ3 "year" ADJ3 "old") OR ("81" ADJ3 "year" ADJ3 "old") OR ("82" ADJ3 "year" ADJ3 "old") OR ("83" ADJ3 "year" ADJ3 "old") OR ("84" ADJ3 "year" ADJ3 "old") OR ("85" ADJ3 "year" ADJ3 "old") OR ("86" ADJ3 "year" ADJ3 "old") OR ("87" ADJ3 "year" ADJ3 "old") OR ("88" ADJ3 "year" ADJ3 "old") OR ("89" ADJ3 "year" ADJ3 "old") OR ("80" ADJ3 "years" ADJ3 "old") OR ("81" ADJ3 "years" ADJ3 "old") OR ("82" ADJ3 "years" ADJ3 "old") OR ("83" ADJ3 "years" ADJ3 "old") OR ("84" ADJ3 "years" ADJ3 "old") OR ("85" ADJ3 "years" ADJ3 "old") OR ("86" ADJ3 "years" ADJ3 "old") OR ("87" ADJ3 "years" ADJ3 "old") OR ("88" ADJ3 "years" ADJ3 "old") OR ("89" ADJ3 "years" ADJ3 "old") OR ("90" ADJ3 "year" ADJ3 "old") OR ("91" ADJ3 "year" ADJ3 "old") OR ("92" ADJ3 "year" ADJ3 "old") OR ("93" ADJ3 "year" ADJ3 "old") OR ("94" ADJ3 "year" ADJ3 "old") OR ("95" ADJ3 "year" ADJ3 "old") OR ("96" ADJ3 "year" ADJ3 "old") OR ("97" ADJ3 "year" ADJ3 "old") OR ("98" ADJ3 "year" ADJ3 "old") OR ("99" ADJ3 "year" ADJ3 "old") OR ("90" ADJ3 "years" ADJ3 "old") OR ("91" ADJ3 "years" ADJ3 "old") OR ("92" ADJ3 "years" ADJ3 "old") OR ("93" ADJ3 "years" ADJ3 "old") OR ("94" ADJ3 "years" ADJ3 "old") OR ("95" ADJ3 "years" ADJ3 "old") OR ("96" ADJ3 "years" ADJ3 "old") OR ("97" ADJ3 "years" ADJ3 "old") OR ("98" ADJ3 "years" ADJ3 "old") OR ("99" ADJ3 "years" ADJ3 "old") OR ("100" ADJ3 "year" ADJ3 "old") OR ("101" ADJ3 "year" ADJ3 "old") OR ("102" ADJ3 "year" ADJ3 "old") OR ("103" ADJ3 "year" ADJ3 "old") OR ("104" ADJ3 "year" ADJ3 "old") OR ("105" ADJ3 "year" ADJ3 "old") OR ("106" ADJ3 "year" ADJ3 "old") OR ("107" ADJ3 "year" ADJ3 "old") OR ("108" ADJ3 "year" ADJ3 "old") OR ("109" ADJ3 "year" ADJ3 "old") OR ("100" ADJ3 "years" ADJ3 "old") OR ("101" ADJ3 "years" ADJ3 "old") OR ("102" ADJ3 "years" ADJ3 "old") OR ("103" ADJ3 "years" ADJ3 "old") OR ("104" ADJ3 "years" ADJ3 "old") OR ("105" ADJ3 "years" ADJ3 "old") OR ("106" ADJ3 "years" ADJ3 "old") OR ("107" ADJ3 "years" ADJ3 "old") OR ("108" ADJ3 "years" ADJ3 "old") OR ("109" ADJ3 "years" ADJ3 "old"**)).ti,ab)) OR** ((exp *"Physical Activity"/ OR exp *"Exercise"/ OR "Exercise".ti,ab OR "Exercises".ti,ab OR "Exercising".ti,ab OR "Exercis*".ti,ab OR "physical activity".ti,ab OR "physical activities".ti,ab OR "Endurance Training".ti,ab OR "Exergaming".ti,ab OR "Gymnastics".ti,ab OR "Gymnastic".ti,ab OR "Interval Training".ti,ab OR "Jogging".ti,ab OR "Motor Activity".ti,ab OR "Movement".ti,ab OR "Muscle Stretching ".ti,ab OR "Physical Conditioning".ti,ab OR "Resistance Training".ti,ab OR "Running".ti,ab OR "Stair Climbing".ti,ab OR "Swimming".ti,ab OR "Training".ti,ab OR "Walking".ti,ab OR exp *"Kinesiotherapy"/ OR "Physical Exertion".ti,ab OR "Physical Exertions".ti,ab OR "Physical Effort".ti,ab OR "Physical Efforts".ti,ab OR exp *"Sport"/ OR "Sports".ti,ab OR "Sport".ti,ab OR "Athletic Performance".ti,ab OR "Baseball".ti,ab OR "Basketball".ti,ab OR "Bicycling".ti,ab OR "Boxing".ti,ab OR "Cardiorespiratory Fitness".ti,ab OR "Cardiorespiratory Fitness".ti,ab OR "Cricket Sport".ti,ab OR "Diving".ti,ab OR "Football".ti,ab OR "Golf".ti,ab OR "Gymnastics".ti,ab OR "Hockey".ti,ab OR "Jogging".ti,ab OR "Marathon Running".ti,ab OR "Martial Arts".ti,ab OR "Mountaineering".ti,ab OR "Nordic Walking".ti,ab OR "Physical Endurance".ti,ab OR "Physical Fitness".ti,ab OR "Racquet Sports".ti,ab OR "Return to Sport".ti,ab OR "Rugby".ti,ab OR "Running".ti,ab OR "Skating".ti,ab OR "Skiing".ti,ab OR "Snow Sports".ti,ab OR "Soccer".ti,ab OR "Swimming".ti,ab OR "Tai Ji".ti,ab OR "Team Sports".ti,ab OR "Tennis".ti,ab OR "Track and Field".ti,ab OR "Volleyball".ti,ab OR "Walking".ti,ab OR "Water Sports".ti,ab OR "Weight Lifting".ti,ab OR "Wrestling".ti,ab OR "Youth Sports".ti,ab OR "Qigong".ti,ab OR "Dance Therapy".ti,ab OR "Tai Ji".ti,ab OR "Yoga".ti,ab OR exp *"Physiotherapy"/ OR "Physical Therapy".ti,ab OR "physiotherapy".ti,ab OR "physiotherapy*".ti,ab OR exp *"Dry Needling"/ OR exp *"Electrotherapy"/ OR exp *"Shock wave Therapy"/ OR exp *"Balneotherapy"/ OR exp *"Musculoskeletal Manipulation"/ OR "Dry Needling".ti,ab OR "Electric Stimulation Therapy".ti,ab OR "Electroacupuncture".ti,ab OR "Pulsed Radiofrequency Treatment".ti,ab OR "Spinal Cord Stimulation".ti,ab OR "Transcutaneous Electric Nerve Stimulation".ti,ab OR "Extracorporeal Shockwave Therapy".ti,ab OR "Hydrotherapy".ti,ab OR "Aquatic Therapy".ti,ab OR "Therapeutic Irrigation".ti,ab OR "Musculoskeletal Manipulations".ti,ab OR "Applied Kinesiology".ti,ab OR "Orthopedic Manipulation".ti,ab OR "Orthopaedic Manipulation".ti,ab OR "Osteopathic Manipulation".ti,ab OR "Spinal Manipulation".ti,ab OR "Continuous Passive Motion Therapy".ti,ab OR "Soft Tissue Therapy".ti,ab OR "Acupressure".ti,ab OR "Massage".ti,ab OR "Manual Lymphatic Drainage".ti,ab OR "Myofascial Release Therapy".ti,ab OR exp *"mobilization"/ OR "mobilization".ti,ab OR "mobilisation".ti,ab OR "mobilizing".ti,ab OR "mobilising".ti,ab OR "mobilize".ti,ab OR "mobilise".ti,ab OR "mobilized".ti,ab OR "mobilised".ti,ab OR "mobility".ti,ab) AND (exp *"Ankle Fracture"/ OR exp *"Femur Fracture"/ OR exp *"Fibula Fracture"/ OR exp *"Knee Fracture"/ OR exp *"Tibia Fracture"/ OR "Ankle Fractures".ti OR "Femoral Fractures".ti OR "Hip Fractures".ti OR "Femoral Neck Fractures".ti OR "Proximal Femoral Fractures".ti OR "Hoffa Fracture".ti OR "Proximal Femoral Fractures".ti OR "Fibula Fractures".ti OR "Knee Fractures".ti OR "Hoffa Fracture".ti OR "Patella Fracture".ti OR "Tibial Plateau Fractures".ti OR "Tibial Fractures".ti OR "Tibial Plateau Fractures".ti OR "Ankle Fracture".ti OR "Femoral Fracture".ti OR "Hip Fracture".ti OR "Femoral Neck Fracture".ti OR "Proximal Femoral Fracture".ti OR "Hoffa Fractures".ti OR "Proximal Femoral Fracture".ti OR "Fibula Fracture".ti OR "Knee Fracture".ti OR "Hoffa Fractures".ti OR "Patella Fractures".ti OR "Tibial Plateau Fracture".ti OR "Tibial Fracture".ti OR "Tibial Plateau Fracture".ti OR ((exp *"Lower Limb"/ OR "lower limb".ti OR "lower limbs".ti OR "lower extremity".ti OR "lower extremities".ti OR "Ankle".ti OR "Foot".ti OR "Heel".ti OR "Hip".ti OR "Knee".ti OR "Leg".ti OR "Ankles".ti OR "Feet".ti OR "Heels".ti OR "Hips".ti OR "Knees".ti OR "Legs".ti) AND (exp *"Fracture"/ OR "fracture".ti OR "fractures".ti OR "fractur*".ti)) OR **"acutely ill".ti**) AND (exp *"immobility"/ OR "immobility".ti,ab OR "immobile".ti,ab OR "immobil*".ti,ab OR "temporary".ti,ab OR exp *"non weight bearing"/ OR "non weight bearing".ti,ab OR "nonweight bearing".ti,ab OR "non weight bear*".ti,ab OR "nonweight bear*".ti,ab OR "physically dependent".ti,ab OR "physically disabled".ti,ab OR **"physically dependent".ti,ab OR "physically disabled".ti,ab OR "disuse".ti,ab OR exp *"bed rest"/ OR "bed rest".ti,ab OR "bedrest".ti,ab OR "leg immobilization".ti,ab OR "leg immobilisation".ti,ab OR exp *"Weightlessness"/ OR exp *"deconditioning"/ OR "deconditioning".ti,ab OR "permissive weight-bearing".ti,ab**)**) OR** ((exp *"Physical Activity"/ OR exp *"Exercise"/ OR "Exercise".ti OR "Exercises".ti OR "Exercising".ti OR "Exercis*".ti OR "physical activity".ti OR "physical activities".ti OR "Endurance Training".ti OR "Exergaming".ti OR "Gymnastics".ti OR "Gymnastic".ti OR "Interval Training".ti OR "Jogging".ti OR "Motor Activity".ti OR "Movement".ti OR "Muscle Stretching ".ti OR "Physical Conditioning".ti OR "Resistance Training".ti OR "Running".ti OR "Stair Climbing".ti OR "Swimming".ti OR "Training".ti OR "Walking".ti OR exp *"Kinesiotherapy"/ OR "Physical Exertion".ti OR "Physical Exertions".ti OR "Physical Effort".ti OR "Physical Efforts".ti OR exp *"Sport"/ OR "Sports".ti OR "Sport".ti OR "Athletic Performance".ti OR "Baseball".ti OR "Basketball".ti OR "Bicycling".ti OR "Boxing".ti OR "Cardiorespiratory Fitness".ti OR "Cardiorespiratory Fitness".ti OR "Cricket Sport".ti OR "Diving".ti OR "Football".ti OR "Golf".ti OR "Gymnastics".ti OR "Hockey".ti OR "Jogging".ti OR "Marathon Running".ti OR "Martial Arts".ti OR "Mountaineering".ti OR "Nordic Walking".ti OR "Physical Endurance".ti OR "Physical Fitness".ti OR "Racquet Sports".ti OR "Return to Sport".ti OR "Rugby".ti OR "Running".ti OR "Skating".ti OR "Skiing".ti OR "Snow Sports".ti OR "Soccer".ti OR "Swimming".ti OR "Tai Ji".ti OR "Team Sports".ti OR "Tennis".ti OR "Track and Field".ti OR "Volleyball".ti OR "Walking".ti OR "Water Sports".ti OR "Weight Lifting".ti OR "Wrestling".ti OR "Youth Sports".ti OR "Qigong".ti OR "Dance Therapy".ti OR "Tai Ji".ti OR "Yoga".ti OR exp *"Physiotherapy"/ OR "Physical Therapy".ti OR "physiotherapy".ti OR "physiotherapy*".ti OR exp *"Dry Needling"/ OR exp *"Electrotherapy"/ OR exp *"Shock wave Therapy"/ OR exp *"Balneotherapy"/ OR exp *"Musculoskeletal Manipulation"/ OR "Dry Needling".ti OR "Electric Stimulation Therapy".ti OR "Electroacupuncture".ti OR "Pulsed Radiofrequency Treatment".ti OR "Spinal Cord Stimulation".ti OR "Transcutaneous Electric Nerve Stimulation".ti OR "Extracorporeal Shockwave Therapy".ti OR "Hydrotherapy".ti OR "Aquatic Therapy".ti OR "Therapeutic Irrigation".ti OR "Musculoskeletal Manipulations".ti OR "Applied Kinesiology".ti OR "Orthopedic Manipulation".ti OR "Orthopaedic Manipulation".ti OR "Osteopathic Manipulation".ti OR "Spinal Manipulation".ti OR "Continuous Passive Motion Therapy".ti OR "Soft Tissue Therapy".ti OR "Acupressure".ti OR "Massage".ti OR "Manual Lymphatic Drainage".ti OR "Myofascial Release Therapy".ti OR exp *"mobilization"/ OR "mobilization".ti OR "mobilisation".ti OR "mobilizing".ti OR "mobilising".ti OR "mobilize".ti OR "mobilise".ti OR "mobilized".ti OR "mobilised".ti OR "mobility".ti) AND (**"disuse".ti,ab**) AND **(exp "Aged"/ OR "elderly".af OR "elder".af OR "elders".af OR "geriatr*".af OR exp "Home for the Aged"/ OR exp "Elderly care"/ OR exp "Senior Center"/ OR "Nonagenarians".af OR "Nonagenarian".af OR "Octogenarians".af OR "Octogenarian".af OR "Centenarians".af OR "Centenarian".af OR "septuagenarian".af OR "septuagenarians".af OR "geront*".af OR "older person".af OR "old person".af OR "older patient".af OR "old patient".af OR "older persons".af OR "old persons".af OR "older patients".af OR "old patients".af OR "older women".af OR "old women".af OR "older men".af OR "old men".af OR "old adult".af OR "older adult".af OR "old adults".af OR "older adults".af OR "Older individual".af OR "Older individuals".af OR "old people".af OR "older people".af OR "Oldest Old".af OR "older population".af OR "aging population".af OR "aging population".af OR "old aged".af OR "old age".af OR (("older" ADJ3 "person") OR ("old" ADJ3 "person") OR ("older" ADJ3 "patient") OR ("old" ADJ3 "patient") OR ("older" ADJ3 "persons") OR ("old" ADJ3 "persons") OR ("older" ADJ3 "patients") OR ("old" ADJ3 "patients") OR ("older" ADJ3 "women") OR ("old" ADJ3 "women") OR ("older" ADJ3 "men") OR ("old" ADJ3 "men") OR ("old" ADJ3 "adult") OR ("older" ADJ3 "adult") OR ("old" ADJ3 "adults") OR ("older" ADJ3 "adults") OR ("Older" ADJ3 "individual") OR ("Older" ADJ3 "individuals") OR ("old" ADJ3 "people") OR ("older" ADJ3 "people") OR ("Oldest" ADJ3 "Old") OR ("older" ADJ3 "population") OR ("aging" ADJ3 "population") OR ("aging" ADJ3 "population") OR ("old" ADJ3 "aged") OR ("old" ADJ3 "age") OR (**"60" ADJ3 "year" ADJ3 "old") OR ("61" ADJ3 "year" ADJ3 "old") OR ("62" ADJ3 "year" ADJ3 "old") OR ("63" ADJ3 "year" ADJ3 "old") OR ("64" ADJ3 "year" ADJ3 "old") OR ("65" ADJ3 "year" ADJ3 "old") OR ("66" ADJ3 "year" ADJ3 "old") OR ("67" ADJ3 "year" ADJ3 "old") OR ("68" ADJ3 "year" ADJ3 "old") OR ("69" ADJ3 "year" ADJ3 "old") OR ("60" ADJ3 "years" ADJ3 "old") OR ("61" ADJ3 "years" ADJ3 "old") OR ("62" ADJ3 "years" ADJ3 "old") OR ("63" ADJ3 "years" ADJ3 "old") OR ("64" ADJ3 "years" ADJ3 "old") OR ("65" ADJ3 "years" ADJ3 "old") OR ("66" ADJ3 "years" ADJ3 "old") OR ("67" ADJ3 "years" ADJ3 "old") OR ("68" ADJ3 "years" ADJ3 "old") OR ("69" ADJ3 "years" ADJ3 "old") OR ("70" ADJ3 "year" ADJ3 "old") OR ("71" ADJ3 "year" ADJ3 "old") OR ("72" ADJ3 "year" ADJ3 "old") OR ("73" ADJ3 "year" ADJ3 "old") OR ("74" ADJ3 "year" ADJ3 "old") OR ("75" ADJ3 "year" ADJ3 "old") OR ("76" ADJ3 "year" ADJ3 "old") OR ("77" ADJ3 "year" ADJ3 "old") OR ("78" ADJ3 "year" ADJ3 "old") OR ("79" ADJ3 "year" ADJ3 "old") OR ("70" ADJ3 "years" ADJ3 "old") OR ("71" ADJ3 "years" ADJ3 "old") OR ("72" ADJ3 "years" ADJ3 "old") OR ("73" ADJ3 "years" ADJ3 "old") OR ("74" ADJ3 "years" ADJ3 "old") OR ("75" ADJ3 "years" ADJ3 "old") OR ("76" ADJ3 "years" ADJ3 "old") OR ("77" ADJ3 "years" ADJ3 "old") OR ("78" ADJ3 "years" ADJ3 "old") OR ("79" ADJ3 "years" ADJ3 "old") OR ("80" ADJ3 "year" ADJ3 "old") OR ("81" ADJ3 "year" ADJ3 "old") OR ("82" ADJ3 "year" ADJ3 "old") OR ("83" ADJ3 "year" ADJ3 "old") OR ("84" ADJ3 "year" ADJ3 "old") OR ("85" ADJ3 "year" ADJ3 "old") OR ("86" ADJ3 "year" ADJ3 "old") OR ("87" ADJ3 "year" ADJ3 "old") OR ("88" ADJ3 "year" ADJ3 "old") OR ("89" ADJ3 "year" ADJ3 "old") OR ("80" ADJ3 "years" ADJ3 "old") OR ("81" ADJ3 "years" ADJ3 "old") OR ("82" ADJ3 "years" ADJ3 "old") OR ("83" ADJ3 "years" ADJ3 "old") OR ("84" ADJ3 "years" ADJ3 "old") OR ("85" ADJ3 "years" ADJ3 "old") OR ("86" ADJ3 "years" ADJ3 "old") OR ("87" ADJ3 "years" ADJ3 "old") OR ("88" ADJ3 "years" ADJ3 "old") OR ("89" ADJ3 "years" ADJ3 "old") OR ("90" ADJ3 "year" ADJ3 "old") OR ("91" ADJ3 "year" ADJ3 "old") OR ("92" ADJ3 "year" ADJ3 "old") OR ("93" ADJ3 "year" ADJ3 "old") OR ("94" ADJ3 "year" ADJ3 "old") OR ("95" ADJ3 "year" ADJ3 "old") OR ("96" ADJ3 "year" ADJ3 "old") OR ("97" ADJ3 "year" ADJ3 "old") OR ("98" ADJ3 "year" ADJ3 "old") OR ("99" ADJ3 "year" ADJ3 "old") OR ("90" ADJ3 "years" ADJ3 "old") OR ("91" ADJ3 "years" ADJ3 "old") OR ("92" ADJ3 "years" ADJ3 "old") OR ("93" ADJ3 "years" ADJ3 "old") OR ("94" ADJ3 "years" ADJ3 "old") OR ("95" ADJ3 "years" ADJ3 "old") OR ("96" ADJ3 "years" ADJ3 "old") OR ("97" ADJ3 "years" ADJ3 "old") OR ("98" ADJ3 "years" ADJ3 "old") OR ("99" ADJ3 "years" ADJ3 "old") OR ("100" ADJ3 "year" ADJ3 "old") OR ("101" ADJ3 "year" ADJ3 "old") OR ("102" ADJ3 "year" ADJ3 "old") OR ("103" ADJ3 "year" ADJ3 "old") OR ("104" ADJ3 "year" ADJ3 "old") OR ("105" ADJ3 "year" ADJ3 "old") OR ("106" ADJ3 "year" ADJ3 "old") OR ("107" ADJ3 "year" ADJ3 "old") OR ("108" ADJ3 "year" ADJ3 "old") OR ("109" ADJ3 "year" ADJ3 "old") OR ("100" ADJ3 "years" ADJ3 "old") OR ("101" ADJ3 "years" ADJ3 "old") OR ("102" ADJ3 "years" ADJ3 "old") OR ("103" ADJ3 "years" ADJ3 "old") OR ("104" ADJ3 "years" ADJ3 "old") OR ("105" ADJ3 "years" ADJ3 "old") OR ("106" ADJ3 "years" ADJ3 "old") OR ("107" ADJ3 "years" ADJ3 "old") OR ("108" ADJ3 "years" ADJ3 "old") OR ("109" ADJ3 "years" ADJ3 "old"**)).ti,ab))) NOT (exp "Animals"/ NOT exp "Humans"/)** NOT (("Case Report"/ OR "case report".ti OR "case report".af OR "Review"/ OR "review".ti) NOT ("Clinical Study"/ OR exp "Clinical Trial"/ OR "trial".ti OR "RCT".ti OR exp "systematic review"/ OR "systematic review".ti)) AND english.la

**Academic Search Premier**

Limit to Academic Journals.

Limit to English.

**(**((TI("Wheelchair" OR "Wheelchairs" OR "Wheelchair" OR "Wheelchair*" OR "Wheel chairs" OR "Wheel chair" OR "Wheel chair*" OR "Mobility Scooter" OR "Mobility Scooters") OR SU("Wheelchair" OR "Wheelchairs" OR "Wheelchair" OR "Wheelchair*" OR "Wheel chairs" OR "Wheel chair" OR "Wheel chair*" OR "Mobility Scooter" OR "Mobility Scooters") OR KW("Wheelchair" OR "Wheelchairs" OR "Wheelchair" OR "Wheelchair*" OR "Wheel chairs" OR "Wheel chair" OR "Wheel chair*" OR "Mobility Scooter" OR "Mobility Scooters") OR AB("Wheelchair" OR "Wheelchairs" OR "Wheelchair" OR "Wheelchair*" OR "Wheel chairs" OR "Wheel chair" OR "Wheel chair*" OR "Mobility Scooter" OR "Mobility Scooters")) AND (TI("Physical Activity" OR "Exercise" OR "Exercise" OR "Exercises" OR "Exercising" OR "Exercis*" OR "physical activity" OR "physical activities" OR "Endurance Training" OR "Exergaming" OR "Gymnastics" OR "Gymnastic" OR "Interval Training" OR "Jogging" OR "Motor Activity" OR "Movement" OR "Muscle Stretching" OR "Physical Conditioning" OR "Resistance Training" OR "Running" OR "Stair Climbing" OR "Swimming" OR "Training" OR "Walking" OR "Kinesiotherapy" OR "Physical Exertion" OR "Physical Exertions" OR "Physical Effort" OR "Physical Efforts" OR "Sport" OR "Sports" OR "Sport" OR "Athletic Performance" OR "Baseball" OR "Basketball" OR "Bicycling" OR "Boxing" OR "Cardiorespiratory Fitness" OR "Cardiorespiratory Fitness" OR "Cricket Sport" OR "Diving" OR "Football" OR "Golf" OR "Gymnastics" OR "Hockey" OR "Jogging" OR "Marathon Running" OR "Martial Arts" OR "Mountaineering" OR "Nordic Walking" OR "Physical Endurance" OR "Physical Fitness" OR "Racquet Sports" OR "Return to Sport" OR "Rugby" OR "Running" OR "Skating" OR "Skiing" OR "Snow Sports" OR "Soccer" OR "Swimming" OR "Tai Ji" OR "Team Sports" OR "Tennis" OR "Track and Field" OR "Volleyball" OR "Walking" OR "Water Sports" OR "Weight Lifting" OR "Wrestling" OR "Youth Sports" OR "Qigong" OR "Dance Therapy" OR "Tai Ji" OR "Yoga" OR "Physiotherapy" OR "Physical Therapy" OR "physiotherapy" OR "physiotherapy*" OR "Dry Needling" OR "Electrotherapy" OR "Shock wave Therapy" OR "Balneotherapy" OR "Musculoskeletal Manipulation" OR "Dry Needling" OR "Electric Stimulation Therapy" OR "Electroacupuncture" OR "Pulsed Radiofrequency Treatment" OR "Spinal Cord Stimulation" OR "Transcutaneous Electric Nerve Stimulation" OR "Extracorporeal Shockwave Therapy" OR "Hydrotherapy" OR "Aquatic Therapy" OR "Therapeutic Irrigation" OR "Musculoskeletal Manipulations" OR "Applied Kinesiology" OR "Orthopedic Manipulation" OR "Orthopaedic Manipulation" OR "Osteopathic Manipulation" OR "Spinal Manipulation" OR "Continuous Passive Motion Therapy" OR "Soft Tissue Therapy" OR "Acupressure" OR "Massage" OR "Manual Lymphatic Drainage" OR "Myofascial Release Therapy" OR "mobilization" OR "mobilization" OR "mobilisation" OR "mobilizing" OR "mobilising" OR "mobilize" OR "mobilise" OR "mobilized" OR "mobilised" OR "mobility") OR SU("Physical Activity" OR "Exercise" OR "Exercise" OR "Exercises" OR "Exercising" OR "Exercis*" OR "physical activity" OR "physical activities" OR "Endurance Training" OR "Exergaming" OR "Gymnastics" OR "Gymnastic" OR "Interval Training" OR "Jogging" OR "Motor Activity" OR "Movement" OR "Muscle Stretching"OR "Physical Conditioning" OR "Resistance Training" OR "Running" OR "Stair Climbing" OR "Swimming" OR "Training" OR "Walking" OR "Kinesiotherapy" OR "Physical Exertion" OR "Physical Exertions" OR "Physical Effort" OR "Physical Efforts" OR "Sport" OR "Sports" OR "Sport" OR "Athletic Performance" OR "Baseball" OR "Basketball" OR "Bicycling" OR "Boxing" OR "Cardiorespiratory Fitness" OR "Cardiorespiratory Fitness" OR "Cricket Sport" OR "Diving" OR "Football" OR "Golf" OR "Gymnastics" OR "Hockey" OR "Jogging" OR "Marathon Running" OR "Martial Arts" OR "Mountaineering" OR "Nordic Walking" OR "Physical Endurance" OR "Physical Fitness" OR "Racquet Sports" OR "Return to Sport" OR "Rugby" OR "Running" OR "Skating" OR "Skiing" OR "Snow Sports" OR "Soccer" OR "Swimming" OR "Tai Ji" OR "Team Sports" OR "Tennis" OR "Track and Field" OR "Volleyball" OR "Walking" OR "Water Sports" OR "Weight Lifting" OR "Wrestling" OR "Youth Sports" OR "Qigong" OR "Dance Therapy" OR "Tai Ji" OR "Yoga" OR "Physiotherapy" OR "Physical Therapy" OR "physiotherapy" OR "physiotherapy*" OR "Dry Needling" OR "Electrotherapy" OR "Shock wave Therapy" OR "Balneotherapy" OR "Musculoskeletal Manipulation" OR "Dry Needling" OR "Electric Stimulation Therapy" OR "Electroacupuncture" OR "Pulsed Radiofrequency Treatment" OR "Spinal Cord Stimulation" OR "Transcutaneous Electric Nerve Stimulation" OR "Extracorporeal Shockwave Therapy" OR "Hydrotherapy" OR "Aquatic Therapy" OR "Therapeutic Irrigation" OR "Musculoskeletal Manipulations" OR "Applied Kinesiology" OR "Orthopedic Manipulation" OR "Orthopaedic Manipulation" OR "Osteopathic Manipulation" OR "Spinal Manipulation" OR "Continuous Passive Motion Therapy" OR "Soft Tissue Therapy" OR "Acupressure" OR "Massage" OR "Manual Lymphatic Drainage" OR "Myofascial Release Therapy" OR "mobilization" OR "mobilization" OR "mobilisation" OR "mobilizing" OR "mobilising" OR "mobilize" OR "mobilise" OR "mobilized" OR "mobilised" OR "mobility") OR KW("Physical Activity" OR "Exercise" OR "Exercise" OR "Exercises" OR "Exercising" OR "Exercis*" OR "physical activity" OR "physical activities" OR "Endurance Training" OR "Exergaming" OR "Gymnastics" OR "Gymnastic" OR "Interval Training" OR "Jogging" OR "Motor Activity" OR "Movement" OR "Muscle Stretching"OR "Physical Conditioning" OR "Resistance Training" OR "Running" OR "Stair Climbing" OR "Swimming" OR "Training" OR "Walking" OR "Kinesiotherapy" OR "Physical Exertion" OR "Physical Exertions" OR "Physical Effort" OR "Physical Efforts" OR "Sport" OR "Sports" OR "Sport" OR "Athletic Performance" OR "Baseball" OR "Basketball" OR "Bicycling" OR "Boxing" OR "Cardiorespiratory Fitness" OR "Cardiorespiratory Fitness" OR "Cricket Sport" OR "Diving" OR "Football" OR "Golf" OR "Gymnastics" OR "Hockey" OR "Jogging" OR "Marathon Running" OR "Martial Arts" OR "Mountaineering" OR "Nordic Walking" OR "Physical Endurance" OR "Physical Fitness" OR "Racquet Sports" OR "Return to Sport" OR "Rugby" OR "Running" OR "Skating" OR "Skiing" OR "Snow Sports" OR "Soccer" OR "Swimming" OR "Tai Ji" OR "Team Sports" OR "Tennis" OR "Track and Field" OR "Volleyball" OR "Walking" OR "Water Sports" OR "Weight Lifting" OR "Wrestling" OR "Youth Sports" OR "Qigong" OR "Dance Therapy" OR "Tai Ji" OR "Yoga" OR "Physiotherapy" OR "Physical Therapy" OR "physiotherapy" OR "physiotherapy*" OR "Dry Needling" OR "Electrotherapy" OR "Shock wave Therapy" OR "Balneotherapy" OR "Musculoskeletal Manipulation" OR "Dry Needling" OR "Electric Stimulation Therapy" OR "Electroacupuncture" OR "Pulsed Radiofrequency Treatment" OR "Spinal Cord Stimulation" OR "Transcutaneous Electric Nerve Stimulation" OR "Extracorporeal Shockwave Therapy" OR "Hydrotherapy" OR "Aquatic Therapy" OR "Therapeutic Irrigation" OR "Musculoskeletal Manipulations" OR "Applied Kinesiology" OR "Orthopedic Manipulation" OR "Orthopaedic Manipulation" OR "Osteopathic Manipulation" OR "Spinal Manipulation" OR "Continuous Passive Motion Therapy" OR "Soft Tissue Therapy" OR "Acupressure" OR "Massage" OR "Manual Lymphatic Drainage" OR "Myofascial Release Therapy" OR "mobilization" OR "mobilization" OR "mobilisation" OR "mobilizing" OR "mobilising" OR "mobilize" OR "mobilise" OR "mobilized" OR "mobilised" OR "mobility") OR AB("Physical Activity" OR "Exercise" OR "Exercise" OR "Exercises" OR "Exercising" OR "Exercis*" OR "physical activity" OR "physical activities" OR "Endurance Training" OR "Exergaming" OR "Gymnastics" OR "Gymnastic" OR "Interval Training" OR "Jogging" OR "Motor Activity" OR "Movement" OR "Muscle Stretching"OR "Physical Conditioning" OR "Resistance Training" OR "Running" OR "Stair Climbing" OR "Swimming" OR "Training" OR "Walking" OR "Kinesiotherapy" OR "Physical Exertion" OR "Physical Exertions" OR "Physical Effort" OR "Physical Efforts" OR "Sport" OR "Sports" OR "Sport" OR "Athletic Performance" OR "Baseball" OR "Basketball" OR "Bicycling" OR "Boxing" OR "Cardiorespiratory Fitness" OR "Cardiorespiratory Fitness" OR "Cricket Sport" OR "Diving" OR "Football" OR "Golf" OR "Gymnastics" OR "Hockey" OR "Jogging" OR "Marathon Running" OR "Martial Arts" OR "Mountaineering" OR "Nordic Walking" OR "Physical Endurance" OR "Physical Fitness" OR "Racquet Sports" OR "Return to Sport" OR "Rugby" OR "Running" OR "Skating" OR "Skiing" OR "Snow Sports" OR "Soccer" OR "Swimming" OR "Tai Ji" OR "Team Sports" OR "Tennis" OR "Track and Field" OR "Volleyball" OR "Walking" OR "Water Sports" OR "Weight Lifting" OR "Wrestling" OR "Youth Sports" OR "Qigong" OR "Dance Therapy" OR "Tai Ji" OR "Yoga" OR "Physiotherapy" OR "Physical Therapy" OR "physiotherapy" OR "physiotherapy*" OR "Dry Needling" OR "Electrotherapy" OR "Shock wave Therapy" OR "Balneotherapy" OR "Musculoskeletal Manipulation" OR "Dry Needling" OR "Electric Stimulation Therapy" OR "Electroacupuncture" OR "Pulsed Radiofrequency Treatment" OR "Spinal Cord Stimulation" OR "Transcutaneous Electric Nerve Stimulation" OR "Extracorporeal Shockwave Therapy" OR "Hydrotherapy" OR "Aquatic Therapy" OR "Therapeutic Irrigation" OR "Musculoskeletal Manipulations" OR "Applied Kinesiology" OR "Orthopedic Manipulation" OR "Orthopaedic Manipulation" OR "Osteopathic Manipulation" OR "Spinal Manipulation" OR "Continuous Passive Motion Therapy" OR "Soft Tissue Therapy" OR "Acupressure" OR "Massage" OR "Manual Lymphatic Drainage" OR "Myofascial Release Therapy" OR "mobilization" OR "mobilization" OR "mobilisation" OR "mobilizing" OR "mobilising" OR "mobilize" OR "mobilise" OR "mobilized" OR "mobilised" OR "mobility")) AND (TI("Ankle Fracture" OR "Femur Fracture" OR "Fibula Fracture" OR "Knee Fracture" OR "Tibia Fracture" OR "Ankle Fractures" OR "Femoral Fractures" OR "Hip Fractures" OR "Femoral Neck Fractures" OR "Proximal Femoral Fractures" OR "Hoffa Fracture" OR "Proximal Femoral Fractures" OR "Fibula Fractures" OR "Knee Fractures" OR "Hoffa Fracture" OR "Patella Fracture" OR "Tibial Plateau Fractures" OR "Tibial Fractures" OR "Tibial Plateau Fractures" OR "Ankle Fracture" OR "Femoral Fracture" OR "Hip Fracture" OR "Femoral Neck Fracture" OR "Proximal Femoral Fracture" OR "Hoffa Fractures" OR "Proximal Femoral Fracture" OR "Fibula Fracture" OR "Knee Fracture" OR "Hoffa Fractures" OR "Patella Fractures" OR "Tibial Plateau Fracture" OR "Tibial Fracture" OR "Tibial Plateau Fracture" OR (("Lower Limb" OR "lower limb" OR "lower limbs" OR "lower extremity" OR "lower extremities" OR "Ankle" OR "Foot" OR "Heel" OR "Hip" OR "Knee" OR "Leg" OR "Ankles" OR "Feet" OR "Heels" OR "Hips" OR "Knees" OR "Legs") AND ("Fracture" OR "fracture" OR "fractures" OR "fractur*")) OR "immobility" OR "immobility" OR "immobile" OR "immobil*" OR "temporary" OR "non weight bearing" OR "non weight bearing" OR "nonweight bearing" OR "non weight bear*" OR "nonweight bear*") OR SU("Ankle Fracture" OR "Femur Fracture" OR "Fibula Fracture" OR "Knee Fracture" OR "Tibia Fracture" OR "Ankle Fractures" OR "Femoral Fractures" OR "Hip Fractures" OR "Femoral Neck Fractures" OR "Proximal Femoral Fractures" OR "Hoffa Fracture" OR "Proximal Femoral Fractures" OR "Fibula Fractures" OR "Knee Fractures" OR "Hoffa Fracture" OR "Patella Fracture" OR "Tibial Plateau Fractures" OR "Tibial Fractures" OR "Tibial Plateau Fractures" OR "Ankle Fracture" OR "Femoral Fracture" OR "Hip Fracture" OR "Femoral Neck Fracture" OR "Proximal Femoral Fracture" OR "Hoffa Fractures" OR "Proximal Femoral Fracture" OR "Fibula Fracture" OR "Knee Fracture" OR "Hoffa Fractures" OR "Patella Fractures" OR "Tibial Plateau Fracture" OR "Tibial Fracture" OR "Tibial Plateau Fracture" OR (("Lower Limb" OR "lower limb" OR "lower limbs" OR "lower extremity" OR "lower extremities" OR "Ankle" OR "Foot" OR "Heel" OR "Hip" OR "Knee" OR "Leg" OR "Ankles" OR "Feet" OR "Heels" OR "Hips" OR "Knees" OR "Legs") AND ("Fracture" OR "fracture" OR "fractures" OR "fractur*")) OR "immobility" OR "immobility" OR "immobile" OR "immobil*" OR "temporary" OR "non weight bearing" OR "non weight bearing" OR "nonweight bearing" OR "non weight bear*" OR "nonweight bear*") OR KW("Ankle Fracture" OR "Femur Fracture" OR "Fibula Fracture" OR "Knee Fracture" OR "Tibia Fracture" OR "Ankle Fractures" OR "Femoral Fractures" OR "Hip Fractures" OR "Femoral Neck Fractures" OR "Proximal Femoral Fractures" OR "Hoffa Fracture" OR "Proximal Femoral Fractures" OR "Fibula Fractures" OR "Knee Fractures" OR "Hoffa Fracture" OR "Patella Fracture" OR "Tibial Plateau Fractures" OR "Tibial Fractures" OR "Tibial Plateau Fractures" OR "Ankle Fracture" OR "Femoral Fracture" OR "Hip Fracture" OR "Femoral Neck Fracture" OR "Proximal Femoral Fracture" OR "Hoffa Fractures" OR "Proximal Femoral Fracture" OR "Fibula Fracture" OR "Knee Fracture" OR "Hoffa Fractures" OR "Patella Fractures" OR "Tibial Plateau Fracture" OR "Tibial Fracture" OR "Tibial Plateau Fracture" OR (("Lower Limb" OR "lower limb" OR "lower limbs" OR "lower extremity" OR "lower extremities" OR "Ankle" OR "Foot" OR "Heel" OR "Hip" OR "Knee" OR "Leg" OR "Ankles" OR "Feet" OR "Heels" OR "Hips" OR "Knees" OR "Legs") AND ("Fracture" OR "fracture" OR "fractures" OR "fractur*")) OR "immobility" OR "immobility" OR "immobile" OR "immobil*" OR "temporary" OR "non weight bearing" OR "non weight bearing" OR "nonweight bearing" OR "non weight bear*" OR "nonweight bear*") OR AB("Ankle Fracture" OR "Femur Fracture" OR "Fibula Fracture" OR "Knee Fracture" OR "Tibia Fracture" OR "Ankle Fractures" OR "Femoral Fractures" OR "Hip Fractures" OR "Femoral Neck Fractures" OR "Proximal Femoral Fractures" OR "Hoffa Fracture" OR "Proximal Femoral Fractures" OR "Fibula Fractures" OR "Knee Fractures" OR "Hoffa Fracture" OR "Patella Fracture" OR "Tibial Plateau Fractures" OR "Tibial Fractures" OR "Tibial Plateau Fractures" OR "Ankle Fracture" OR "Femoral Fracture" OR "Hip Fracture" OR "Femoral Neck Fracture" OR "Proximal Femoral Fracture" OR "Hoffa Fractures" OR "Proximal Femoral Fracture" OR "Fibula Fracture" OR "Knee Fracture" OR "Hoffa Fractures" OR "Patella Fractures" OR "Tibial Plateau Fracture" OR "Tibial Fracture" OR "Tibial Plateau Fracture" OR (("Lower Limb" OR "lower limb" OR "lower limbs" OR "lower extremity" OR "lower extremities" OR "Ankle" OR "Foot" OR "Heel" OR "Hip" OR "Knee" OR "Leg" OR "Ankles" OR "Feet" OR "Heels" OR "Hips" OR "Knees" OR "Legs") AND ("Fracture" OR "fracture" OR "fractures" OR "fractur*")) OR "immobility" OR "immobility" OR "immobile" OR "immobil*" OR "temporary" OR "non weight bearing" OR "non weight bearing" OR "nonweight bearing" OR "non weight bear*" OR "nonweight bear*"))**) OR** (TI("Wheelchair" OR "Wheelchairs" OR "Wheelchair" OR "Wheelchair*" OR "Wheel chairs" OR "Wheel chair" OR "Wheel chair*" OR "Mobility Scooter" OR "Mobility Scooters") AND TI("Physical Activity" OR "Exercise" OR "Exercise" OR "Exercises" OR "Exercising" OR "Exercis*" OR "physical activity" OR "physical activities" OR "Endurance Training" OR "Exergaming" OR "Gymnastics" OR "Gymnastic" OR "Interval Training" OR "Jogging" OR "Motor Activity" OR "Movement" OR "Muscle Stretching"OR "Physical Conditioning" OR "Resistance Training" OR "Running" OR "Stair Climbing" OR "Swimming" OR "Training" OR "Walking" OR "Kinesiotherapy" OR "Physical Exertion" OR "Physical Exertions" OR "Physical Effort" OR "Physical Efforts" OR "Sport" OR "Sports" OR "Sport" OR "Athletic Performance" OR "Baseball" OR "Basketball" OR "Bicycling" OR "Boxing" OR "Cardiorespiratory Fitness" OR "Cardiorespiratory Fitness" OR "Cricket Sport" OR "Diving" OR "Football" OR "Golf" OR "Gymnastics" OR "Hockey" OR "Jogging" OR "Marathon Running" OR "Martial Arts" OR "Mountaineering" OR "Nordic Walking" OR "Physical Endurance" OR "Physical Fitness" OR "Racquet Sports" OR "Return to Sport" OR "Rugby" OR "Running" OR "Skating" OR "Skiing" OR "Snow Sports" OR "Soccer" OR "Swimming" OR "Tai Ji" OR "Team Sports" OR "Tennis" OR "Track and Field" OR "Volleyball" OR "Walking" OR "Water Sports" OR "Weight Lifting" OR "Wrestling" OR "Youth Sports" OR "Qigong" OR "Dance Therapy" OR "Tai Ji" OR "Yoga" OR "Physiotherapy" OR "Physical Therapy" OR "physiotherapy" OR "physiotherapy*" OR "Dry Needling" OR "Electrotherapy" OR "Shock wave Therapy" OR "Balneotherapy" OR "Musculoskeletal Manipulation" OR "Dry Needling" OR "Electric Stimulation Therapy" OR "Electroacupuncture" OR "Pulsed Radiofrequency Treatment" OR "Spinal Cord Stimulation" OR "Transcutaneous Electric Nerve Stimulation" OR "Extracorporeal Shockwave Therapy" OR "Hydrotherapy" OR "Aquatic Therapy" OR "Therapeutic Irrigation" OR "Musculoskeletal Manipulations" OR "Applied Kinesiology" OR "Orthopedic Manipulation" OR "Orthopaedic Manipulation" OR "Osteopathic Manipulation" OR "Spinal Manipulation" OR "Continuous Passive Motion Therapy" OR "Soft Tissue Therapy" OR "Acupressure" OR "Massage" OR "Manual Lymphatic Drainage" OR "Myofascial Release Therapy" OR "mobilization" OR "mobilization" OR "mobilisation" OR "mobilizing" OR "mobilising" OR "mobilize" OR "mobilise" OR "mobilized" OR "mobilised" OR "mobility") AND TI**("elderly" OR "elder" OR "elders" OR "geriatr*" OR "Home for the Aged" OR "Elderly care" OR "Senior Center" OR "Nonagenarians" OR "Nonagenarian" OR "Octogenarians" OR "Octogenarian" OR "Centenarians" OR "Centenarian" OR "septuagenarian" OR "septuagenarians" OR "geront*" OR "older person" OR "old person" OR "older patient" OR "old patient" OR "older persons" OR "old persons" OR "older patients" OR "old patients" OR "older women" OR "old women" OR "older men" OR "old men" OR "old adult" OR "older adult" OR "old adults" OR "older adults" OR "Older individual" OR "Older individuals" OR "old people" OR "older people" OR "Oldest Old" OR "older population" OR "aging population" OR "aging population" OR "old aged" OR "old age" OR (("older" NEAR/3 "person") OR ("old" NEAR/3 "person") OR ("older" NEAR/3 "patient") OR ("old" NEAR/3 "patient") OR ("older" NEAR/3 "persons") OR ("old" NEAR/3 "persons") OR ("older" NEAR/3 "patients") OR ("old" NEAR/3 "patients") OR ("older" NEAR/3 "women") OR ("old" NEAR/3 "women") OR ("older" NEAR/3 "men") OR ("old" NEAR/3 "men") OR ("old" NEAR/3 "adult") OR ("older" NEAR/3 "adult") OR ("old" NEAR/3 "adults") OR ("older" NEAR/3 "adults") OR ("Older" NEAR/3 "individual") OR ("Older" NEAR/3 "individuals") OR ("old" NEAR/3 "people") OR ("older" NEAR/3 "people") OR ("Oldest" NEAR/3 "Old") OR ("older" NEAR/3 "population") OR ("aging" NEAR/3 "population") OR ("aging" NEAR/3 "population") OR ("old" NEAR/3 "aged") OR ("old" NEAR/3 "age") OR (**"60" NEAR/3 "year" NEAR/3 "old") OR ("61" NEAR/3 "year" NEAR/3 "old") OR ("62" NEAR/3 "year" NEAR/3 "old") OR ("63" NEAR/3 "year" NEAR/3 "old") OR ("64" NEAR/3 "year" NEAR/3 "old") OR ("65" NEAR/3 "year" NEAR/3 "old") OR ("66" NEAR/3 "year" NEAR/3 "old") OR ("67" NEAR/3 "year" NEAR/3 "old") OR ("68" NEAR/3 "year" NEAR/3 "old") OR ("69" NEAR/3 "year" NEAR/3 "old") OR ("60" NEAR/3 "years" NEAR/3 "old") OR ("61" NEAR/3 "years" NEAR/3 "old") OR ("62" NEAR/3 "years" NEAR/3 "old") OR ("63" NEAR/3 "years" NEAR/3 "old") OR ("64" NEAR/3 "years" NEAR/3 "old") OR ("65" NEAR/3 "years" NEAR/3 "old") OR ("66" NEAR/3 "years" NEAR/3 "old") OR ("67" NEAR/3 "years" NEAR/3 "old") OR ("68" NEAR/3 "years" NEAR/3 "old") OR ("69" NEAR/3 "years" NEAR/3 "old") OR ("70" NEAR/3 "year" NEAR/3 "old") OR ("71" NEAR/3 "year" NEAR/3 "old") OR ("72" NEAR/3 "year" NEAR/3 "old") OR ("73" NEAR/3 "year" NEAR/3 "old") OR ("74" NEAR/3 "year" NEAR/3 "old") OR ("75" NEAR/3 "year" NEAR/3 "old") OR ("76" NEAR/3 "year" NEAR/3 "old") OR ("77" NEAR/3 "year" NEAR/3 "old") OR ("78" NEAR/3 "year" NEAR/3 "old") OR ("79" NEAR/3 "year" NEAR/3 "old") OR ("70" NEAR/3 "years" NEAR/3 "old") OR ("71" NEAR/3 "years" NEAR/3 "old") OR ("72" NEAR/3 "years" NEAR/3 "old") OR ("73" NEAR/3 "years" NEAR/3 "old") OR ("74" NEAR/3 "years" NEAR/3 "old") OR ("75" NEAR/3 "years" NEAR/3 "old") OR ("76" NEAR/3 "years" NEAR/3 "old") OR ("77" NEAR/3 "years" NEAR/3 "old") OR ("78" NEAR/3 "years" NEAR/3 "old") OR ("79" NEAR/3 "years" NEAR/3 "old") OR ("80" NEAR/3 "year" NEAR/3 "old") OR ("81" NEAR/3 "year" NEAR/3 "old") OR ("82" NEAR/3 "year" NEAR/3 "old") OR ("83" NEAR/3 "year" NEAR/3 "old") OR ("84" NEAR/3 "year" NEAR/3 "old") OR ("85" NEAR/3 "year" NEAR/3 "old") OR ("86" NEAR/3 "year" NEAR/3 "old") OR ("87" NEAR/3 "year" NEAR/3 "old") OR ("88" NEAR/3 "year" NEAR/3 "old") OR ("89" NEAR/3 "year" NEAR/3 "old") OR ("80" NEAR/3 "years" NEAR/3 "old") OR ("81" NEAR/3 "years" NEAR/3 "old") OR ("82" NEAR/3 "years" NEAR/3 "old") OR ("83" NEAR/3 "years" NEAR/3 "old") OR ("84" NEAR/3 "years" NEAR/3 "old") OR ("85" NEAR/3 "years" NEAR/3 "old") OR ("86" NEAR/3 "years" NEAR/3 "old") OR ("87" NEAR/3 "years" NEAR/3 "old") OR ("88" NEAR/3 "years" NEAR/3 "old") OR ("89" NEAR/3 "years" NEAR/3 "old") OR ("90" NEAR/3 "year" NEAR/3 "old") OR ("91" NEAR/3 "year" NEAR/3 "old") OR ("92" NEAR/3 "year" NEAR/3 "old") OR ("93" NEAR/3 "year" NEAR/3 "old") OR ("94" NEAR/3 "year" NEAR/3 "old") OR ("95" NEAR/3 "year" NEAR/3 "old") OR ("96" NEAR/3 "year" NEAR/3 "old") OR ("97" NEAR/3 "year" NEAR/3 "old") OR ("98" NEAR/3 "year" NEAR/3 "old") OR ("99" NEAR/3 "year" NEAR/3 "old") OR ("90" NEAR/3 "years" NEAR/3 "old") OR ("91" NEAR/3 "years" NEAR/3 "old") OR ("92" NEAR/3 "years" NEAR/3 "old") OR ("93" NEAR/3 "years" NEAR/3 "old") OR ("94" NEAR/3 "years" NEAR/3 "old") OR ("95" NEAR/3 "years" NEAR/3 "old") OR ("96" NEAR/3 "years" NEAR/3 "old") OR ("97" NEAR/3 "years" NEAR/3 "old") OR ("98" NEAR/3 "years" NEAR/3 "old") OR ("99" NEAR/3 "years" NEAR/3 "old") OR ("100" NEAR/3 "year" NEAR/3 "old") OR ("101" NEAR/3 "year" NEAR/3 "old") OR ("102" NEAR/3 "year" NEAR/3 "old") OR ("103" NEAR/3 "year" NEAR/3 "old") OR ("104" NEAR/3 "year" NEAR/3 "old") OR ("105" NEAR/3 "year" NEAR/3 "old") OR ("106" NEAR/3 "year" NEAR/3 "old") OR ("107" NEAR/3 "year" NEAR/3 "old") OR ("108" NEAR/3 "year" NEAR/3 "old") OR ("109" NEAR/3 "year" NEAR/3 "old") OR ("100" NEAR/3 "years" NEAR/3 "old") OR ("101" NEAR/3 "years" NEAR/3 "old") OR ("102" NEAR/3 "years" NEAR/3 "old") OR ("103" NEAR/3 "years" NEAR/3 "old") OR ("104" NEAR/3 "years" NEAR/3 "old") OR ("105" NEAR/3 "years" NEAR/3 "old") OR ("106" NEAR/3 "years" NEAR/3 "old") OR ("107" NEAR/3 "years" NEAR/3 "old") OR ("108" NEAR/3 "years" NEAR/3 "old") OR ("109" NEAR/3 "years" NEAR/3 "old"**)))) OR** ((TI("Physical Activity" OR "Exercise" OR "Exercise" OR "Exercises" OR "Exercising" OR "Exercis*" OR "physical activity" OR "physical activities" OR "Endurance Training" OR "Exergaming" OR "Gymnastics" OR "Gymnastic" OR "Interval Training" OR "Jogging" OR "Motor Activity" OR "Movement" OR "Muscle Stretching"OR "Physical Conditioning" OR "Resistance Training" OR "Running" OR "Stair Climbing" OR "Swimming" OR "Training" OR "Walking" OR "Kinesiotherapy" OR "Physical Exertion" OR "Physical Exertions" OR "Physical Effort" OR "Physical Efforts" OR "Sport" OR "Sports" OR "Sport" OR "Athletic Performance" OR "Baseball" OR "Basketball" OR "Bicycling" OR "Boxing" OR "Cardiorespiratory Fitness" OR "Cardiorespiratory Fitness" OR "Cricket Sport" OR "Diving" OR "Football" OR "Golf" OR "Gymnastics" OR "Hockey" OR "Jogging" OR "Marathon Running" OR "Martial Arts" OR "Mountaineering" OR "Nordic Walking" OR "Physical Endurance" OR "Physical Fitness" OR "Racquet Sports" OR "Return to Sport" OR "Rugby" OR "Running" OR "Skating" OR "Skiing" OR "Snow Sports" OR "Soccer" OR "Swimming" OR "Tai Ji" OR "Team Sports" OR "Tennis" OR "Track and Field" OR "Volleyball" OR "Walking" OR "Water Sports" OR "Weight Lifting" OR "Wrestling" OR "Youth Sports" OR "Qigong" OR "Dance Therapy" OR "Tai Ji" OR "Yoga" OR "Physiotherapy" OR "Physical Therapy" OR "physiotherapy" OR "physiotherapy*" OR "Dry Needling" OR "Electrotherapy" OR "Shock wave Therapy" OR "Balneotherapy" OR "Musculoskeletal Manipulation" OR "Dry Needling" OR "Electric Stimulation Therapy" OR "Electroacupuncture" OR "Pulsed Radiofrequency Treatment" OR "Spinal Cord Stimulation" OR "Transcutaneous Electric Nerve Stimulation" OR "Extracorporeal Shockwave Therapy" OR "Hydrotherapy" OR "Aquatic Therapy" OR "Therapeutic Irrigation" OR "Musculoskeletal Manipulations" OR "Applied Kinesiology" OR "Orthopedic Manipulation" OR "Orthopaedic Manipulation" OR "Osteopathic Manipulation" OR "Spinal Manipulation" OR "Continuous Passive Motion Therapy" OR "Soft Tissue Therapy" OR "Acupressure" OR "Massage" OR "Manual Lymphatic Drainage" OR "Myofascial Release Therapy" OR "mobilization" OR "mobilization" OR "mobilisation" OR "mobilizing" OR "mobilising" OR "mobilize" OR "mobilise" OR "mobilized" OR "mobilised" OR "mobility") OR SU("Physical Activity" OR "Exercise" OR "Exercise" OR "Exercises" OR "Exercising" OR "Exercis*" OR "physical activity" OR "physical activities" OR "Endurance Training" OR "Exergaming" OR "Gymnastics" OR "Gymnastic" OR "Interval Training" OR "Jogging" OR "Motor Activity" OR "Movement" OR "Muscle Stretching"OR "Physical Conditioning" OR "Resistance Training" OR "Running" OR "Stair Climbing" OR "Swimming" OR "Training" OR "Walking" OR "Kinesiotherapy" OR "Physical Exertion" OR "Physical Exertions" OR "Physical Effort" OR "Physical Efforts" OR "Sport" OR "Sports" OR "Sport" OR "Athletic Performance" OR "Baseball" OR "Basketball" OR "Bicycling" OR "Boxing" OR "Cardiorespiratory Fitness" OR "Cardiorespiratory Fitness" OR "Cricket Sport" OR "Diving" OR "Football" OR "Golf" OR "Gymnastics" OR "Hockey" OR "Jogging" OR "Marathon Running" OR "Martial Arts" OR "Mountaineering" OR "Nordic Walking" OR "Physical Endurance" OR "Physical Fitness" OR "Racquet Sports" OR "Return to Sport" OR "Rugby" OR "Running" OR "Skating" OR "Skiing" OR "Snow Sports" OR "Soccer" OR "Swimming" OR "Tai Ji" OR "Team Sports" OR "Tennis" OR "Track and Field" OR "Volleyball" OR "Walking" OR "Water Sports" OR "Weight Lifting" OR "Wrestling" OR "Youth Sports" OR "Qigong" OR "Dance Therapy" OR "Tai Ji" OR "Yoga" OR "Physiotherapy" OR "Physical Therapy" OR "physiotherapy" OR "physiotherapy*" OR "Dry Needling" OR "Electrotherapy" OR "Shock wave Therapy" OR "Balneotherapy" OR "Musculoskeletal Manipulation" OR "Dry Needling" OR "Electric Stimulation Therapy" OR "Electroacupuncture" OR "Pulsed Radiofrequency Treatment" OR "Spinal Cord Stimulation" OR "Transcutaneous Electric Nerve Stimulation" OR "Extracorporeal Shockwave Therapy" OR "Hydrotherapy" OR "Aquatic Therapy" OR "Therapeutic Irrigation" OR "Musculoskeletal Manipulations" OR "Applied Kinesiology" OR "Orthopedic Manipulation" OR "Orthopaedic Manipulation" OR "Osteopathic Manipulation" OR "Spinal Manipulation" OR "Continuous Passive Motion Therapy" OR "Soft Tissue Therapy" OR "Acupressure" OR "Massage" OR "Manual Lymphatic Drainage" OR "Myofascial Release Therapy" OR "mobilization" OR "mobilization" OR "mobilisation" OR "mobilizing" OR "mobilising" OR "mobilize" OR "mobilise" OR "mobilized" OR "mobilised" OR "mobility") OR KW("Physical Activity" OR "Exercise" OR "Exercise" OR "Exercises" OR "Exercising" OR "Exercis*" OR "physical activity" OR "physical activities" OR "Endurance Training" OR "Exergaming" OR "Gymnastics" OR "Gymnastic" OR "Interval Training" OR "Jogging" OR "Motor Activity" OR "Movement" OR "Muscle Stretching"OR "Physical Conditioning" OR "Resistance Training" OR "Running" OR "Stair Climbing" OR "Swimming" OR "Training" OR "Walking" OR "Kinesiotherapy" OR "Physical Exertion" OR "Physical Exertions" OR "Physical Effort" OR "Physical Efforts" OR "Sport" OR "Sports" OR "Sport" OR "Athletic Performance" OR "Baseball" OR "Basketball" OR "Bicycling" OR "Boxing" OR "Cardiorespiratory Fitness" OR "Cardiorespiratory Fitness" OR "Cricket Sport" OR "Diving" OR "Football" OR "Golf" OR "Gymnastics" OR "Hockey" OR "Jogging" OR "Marathon Running" OR "Martial Arts" OR "Mountaineering" OR "Nordic Walking" OR "Physical Endurance" OR "Physical Fitness" OR "Racquet Sports" OR "Return to Sport" OR "Rugby" OR "Running" OR "Skating" OR "Skiing" OR "Snow Sports" OR "Soccer" OR "Swimming" OR "Tai Ji" OR "Team Sports" OR "Tennis" OR "Track and Field" OR "Volleyball" OR "Walking" OR "Water Sports" OR "Weight Lifting" OR "Wrestling" OR "Youth Sports" OR "Qigong" OR "Dance Therapy" OR "Tai Ji" OR "Yoga" OR "Physiotherapy" OR "Physical Therapy" OR "physiotherapy" OR "physiotherapy*" OR "Dry Needling" OR "Electrotherapy" OR "Shock wave Therapy" OR "Balneotherapy" OR "Musculoskeletal Manipulation" OR "Dry Needling" OR "Electric Stimulation Therapy" OR "Electroacupuncture" OR "Pulsed Radiofrequency Treatment" OR "Spinal Cord Stimulation" OR "Transcutaneous Electric Nerve Stimulation" OR "Extracorporeal Shockwave Therapy" OR "Hydrotherapy" OR "Aquatic Therapy" OR "Therapeutic Irrigation" OR "Musculoskeletal Manipulations" OR "Applied Kinesiology" OR "Orthopedic Manipulation" OR "Orthopaedic Manipulation" OR "Osteopathic Manipulation" OR "Spinal Manipulation" OR "Continuous Passive Motion Therapy" OR "Soft Tissue Therapy" OR "Acupressure" OR "Massage" OR "Manual Lymphatic Drainage" OR "Myofascial Release Therapy" OR "mobilization" OR "mobilization" OR "mobilisation" OR "mobilizing" OR "mobilising" OR "mobilize" OR "mobilise" OR "mobilized" OR "mobilised" OR "mobility") OR AB("Physical Activity" OR "Exercise" OR "Exercise" OR "Exercises" OR "Exercising" OR "Exercis*" OR "physical activity" OR "physical activities" OR "Endurance Training" OR "Exergaming" OR "Gymnastics" OR "Gymnastic" OR "Interval Training" OR "Jogging" OR "Motor Activity" OR "Movement" OR "Muscle Stretching"OR "Physical Conditioning" OR "Resistance Training" OR "Running" OR "Stair Climbing" OR "Swimming" OR "Training" OR "Walking" OR "Kinesiotherapy" OR "Physical Exertion" OR "Physical Exertions" OR "Physical Effort" OR "Physical Efforts" OR "Sport" OR "Sports" OR "Sport" OR "Athletic Performance" OR "Baseball" OR "Basketball" OR "Bicycling" OR "Boxing" OR "Cardiorespiratory Fitness" OR "Cardiorespiratory Fitness" OR "Cricket Sport" OR "Diving" OR "Football" OR "Golf" OR "Gymnastics" OR "Hockey" OR "Jogging" OR "Marathon Running" OR "Martial Arts" OR "Mountaineering" OR "Nordic Walking" OR "Physical Endurance" OR "Physical Fitness" OR "Racquet Sports" OR "Return to Sport" OR "Rugby" OR "Running" OR "Skating" OR "Skiing" OR "Snow Sports" OR "Soccer" OR "Swimming" OR "Tai Ji" OR "Team Sports" OR "Tennis" OR "Track and Field" OR "Volleyball" OR "Walking" OR "Water Sports" OR "Weight Lifting" OR "Wrestling" OR "Youth Sports" OR "Qigong" OR "Dance Therapy" OR "Tai Ji" OR "Yoga" OR "Physiotherapy" OR "Physical Therapy" OR "physiotherapy" OR "physiotherapy*" OR "Dry Needling" OR "Electrotherapy" OR "Shock wave Therapy" OR "Balneotherapy" OR "Musculoskeletal Manipulation" OR "Dry Needling" OR "Electric Stimulation Therapy" OR "Electroacupuncture" OR "Pulsed Radiofrequency Treatment" OR "Spinal Cord Stimulation" OR "Transcutaneous Electric Nerve Stimulation" OR "Extracorporeal Shockwave Therapy" OR "Hydrotherapy" OR "Aquatic Therapy" OR "Therapeutic Irrigation" OR "Musculoskeletal Manipulations" OR "Applied Kinesiology" OR "Orthopedic Manipulation" OR "Orthopaedic Manipulation" OR "Osteopathic Manipulation" OR "Spinal Manipulation" OR "Continuous Passive Motion Therapy" OR "Soft Tissue Therapy" OR "Acupressure" OR "Massage" OR "Manual Lymphatic Drainage" OR "Myofascial Release Therapy" OR "mobilization" OR "mobilization" OR "mobilisation" OR "mobilizing" OR "mobilising" OR "mobilize" OR "mobilise" OR "mobilized" OR "mobilised" OR "mobility")) AND TI("Ankle Fracture" OR "Femur Fracture" OR "Fibula Fracture" OR "Knee Fracture" OR "Tibia Fracture" OR "Ankle Fractures" OR "Femoral Fractures" OR "Hip Fractures" OR "Femoral Neck Fractures" OR "Proximal Femoral Fractures" OR "Hoffa Fracture" OR "Proximal Femoral Fractures" OR "Fibula Fractures" OR "Knee Fractures" OR "Hoffa Fracture" OR "Patella Fracture" OR "Tibial Plateau Fractures" OR "Tibial Fractures" OR "Tibial Plateau Fractures" OR "Ankle Fracture" OR "Femoral Fracture" OR "Hip Fracture" OR "Femoral Neck Fracture" OR "Proximal Femoral Fracture" OR "Hoffa Fractures" OR "Proximal Femoral Fracture" OR "Fibula Fracture" OR "Knee Fracture" OR "Hoffa Fractures" OR "Patella Fractures" OR "Tibial Plateau Fracture" OR "Tibial Fracture" OR "Tibial Plateau Fracture" OR (("Lower Limb" OR "lower limb" OR "lower limbs" OR "lower extremity" OR "lower extremities" OR "Ankle" OR "Foot" OR "Heel" OR "Hip" OR "Knee" OR "Leg" OR "Ankles" OR "Feet" OR "Heels" OR "Hips" OR "Knees" OR "Legs") AND ("Fracture" OR "fracture" OR "fractures" OR "fractur*")) OR **"acutely ill"**) AND (TI("immobility" OR "immobility" OR "immobile" OR "immobil*" OR "temporary" OR "non weight bearing" OR "non weight bearing" OR "nonweight bearing" OR "non weight bear*" OR "nonweight bear*" OR "physically dependent" OR "physically disabled" OR **"physically dependent" OR "physically disabled" OR "disuse" OR "bed rest" OR "bed rest" OR "bedrest" OR "leg immobilization" OR "leg immobilisation" OR "Weightlessness" OR "deconditioning" OR "deconditioning" OR "permissive weight-bearing"**) OR SU("immobility" OR "immobility" OR "immobile" OR "immobil*" OR "temporary" OR "non weight bearing" OR "non weight bearing" OR "nonweight bearing" OR "non weight bear*" OR "nonweight bear*" OR "physically dependent" OR "physically disabled" OR **"physically dependent" OR "physically disabled" OR "disuse" OR "bed rest" OR "bed rest" OR "bedrest" OR "leg immobilization" OR "leg immobilisation" OR "Weightlessness" OR "deconditioning" OR "deconditioning" OR "permissive weight-bearing"**) OR KW("immobility" OR "immobility" OR "immobile" OR "immobil*" OR "temporary" OR "non weight bearing" OR "non weight bearing" OR "nonweight bearing" OR "non weight bear*" OR "nonweight bear*" OR "physically dependent" OR "physically disabled" OR **"physically dependent" OR "physically disabled" OR "disuse" OR "bed rest" OR "bed rest" OR "bedrest" OR "leg immobilization" OR "leg immobilisation" OR "Weightlessness" OR "deconditioning" OR "deconditioning" OR "permissive weight-bearing"**) OR AB("immobility" OR "immobility" OR "immobile" OR "immobil*" OR "temporary" OR "non weight bearing" OR "non weight bearing" OR "nonweight bearing" OR "non weight bear*" OR "nonweight bear*" OR "physically dependent" OR "physically disabled" OR **"physically dependent" OR "physically disabled" OR "disuse" OR "bed rest" OR "bed rest" OR "bedrest" OR "leg immobilization" OR "leg immobilisation" OR "Weightlessness" OR "deconditioning" OR "deconditioning" OR "permissive weight-bearing"**))**) OR** (TI("Physical Activity" OR "Exercise" OR "Exercise" OR "Exercises" OR "Exercising" OR "Exercis*" OR "physical activity" OR "physical activities" OR "Endurance Training" OR "Exergaming" OR "Gymnastics" OR "Gymnastic" OR "Interval Training" OR "Jogging" OR "Motor Activity" OR "Movement" OR "Muscle Stretching"OR "Physical Conditioning" OR "Resistance Training" OR "Running" OR "Stair Climbing" OR "Swimming" OR "Training" OR "Walking" OR "Kinesiotherapy" OR "Physical Exertion" OR "Physical Exertions" OR "Physical Effort" OR "Physical Efforts" OR "Sport" OR "Sports" OR "Sport" OR "Athletic Performance" OR "Baseball" OR "Basketball" OR "Bicycling" OR "Boxing" OR "Cardiorespiratory Fitness" OR "Cardiorespiratory Fitness" OR "Cricket Sport" OR "Diving" OR "Football" OR "Golf" OR "Gymnastics" OR "Hockey" OR "Jogging" OR "Marathon Running" OR "Martial Arts" OR "Mountaineering" OR "Nordic Walking" OR "Physical Endurance" OR "Physical Fitness" OR "Racquet Sports" OR "Return to Sport" OR "Rugby" OR "Running" OR "Skating" OR "Skiing" OR "Snow Sports" OR "Soccer" OR "Swimming" OR "Tai Ji" OR "Team Sports" OR "Tennis" OR "Track and Field" OR "Volleyball" OR "Walking" OR "Water Sports" OR "Weight Lifting" OR "Wrestling" OR "Youth Sports" OR "Qigong" OR "Dance Therapy" OR "Tai Ji" OR "Yoga" OR "Physiotherapy" OR "Physical Therapy" OR "physiotherapy" OR "physiotherapy*" OR "Dry Needling" OR "Electrotherapy" OR "Shock wave Therapy" OR "Balneotherapy" OR "Musculoskeletal Manipulation" OR "Dry Needling" OR "Electric Stimulation Therapy" OR "Electroacupuncture" OR "Pulsed Radiofrequency Treatment" OR "Spinal Cord Stimulation" OR "Transcutaneous Electric Nerve Stimulation" OR "Extracorporeal Shockwave Therapy" OR "Hydrotherapy" OR "Aquatic Therapy" OR "Therapeutic Irrigation" OR "Musculoskeletal Manipulations" OR "Applied Kinesiology" OR "Orthopedic Manipulation" OR "Orthopaedic Manipulation" OR "Osteopathic Manipulation" OR "Spinal Manipulation" OR "Continuous Passive Motion Therapy" OR "Soft Tissue Therapy" OR "Acupressure" OR "Massage" OR "Manual Lymphatic Drainage" OR "Myofascial Release Therapy" OR "mobilization" OR "mobilization" OR "mobilisation" OR "mobilizing" OR "mobilising" OR "mobilize" OR "mobilise" OR "mobilized" OR "mobilised" OR "mobility") AND (TI(**"disuse"**) OR SU(**"disuse"**) OR KW(**"disuse"**) OR AB(**"disuse"**)) AND TI**("elderly" OR "elder" OR "elders" OR "geriatr*" OR "Home for the Aged" OR "Elderly care" OR "Senior Center" OR "Nonagenarians" OR "Nonagenarian" OR "Octogenarians" OR "Octogenarian" OR "Centenarians" OR "Centenarian" OR "septuagenarian" OR "septuagenarians" OR "geront*" OR "older person" OR "old person" OR "older patient" OR "old patient" OR "older persons" OR "old persons" OR "older patients" OR "old patients" OR "older women" OR "old women" OR "older men" OR "old men" OR "old adult" OR "older adult" OR "old adults" OR "older adults" OR "Older individual" OR "Older individuals" OR "old people" OR "older people" OR "Oldest Old" OR "older population" OR "aging population" OR "aging population" OR "old aged" OR "old age" OR (("older" NEAR/3 "person") OR ("old" NEAR/3 "person") OR ("older" NEAR/3 "patient") OR ("old" NEAR/3 "patient") OR ("older" NEAR/3 "persons") OR ("old" NEAR/3 "persons") OR ("older" NEAR/3 "patients") OR ("old" NEAR/3 "patients") OR ("older" NEAR/3 "women") OR ("old" NEAR/3 "women") OR ("older" NEAR/3 "men") OR ("old" NEAR/3 "men") OR ("old" NEAR/3 "adult") OR ("older" NEAR/3 "adult") OR ("old" NEAR/3 "adults") OR ("older" NEAR/3 "adults") OR ("Older" NEAR/3 "individual") OR ("Older" NEAR/3 "individuals") OR ("old" NEAR/3 "people") OR ("older" NEAR/3 "people") OR ("Oldest" NEAR/3 "Old") OR ("older" NEAR/3 "population") OR ("aging" NEAR/3 "population") OR ("aging" NEAR/3 "population") OR ("old" NEAR/3 "aged") OR ("old" NEAR/3 "age") OR (**"60" NEAR/3 "year" NEAR/3 "old") OR ("61" NEAR/3 "year" NEAR/3 "old") OR ("62" NEAR/3 "year" NEAR/3 "old") OR ("63" NEAR/3 "year" NEAR/3 "old") OR ("64" NEAR/3 "year" NEAR/3 "old") OR ("65" NEAR/3 "year" NEAR/3 "old") OR ("66" NEAR/3 "year" NEAR/3 "old") OR ("67" NEAR/3 "year" NEAR/3 "old") OR ("68" NEAR/3 "year" NEAR/3 "old") OR ("69" NEAR/3 "year" NEAR/3 "old") OR ("60" NEAR/3 "years" NEAR/3 "old") OR ("61" NEAR/3 "years" NEAR/3 "old") OR ("62" NEAR/3 "years" NEAR/3 "old") OR ("63" NEAR/3 "years" NEAR/3 "old") OR ("64" NEAR/3 "years" NEAR/3 "old") OR ("65" NEAR/3 "years" NEAR/3 "old") OR ("66" NEAR/3 "years" NEAR/3 "old") OR ("67" NEAR/3 "years" NEAR/3 "old") OR ("68" NEAR/3 "years" NEAR/3 "old") OR ("69" NEAR/3 "years" NEAR/3 "old") OR ("70" NEAR/3 "year" NEAR/3 "old") OR ("71" NEAR/3 "year" NEAR/3 "old") OR ("72" NEAR/3 "year" NEAR/3 "old") OR ("73" NEAR/3 "year" NEAR/3 "old") OR ("74" NEAR/3 "year" NEAR/3 "old") OR ("75" NEAR/3 "year" NEAR/3 "old") OR ("76" NEAR/3 "year" NEAR/3 "old") OR ("77" NEAR/3 "year" NEAR/3 "old") OR ("78" NEAR/3 "year" NEAR/3 "old") OR ("79" NEAR/3 "year" NEAR/3 "old") OR ("70" NEAR/3 "years" NEAR/3 "old") OR ("71" NEAR/3 "years" NEAR/3 "old") OR ("72" NEAR/3 "years" NEAR/3 "old") OR ("73" NEAR/3 "years" NEAR/3 "old") OR ("74" NEAR/3 "years" NEAR/3 "old") OR ("75" NEAR/3 "years" NEAR/3 "old") OR ("76" NEAR/3 "years" NEAR/3 "old") OR ("77" NEAR/3 "years" NEAR/3 "old") OR ("78" NEAR/3 "years" NEAR/3 "old") OR ("79" NEAR/3 "years" NEAR/3 "old") OR ("80" NEAR/3 "year" NEAR/3 "old") OR ("81" NEAR/3 "year" NEAR/3 "old") OR ("82" NEAR/3 "year" NEAR/3 "old") OR ("83" NEAR/3 "year" NEAR/3 "old") OR ("84" NEAR/3 "year" NEAR/3 "old") OR ("85" NEAR/3 "year" NEAR/3 "old") OR ("86" NEAR/3 "year" NEAR/3 "old") OR ("87" NEAR/3 "year" NEAR/3 "old") OR ("88" NEAR/3 "year" NEAR/3 "old") OR ("89" NEAR/3 "year" NEAR/3 "old") OR ("80" NEAR/3 "years" NEAR/3 "old") OR ("81" NEAR/3 "years" NEAR/3 "old") OR ("82" NEAR/3 "years" NEAR/3 "old") OR ("83" NEAR/3 "years" NEAR/3 "old") OR ("84" NEAR/3 "years" NEAR/3 "old") OR ("85" NEAR/3 "years" NEAR/3 "old") OR ("86" NEAR/3 "years" NEAR/3 "old") OR ("87" NEAR/3 "years" NEAR/3 "old") OR ("88" NEAR/3 "years" NEAR/3 "old") OR ("89" NEAR/3 "years" NEAR/3 "old") OR ("90" NEAR/3 "year" NEAR/3 "old") OR ("91" NEAR/3 "year" NEAR/3 "old") OR ("92" NEAR/3 "year" NEAR/3 "old") OR ("93" NEAR/3 "year" NEAR/3 "old") OR ("94" NEAR/3 "year" NEAR/3 "old") OR ("95" NEAR/3 "year" NEAR/3 "old") OR ("96" NEAR/3 "year" NEAR/3 "old") OR ("97" NEAR/3 "year" NEAR/3 "old") OR ("98" NEAR/3 "year" NEAR/3 "old") OR ("99" NEAR/3 "year" NEAR/3 "old") OR ("90" NEAR/3 "years" NEAR/3 "old") OR ("91" NEAR/3 "years" NEAR/3 "old") OR ("92" NEAR/3 "years" NEAR/3 "old") OR ("93" NEAR/3 "years" NEAR/3 "old") OR ("94" NEAR/3 "years" NEAR/3 "old") OR ("95" NEAR/3 "years" NEAR/3 "old") OR ("96" NEAR/3 "years" NEAR/3 "old") OR ("97" NEAR/3 "years" NEAR/3 "old") OR ("98" NEAR/3 "years" NEAR/3 "old") OR ("99" NEAR/3 "years" NEAR/3 "old") OR ("100" NEAR/3 "year" NEAR/3 "old") OR ("101" NEAR/3 "year" NEAR/3 "old") OR ("102" NEAR/3 "year" NEAR/3 "old") OR ("103" NEAR/3 "year" NEAR/3 "old") OR ("104" NEAR/3 "year" NEAR/3 "old") OR ("105" NEAR/3 "year" NEAR/3 "old") OR ("106" NEAR/3 "year" NEAR/3 "old") OR ("107" NEAR/3 "year" NEAR/3 "old") OR ("108" NEAR/3 "year" NEAR/3 "old") OR ("109" NEAR/3 "year" NEAR/3 "old") OR ("100" NEAR/3 "years" NEAR/3 "old") OR ("101" NEAR/3 "years" NEAR/3 "old") OR ("102" NEAR/3 "years" NEAR/3 "old") OR ("103" NEAR/3 "years" NEAR/3 "old") OR ("104" NEAR/3 "years" NEAR/3 "old") OR ("105" NEAR/3 "years" NEAR/3 "old") OR ("106" NEAR/3 "years" NEAR/3 "old") OR ("107" NEAR/3 "years" NEAR/3 "old") OR ("108" NEAR/3 "years" NEAR/3 "old") OR ("109" NEAR/3 "years" NEAR/3 "old"**))))) NOT TI("veterinary" OR "rabbit" OR "rabbits" OR "animal" OR "animals" OR "mouse" OR "mice" OR "rodent" OR "rodents" OR "rat" OR "rats" OR "pig" OR "pigs" OR "porcine" OR "horse" OR "horses" OR "equine" OR "cow" OR "cows" OR "bovine" OR "goat" OR "goats" OR "sheep" OR "ovine" OR "canine" OR "dog" OR "dogs" OR "feline" OR "cat" OR "cats")** NOT TI(("Case Report" OR "review") NOT ("Clinical Trial" OR "trial" OR "RCT" OR "systematic"))

**PEDro**

Eight queries

**Body part: lower leg or knee**

**Abstract & Title: wheelchair**

**Body part: lower leg or knee**

**Abstract & Title: wheelchairs**

**Body part: foot or ankle**

**Abstract & Title: wheelchair**

**Body part: foot or ankle**

**Abstract & Title: wheelchairs**

**Body part: lower leg or knee**

**Abstract & Title: immobility**

**Body part: foot or ankle**

**Abstract & Title: immobility**

**Body part: lower leg or knee**

**Abstract & Title: disuse**

**Body part: foot or ankle**

**Abstract & Title: disuse**

**Epistemonikos database**

Five queries

Query 1

**Title/abstract**

("Wheelchair" OR "Wheelchairs" OR "Wheelchair" OR "Wheel chairs" OR "Wheel chair" OR "Mobility Scooter" OR "Mobility Scooters")

AND

**Title/abstract**

("Physical Activity" OR "Exercise" OR "mobilization" OR "mobilization" OR "mobilisation" OR "mobilizing" OR "mobilising" OR "mobilize" OR "mobilise" OR "mobilized" OR "mobilised" OR "mobility")

AND

**Title/abstract**

(("Lower Limb" OR "lower limb" OR "lower limbs" OR "lower extremity" OR "lower extremities" OR "Ankle" OR "Foot" OR "Heel" OR "Hip" OR "Knee" OR "Leg" OR "Ankles" OR "Feet" OR "Heels" OR "Hips" OR "Knees" OR "Legs") AND ("Fracture" OR "fracture" OR "fractures"))

Query 2

**Title/abstract**

("Wheelchair" OR "Wheelchairs" OR "Wheelchair" OR "Wheel chairs" OR "Wheel chair" OR "Mobility Scooter" OR "Mobility Scooters")

AND

**Title/abstract**

("Physical Activity" OR "Exercise" OR "mobilization" OR "mobilization" OR "mobilisation" OR "mobilizing" OR "mobilising" OR "mobilize" OR "mobilise" OR "mobilized" OR "mobilised" OR "mobility")

AND

**Title/abstract**

("immobility" OR "immobile" OR "temporary" OR "non weight bearing" OR "non weight bearing" OR "nonweight bearing")

**Query 3**

**Title/abstract**

("Wheelchair" OR "Wheelchairs" OR "Wheelchair" OR "Wheel chairs" OR "Wheel chair" OR "Mobility Scooter" OR "Mobility Scooters")

AND

**Title/abstract**

("Physical Activity" OR "Exercise" OR "mobilization" OR "mobilization" OR "mobilisation" OR "mobilizing" OR "mobilising" OR "mobilize" OR "mobilise" OR "mobilized" OR "mobilised" OR "mobility")

Title/abstract

**("elderly" OR "elder" OR "elders" OR "geriatrics" OR "geriatric")**

**Query 4**

**Title/abstract**

("Physical Activity" OR "Exercise" OR "mobilization" OR "mobilization" OR "mobilisation" OR "mobilizing" OR "mobilising" OR "mobilize" OR "mobilise" OR "mobilized" OR "mobilised" OR "mobility")

**Title/abstract**

(("Lower Limb" OR "lower limb" OR "lower limbs" OR "lower extremity" OR "lower extremities" OR "Ankle" OR "Foot" OR "Heel" OR "Hip" OR "Knee" OR "Leg" OR "Ankles" OR "Feet" OR "Heels" OR "Hips" OR "Knees" OR "Legs") AND ("Fracture" OR "fracture" OR "fractures"))

**Title/abstract**

("immobility" OR "immobile" OR OR "temporary" OR "non weight bearing" OR "nonweight bearing" OR "physically dependent" OR "physically disabled" OR **"disuse" OR "bed rest" OR "bedrest" OR "leg immobilization" OR "leg immobilisation" OR "Weightlessness" OR "deconditioning" OR "permissive weight-bearing"**)

Query 5

**Title**

("Physical Activity" OR "Exercise" OR "mobilization" OR "mobilization" OR "mobilisation" OR "mobilizing" OR "mobilising" OR "mobilize" OR "mobilise" OR "mobilized" OR "mobilised" OR "mobility")

Title/abstract

**"disuse"**

Title/abstract

**("elderly" OR "elder" OR "elders" OR "geriatrics" OR "geriatric")**

**CINAHL**

<https://catalogue.leidenuniv.nl/view/action/uresolver.do?operation=resolveService&package_service_id=33442523690002711&institutionId=2711&customerId=2710&VE=true>

(replacing Emcare)

Limit to Academic Journals

Limit to English

**(**((TI("Wheelchair" OR "Wheelchairs" OR "Wheelchair" OR "Wheelchair*" OR "Wheel chairs" OR "Wheel chair" OR "Wheel chair*" OR "Mobility Scooter" OR "Mobility Scooters") OR SU("Wheelchair" OR "Wheelchairs" OR "Wheelchair" OR "Wheelchair*" OR "Wheel chairs" OR "Wheel chair" OR "Wheel chair*" OR "Mobility Scooter" OR "Mobility Scooters") OR MW("Wheelchair" OR "Wheelchairs" OR "Wheelchair" OR "Wheelchair*" OR "Wheel chairs" OR "Wheel chair" OR "Wheel chair*" OR "Mobility Scooter" OR "Mobility Scooters") OR AB("Wheelchair" OR "Wheelchairs" OR "Wheelchair" OR "Wheelchair*" OR "Wheel chairs" OR "Wheel chair" OR "Wheel chair*" OR "Mobility Scooter" OR "Mobility Scooters")) AND (TI("Physical Activity" OR "Exercise" OR "Exercise" OR "Exercises" OR "Exercising" OR "Exercis*" OR "physical activity" OR "physical activities" OR "Endurance Training" OR "Exergaming" OR "Gymnastics" OR "Gymnastic" OR "Interval Training" OR "Jogging" OR "Motor Activity" OR "Movement" OR "Muscle Stretching" OR "Physical Conditioning" OR "Resistance Training" OR "Running" OR "Stair Climbing" OR "Swimming" OR "Training" OR "Walking" OR "Kinesiotherapy" OR "Physical Exertion" OR "Physical Exertions" OR "Physical Effort" OR "Physical Efforts" OR "Sport" OR "Sports" OR "Sport" OR "Athletic Performance" OR "Baseball" OR "Basketball" OR "Bicycling" OR "Boxing" OR "Cardiorespiratory Fitness" OR "Cardiorespiratory Fitness" OR "Cricket Sport" OR "Diving" OR "Football" OR "Golf" OR "Gymnastics" OR "Hockey" OR "Jogging" OR "Marathon Running" OR "Martial Arts" OR "Mountaineering" OR "Nordic Walking" OR "Physical Endurance" OR "Physical Fitness" OR "Racquet Sports" OR "Return to Sport" OR "Rugby" OR "Running" OR "Skating" OR "Skiing" OR "Snow Sports" OR "Soccer" OR "Swimming" OR "Tai Ji" OR "Team Sports" OR "Tennis" OR "Track and Field" OR "Volleyball" OR "Walking" OR "Water Sports" OR "Weight Lifting" OR "Wrestling" OR "Youth Sports" OR "Qigong" OR "Dance Therapy" OR "Tai Ji" OR "Yoga" OR "Physiotherapy" OR "Physical Therapy" OR "physiotherapy" OR "physiotherapy*" OR "Dry Needling" OR "Electrotherapy" OR "Shock wave Therapy" OR "Balneotherapy" OR "Musculoskeletal Manipulation" OR "Dry Needling" OR "Electric Stimulation Therapy" OR "Electroacupuncture" OR "Pulsed Radiofrequency Treatment" OR "Spinal Cord Stimulation" OR "Transcutaneous Electric Nerve Stimulation" OR "Extracorporeal Shockwave Therapy" OR "Hydrotherapy" OR "Aquatic Therapy" OR "Therapeutic Irrigation" OR "Musculoskeletal Manipulations" OR "Applied Kinesiology" OR "Orthopedic Manipulation" OR "Orthopaedic Manipulation" OR "Osteopathic Manipulation" OR "Spinal Manipulation" OR "Continuous Passive Motion Therapy" OR "Soft Tissue Therapy" OR "Acupressure" OR "Massage" OR "Manual Lymphatic Drainage" OR "Myofascial Release Therapy" OR "mobilization" OR "mobilization" OR "mobilisation" OR "mobilizing" OR "mobilising" OR "mobilize" OR "mobilise" OR "mobilized" OR "mobilised" OR "mobility") OR SU("Physical Activity" OR "Exercise" OR "Exercise" OR "Exercises" OR "Exercising" OR "Exercis*" OR "physical activity" OR "physical activities" OR "Endurance Training" OR "Exergaming" OR "Gymnastics" OR "Gymnastic" OR "Interval Training" OR "Jogging" OR "Motor Activity" OR "Movement" OR "Muscle Stretching"OR "Physical Conditioning" OR "Resistance Training" OR "Running" OR "Stair Climbing" OR "Swimming" OR "Training" OR "Walking" OR "Kinesiotherapy" OR "Physical Exertion" OR "Physical Exertions" OR "Physical Effort" OR "Physical Efforts" OR "Sport" OR "Sports" OR "Sport" OR "Athletic Performance" OR "Baseball" OR "Basketball" OR "Bicycling" OR "Boxing" OR "Cardiorespiratory Fitness" OR "Cardiorespiratory Fitness" OR "Cricket Sport" OR "Diving" OR "Football" OR "Golf" OR "Gymnastics" OR "Hockey" OR "Jogging" OR "Marathon Running" OR "Martial Arts" OR "Mountaineering" OR "Nordic Walking" OR "Physical Endurance" OR "Physical Fitness" OR "Racquet Sports" OR "Return to Sport" OR "Rugby" OR "Running" OR "Skating" OR "Skiing" OR "Snow Sports" OR "Soccer" OR "Swimming" OR "Tai Ji" OR "Team Sports" OR "Tennis" OR "Track and Field" OR "Volleyball" OR "Walking" OR "Water Sports" OR "Weight Lifting" OR "Wrestling" OR "Youth Sports" OR "Qigong" OR "Dance Therapy" OR "Tai Ji" OR "Yoga" OR "Physiotherapy" OR "Physical Therapy" OR "physiotherapy" OR "physiotherapy*" OR "Dry Needling" OR "Electrotherapy" OR "Shock wave Therapy" OR "Balneotherapy" OR "Musculoskeletal Manipulation" OR "Dry Needling" OR "Electric Stimulation Therapy" OR "Electroacupuncture" OR "Pulsed Radiofrequency Treatment" OR "Spinal Cord Stimulation" OR "Transcutaneous Electric Nerve Stimulation" OR "Extracorporeal Shockwave Therapy" OR "Hydrotherapy" OR "Aquatic Therapy" OR "Therapeutic Irrigation" OR "Musculoskeletal Manipulations" OR "Applied Kinesiology" OR "Orthopedic Manipulation" OR "Orthopaedic Manipulation" OR "Osteopathic Manipulation" OR "Spinal Manipulation" OR "Continuous Passive Motion Therapy" OR "Soft Tissue Therapy" OR "Acupressure" OR "Massage" OR "Manual Lymphatic Drainage" OR "Myofascial Release Therapy" OR "mobilization" OR "mobilization" OR "mobilisation" OR "mobilizing" OR "mobilising" OR "mobilize" OR "mobilise" OR "mobilized" OR "mobilised" OR "mobility") OR MW("Physical Activity" OR "Exercise" OR "Exercise" OR "Exercises" OR "Exercising" OR "Exercis*" OR "physical activity" OR "physical activities" OR "Endurance Training" OR "Exergaming" OR "Gymnastics" OR "Gymnastic" OR "Interval Training" OR "Jogging" OR "Motor Activity" OR "Movement" OR "Muscle Stretching"OR "Physical Conditioning" OR "Resistance Training" OR "Running" OR "Stair Climbing" OR "Swimming" OR "Training" OR "Walking" OR "Kinesiotherapy" OR "Physical Exertion" OR "Physical Exertions" OR "Physical Effort" OR "Physical Efforts" OR "Sport" OR "Sports" OR "Sport" OR "Athletic Performance" OR "Baseball" OR "Basketball" OR "Bicycling" OR "Boxing" OR "Cardiorespiratory Fitness" OR "Cardiorespiratory Fitness" OR "Cricket Sport" OR "Diving" OR "Football" OR "Golf" OR "Gymnastics" OR "Hockey" OR "Jogging" OR "Marathon Running" OR "Martial Arts" OR "Mountaineering" OR "Nordic Walking" OR "Physical Endurance" OR "Physical Fitness" OR "Racquet Sports" OR "Return to Sport" OR "Rugby" OR "Running" OR "Skating" OR "Skiing" OR "Snow Sports" OR "Soccer" OR "Swimming" OR "Tai Ji" OR "Team Sports" OR "Tennis" OR "Track and Field" OR "Volleyball" OR "Walking" OR "Water Sports" OR "Weight Lifting" OR "Wrestling" OR "Youth Sports" OR "Qigong" OR "Dance Therapy" OR "Tai Ji" OR "Yoga" OR "Physiotherapy" OR "Physical Therapy" OR "physiotherapy" OR "physiotherapy*" OR "Dry Needling" OR "Electrotherapy" OR "Shock wave Therapy" OR "Balneotherapy" OR "Musculoskeletal Manipulation" OR "Dry Needling" OR "Electric Stimulation Therapy" OR "Electroacupuncture" OR "Pulsed Radiofrequency Treatment" OR "Spinal Cord Stimulation" OR "Transcutaneous Electric Nerve Stimulation" OR "Extracorporeal Shockwave Therapy" OR "Hydrotherapy" OR "Aquatic Therapy" OR "Therapeutic Irrigation" OR "Musculoskeletal Manipulations" OR "Applied Kinesiology" OR "Orthopedic Manipulation" OR "Orthopaedic Manipulation" OR "Osteopathic Manipulation" OR "Spinal Manipulation" OR "Continuous Passive Motion Therapy" OR "Soft Tissue Therapy" OR "Acupressure" OR "Massage" OR "Manual Lymphatic Drainage" OR "Myofascial Release Therapy" OR "mobilization" OR "mobilization" OR "mobilisation" OR "mobilizing" OR "mobilising" OR "mobilize" OR "mobilise" OR "mobilized" OR "mobilised" OR "mobility") OR AB("Physical Activity" OR "Exercise" OR "Exercise" OR "Exercises" OR "Exercising" OR "Exercis*" OR "physical activity" OR "physical activities" OR "Endurance Training" OR "Exergaming" OR "Gymnastics" OR "Gymnastic" OR "Interval Training" OR "Jogging" OR "Motor Activity" OR "Movement" OR "Muscle Stretching"OR "Physical Conditioning" OR "Resistance Training" OR "Running" OR "Stair Climbing" OR "Swimming" OR "Training" OR "Walking" OR "Kinesiotherapy" OR "Physical Exertion" OR "Physical Exertions" OR "Physical Effort" OR "Physical Efforts" OR "Sport" OR "Sports" OR "Sport" OR "Athletic Performance" OR "Baseball" OR "Basketball" OR "Bicycling" OR "Boxing" OR "Cardiorespiratory Fitness" OR "Cardiorespiratory Fitness" OR "Cricket Sport" OR "Diving" OR "Football" OR "Golf" OR "Gymnastics" OR "Hockey" OR "Jogging" OR "Marathon Running" OR "Martial Arts" OR "Mountaineering" OR "Nordic Walking" OR "Physical Endurance" OR "Physical Fitness" OR "Racquet Sports" OR "Return to Sport" OR "Rugby" OR "Running" OR "Skating" OR "Skiing" OR "Snow Sports" OR "Soccer" OR "Swimming" OR "Tai Ji" OR "Team Sports" OR "Tennis" OR "Track and Field" OR "Volleyball" OR "Walking" OR "Water Sports" OR "Weight Lifting" OR "Wrestling" OR "Youth Sports" OR "Qigong" OR "Dance Therapy" OR "Tai Ji" OR "Yoga" OR "Physiotherapy" OR "Physical Therapy" OR "physiotherapy" OR "physiotherapy*" OR "Dry Needling" OR "Electrotherapy" OR "Shock wave Therapy" OR "Balneotherapy" OR "Musculoskeletal Manipulation" OR "Dry Needling" OR "Electric Stimulation Therapy" OR "Electroacupuncture" OR "Pulsed Radiofrequency Treatment" OR "Spinal Cord Stimulation" OR "Transcutaneous Electric Nerve Stimulation" OR "Extracorporeal Shockwave Therapy" OR "Hydrotherapy" OR "Aquatic Therapy" OR "Therapeutic Irrigation" OR "Musculoskeletal Manipulations" OR "Applied Kinesiology" OR "Orthopedic Manipulation" OR "Orthopaedic Manipulation" OR "Osteopathic Manipulation" OR "Spinal Manipulation" OR "Continuous Passive Motion Therapy" OR "Soft Tissue Therapy" OR "Acupressure" OR "Massage" OR "Manual Lymphatic Drainage" OR "Myofascial Release Therapy" OR "mobilization" OR "mobilization" OR "mobilisation" OR "mobilizing" OR "mobilising" OR "mobilize" OR "mobilise" OR "mobilized" OR "mobilised" OR "mobility")) AND (TI("Ankle Fracture" OR "Femur Fracture" OR "Fibula Fracture" OR "Knee Fracture" OR "Tibia Fracture" OR "Ankle Fractures" OR "Femoral Fractures" OR "Hip Fractures" OR "Femoral Neck Fractures" OR "Proximal Femoral Fractures" OR "Hoffa Fracture" OR "Proximal Femoral Fractures" OR "Fibula Fractures" OR "Knee Fractures" OR "Hoffa Fracture" OR "Patella Fracture" OR "Tibial Plateau Fractures" OR "Tibial Fractures" OR "Tibial Plateau Fractures" OR "Ankle Fracture" OR "Femoral Fracture" OR "Hip Fracture" OR "Femoral Neck Fracture" OR "Proximal Femoral Fracture" OR "Hoffa Fractures" OR "Proximal Femoral Fracture" OR "Fibula Fracture" OR "Knee Fracture" OR "Hoffa Fractures" OR "Patella Fractures" OR "Tibial Plateau Fracture" OR "Tibial Fracture" OR "Tibial Plateau Fracture" OR (("Lower Limb" OR "lower limb" OR "lower limbs" OR "lower extremity" OR "lower extremities" OR "Ankle" OR "Foot" OR "Heel" OR "Hip" OR "Knee" OR "Leg" OR "Ankles" OR "Feet" OR "Heels" OR "Hips" OR "Knees" OR "Legs") AND ("Fracture" OR "fracture" OR "fractures" OR "fractur*")) OR "immobility" OR "immobility" OR "immobile" OR "immobil*" OR "temporary" OR "non weight bearing" OR "non weight bearing" OR "nonweight bearing" OR "non weight bear*" OR "nonweight bear*") OR SU("Ankle Fracture" OR "Femur Fracture" OR "Fibula Fracture" OR "Knee Fracture" OR "Tibia Fracture" OR "Ankle Fractures" OR "Femoral Fractures" OR "Hip Fractures" OR "Femoral Neck Fractures" OR "Proximal Femoral Fractures" OR "Hoffa Fracture" OR "Proximal Femoral Fractures" OR "Fibula Fractures" OR "Knee Fractures" OR "Hoffa Fracture" OR "Patella Fracture" OR "Tibial Plateau Fractures" OR "Tibial Fractures" OR "Tibial Plateau Fractures" OR "Ankle Fracture" OR "Femoral Fracture" OR "Hip Fracture" OR "Femoral Neck Fracture" OR "Proximal Femoral Fracture" OR "Hoffa Fractures" OR "Proximal Femoral Fracture" OR "Fibula Fracture" OR "Knee Fracture" OR "Hoffa Fractures" OR "Patella Fractures" OR "Tibial Plateau Fracture" OR "Tibial Fracture" OR "Tibial Plateau Fracture" OR (("Lower Limb" OR "lower limb" OR "lower limbs" OR "lower extremity" OR "lower extremities" OR "Ankle" OR "Foot" OR "Heel" OR "Hip" OR "Knee" OR "Leg" OR "Ankles" OR "Feet" OR "Heels" OR "Hips" OR "Knees" OR "Legs") AND ("Fracture" OR "fracture" OR "fractures" OR "fractur*")) OR "immobility" OR "immobility" OR "immobile" OR "immobil*" OR "temporary" OR "non weight bearing" OR "non weight bearing" OR "nonweight bearing" OR "non weight bear*" OR "nonweight bear*") OR MW("Ankle Fracture" OR "Femur Fracture" OR "Fibula Fracture" OR "Knee Fracture" OR "Tibia Fracture" OR "Ankle Fractures" OR "Femoral Fractures" OR "Hip Fractures" OR "Femoral Neck Fractures" OR "Proximal Femoral Fractures" OR "Hoffa Fracture" OR "Proximal Femoral Fractures" OR "Fibula Fractures" OR "Knee Fractures" OR "Hoffa Fracture" OR "Patella Fracture" OR "Tibial Plateau Fractures" OR "Tibial Fractures" OR "Tibial Plateau Fractures" OR "Ankle Fracture" OR "Femoral Fracture" OR "Hip Fracture" OR "Femoral Neck Fracture" OR "Proximal Femoral Fracture" OR "Hoffa Fractures" OR "Proximal Femoral Fracture" OR "Fibula Fracture" OR "Knee Fracture" OR "Hoffa Fractures" OR "Patella Fractures" OR "Tibial Plateau Fracture" OR "Tibial Fracture" OR "Tibial Plateau Fracture" OR (("Lower Limb" OR "lower limb" OR "lower limbs" OR "lower extremity" OR "lower extremities" OR "Ankle" OR "Foot" OR "Heel" OR "Hip" OR "Knee" OR "Leg" OR "Ankles" OR "Feet" OR "Heels" OR "Hips" OR "Knees" OR "Legs") AND ("Fracture" OR "fracture" OR "fractures" OR "fractur*")) OR "immobility" OR "immobility" OR "immobile" OR "immobil*" OR "temporary" OR "non weight bearing" OR "non weight bearing" OR "nonweight bearing" OR "non weight bear*" OR "nonweight bear*") OR AB("Ankle Fracture" OR "Femur Fracture" OR "Fibula Fracture" OR "Knee Fracture" OR "Tibia Fracture" OR "Ankle Fractures" OR "Femoral Fractures" OR "Hip Fractures" OR "Femoral Neck Fractures" OR "Proximal Femoral Fractures" OR "Hoffa Fracture" OR "Proximal Femoral Fractures" OR "Fibula Fractures" OR "Knee Fractures" OR "Hoffa Fracture" OR "Patella Fracture" OR "Tibial Plateau Fractures" OR "Tibial Fractures" OR "Tibial Plateau Fractures" OR "Ankle Fracture" OR "Femoral Fracture" OR "Hip Fracture" OR "Femoral Neck Fracture" OR "Proximal Femoral Fracture" OR "Hoffa Fractures" OR "Proximal Femoral Fracture" OR "Fibula Fracture" OR "Knee Fracture" OR "Hoffa Fractures" OR "Patella Fractures" OR "Tibial Plateau Fracture" OR "Tibial Fracture" OR "Tibial Plateau Fracture" OR (("Lower Limb" OR "lower limb" OR "lower limbs" OR "lower extremity" OR "lower extremities" OR "Ankle" OR "Foot" OR "Heel" OR "Hip" OR "Knee" OR "Leg" OR "Ankles" OR "Feet" OR "Heels" OR "Hips" OR "Knees" OR "Legs") AND ("Fracture" OR "fracture" OR "fractures" OR "fractur*")) OR "immobility" OR "immobility" OR "immobile" OR "immobil*" OR "temporary" OR "non weight bearing" OR "non weight bearing" OR "nonweight bearing" OR "non weight bear*" OR "nonweight bear*"))**) OR** (TI("Wheelchair" OR "Wheelchairs" OR "Wheelchair" OR "Wheelchair*" OR "Wheel chairs" OR "Wheel chair" OR "Wheel chair*" OR "Mobility Scooter" OR "Mobility Scooters") AND TI("Physical Activity" OR "Exercise" OR "Exercise" OR "Exercises" OR "Exercising" OR "Exercis*" OR "physical activity" OR "physical activities" OR "Endurance Training" OR "Exergaming" OR "Gymnastics" OR "Gymnastic" OR "Interval Training" OR "Jogging" OR "Motor Activity" OR "Movement" OR "Muscle Stretching"OR "Physical Conditioning" OR "Resistance Training" OR "Running" OR "Stair Climbing" OR "Swimming" OR "Training" OR "Walking" OR "Kinesiotherapy" OR "Physical Exertion" OR "Physical Exertions" OR "Physical Effort" OR "Physical Efforts" OR "Sport" OR "Sports" OR "Sport" OR "Athletic Performance" OR "Baseball" OR "Basketball" OR "Bicycling" OR "Boxing" OR "Cardiorespiratory Fitness" OR "Cardiorespiratory Fitness" OR "Cricket Sport" OR "Diving" OR "Football" OR "Golf" OR "Gymnastics" OR "Hockey" OR "Jogging" OR "Marathon Running" OR "Martial Arts" OR "Mountaineering" OR "Nordic Walking" OR "Physical Endurance" OR "Physical Fitness" OR "Racquet Sports" OR "Return to Sport" OR "Rugby" OR "Running" OR "Skating" OR "Skiing" OR "Snow Sports" OR "Soccer" OR "Swimming" OR "Tai Ji" OR "Team Sports" OR "Tennis" OR "Track and Field" OR "Volleyball" OR "Walking" OR "Water Sports" OR "Weight Lifting" OR "Wrestling" OR "Youth Sports" OR "Qigong" OR "Dance Therapy" OR "Tai Ji" OR "Yoga" OR "Physiotherapy" OR "Physical Therapy" OR "physiotherapy" OR "physiotherapy*" OR "Dry Needling" OR "Electrotherapy" OR "Shock wave Therapy" OR "Balneotherapy" OR "Musculoskeletal Manipulation" OR "Dry Needling" OR "Electric Stimulation Therapy" OR "Electroacupuncture" OR "Pulsed Radiofrequency Treatment" OR "Spinal Cord Stimulation" OR "Transcutaneous Electric Nerve Stimulation" OR "Extracorporeal Shockwave Therapy" OR "Hydrotherapy" OR "Aquatic Therapy" OR "Therapeutic Irrigation" OR "Musculoskeletal Manipulations" OR "Applied Kinesiology" OR "Orthopedic Manipulation" OR "Orthopaedic Manipulation" OR "Osteopathic Manipulation" OR "Spinal Manipulation" OR "Continuous Passive Motion Therapy" OR "Soft Tissue Therapy" OR "Acupressure" OR "Massage" OR "Manual Lymphatic Drainage" OR "Myofascial Release Therapy" OR "mobilization" OR "mobilization" OR "mobilisation" OR "mobilizing" OR "mobilising" OR "mobilize" OR "mobilise" OR "mobilized" OR "mobilised" OR "mobility") AND TI**("elderly" OR "elder" OR "elders" OR "geriatr*" OR "Home for the Aged" OR "Elderly care" OR "Senior Center" OR "Nonagenarians" OR "Nonagenarian" OR "Octogenarians" OR "Octogenarian" OR "Centenarians" OR "Centenarian" OR "septuagenarian" OR "septuagenarians" OR "geront*" OR "older person" OR "old person" OR "older patient" OR "old patient" OR "older persons" OR "old persons" OR "older patients" OR "old patients" OR "older women" OR "old women" OR "older men" OR "old men" OR "old adult" OR "older adult" OR "old adults" OR "older adults" OR "Older individual" OR "Older individuals" OR "old people" OR "older people" OR "Oldest Old" OR "older population" OR "aging population" OR "aging population" OR "old aged" OR "old age" OR (("older" NEAR/3 "person") OR ("old" NEAR/3 "person") OR ("older" NEAR/3 "patient") OR ("old" NEAR/3 "patient") OR ("older" NEAR/3 "persons") OR ("old" NEAR/3 "persons") OR ("older" NEAR/3 "patients") OR ("old" NEAR/3 "patients") OR ("older" NEAR/3 "women") OR ("old" NEAR/3 "women") OR ("older" NEAR/3 "men") OR ("old" NEAR/3 "men") OR ("old" NEAR/3 "adult") OR ("older" NEAR/3 "adult") OR ("old" NEAR/3 "adults") OR ("older" NEAR/3 "adults") OR ("Older" NEAR/3 "individual") OR ("Older" NEAR/3 "individuals") OR ("old" NEAR/3 "people") OR ("older" NEAR/3 "people") OR ("Oldest" NEAR/3 "Old") OR ("older" NEAR/3 "population") OR ("aging" NEAR/3 "population") OR ("aging" NEAR/3 "population") OR ("old" NEAR/3 "aged") OR ("old" NEAR/3 "age") OR (**"60" NEAR/3 "year" NEAR/3 "old") OR ("61" NEAR/3 "year" NEAR/3 "old") OR ("62" NEAR/3 "year" NEAR/3 "old") OR ("63" NEAR/3 "year" NEAR/3 "old") OR ("64" NEAR/3 "year" NEAR/3 "old") OR ("65" NEAR/3 "year" NEAR/3 "old") OR ("66" NEAR/3 "year" NEAR/3 "old") OR ("67" NEAR/3 "year" NEAR/3 "old") OR ("68" NEAR/3 "year" NEAR/3 "old") OR ("69" NEAR/3 "year" NEAR/3 "old") OR ("60" NEAR/3 "years" NEAR/3 "old") OR ("61" NEAR/3 "years" NEAR/3 "old") OR ("62" NEAR/3 "years" NEAR/3 "old") OR ("63" NEAR/3 "years" NEAR/3 "old") OR ("64" NEAR/3 "years" NEAR/3 "old") OR ("65" NEAR/3 "years" NEAR/3 "old") OR ("66" NEAR/3 "years" NEAR/3 "old") OR ("67" NEAR/3 "years" NEAR/3 "old") OR ("68" NEAR/3 "years" NEAR/3 "old") OR ("69" NEAR/3 "years" NEAR/3 "old") OR ("70" NEAR/3 "year" NEAR/3 "old") OR ("71" NEAR/3 "year" NEAR/3 "old") OR ("72" NEAR/3 "year" NEAR/3 "old") OR ("73" NEAR/3 "year" NEAR/3 "old") OR ("74" NEAR/3 "year" NEAR/3 "old") OR ("75" NEAR/3 "year" NEAR/3 "old") OR ("76" NEAR/3 "year" NEAR/3 "old") OR ("77" NEAR/3 "year" NEAR/3 "old") OR ("78" NEAR/3 "year" NEAR/3 "old") OR ("79" NEAR/3 "year" NEAR/3 "old") OR ("70" NEAR/3 "years" NEAR/3 "old") OR ("71" NEAR/3 "years" NEAR/3 "old") OR ("72" NEAR/3 "years" NEAR/3 "old") OR ("73" NEAR/3 "years" NEAR/3 "old") OR ("74" NEAR/3 "years" NEAR/3 "old") OR ("75" NEAR/3 "years" NEAR/3 "old") OR ("76" NEAR/3 "years" NEAR/3 "old") OR ("77" NEAR/3 "years" NEAR/3 "old") OR ("78" NEAR/3 "years" NEAR/3 "old") OR ("79" NEAR/3 "years" NEAR/3 "old") OR ("80" NEAR/3 "year" NEAR/3 "old") OR ("81" NEAR/3 "year" NEAR/3 "old") OR ("82" NEAR/3 "year" NEAR/3 "old") OR ("83" NEAR/3 "year" NEAR/3 "old") OR ("84" NEAR/3 "year" NEAR/3 "old") OR ("85" NEAR/3 "year" NEAR/3 "old") OR ("86" NEAR/3 "year" NEAR/3 "old") OR ("87" NEAR/3 "year" NEAR/3 "old") OR ("88" NEAR/3 "year" NEAR/3 "old") OR ("89" NEAR/3 "year" NEAR/3 "old") OR ("80" NEAR/3 "years" NEAR/3 "old") OR ("81" NEAR/3 "years" NEAR/3 "old") OR ("82" NEAR/3 "years" NEAR/3 "old") OR ("83" NEAR/3 "years" NEAR/3 "old") OR ("84" NEAR/3 "years" NEAR/3 "old") OR ("85" NEAR/3 "years" NEAR/3 "old") OR ("86" NEAR/3 "years" NEAR/3 "old") OR ("87" NEAR/3 "years" NEAR/3 "old") OR ("88" NEAR/3 "years" NEAR/3 "old") OR ("89" NEAR/3 "years" NEAR/3 "old") OR ("90" NEAR/3 "year" NEAR/3 "old") OR ("91" NEAR/3 "year" NEAR/3 "old") OR ("92" NEAR/3 "year" NEAR/3 "old") OR ("93" NEAR/3 "year" NEAR/3 "old") OR ("94" NEAR/3 "year" NEAR/3 "old") OR ("95" NEAR/3 "year" NEAR/3 "old") OR ("96" NEAR/3 "year" NEAR/3 "old") OR ("97" NEAR/3 "year" NEAR/3 "old") OR ("98" NEAR/3 "year" NEAR/3 "old") OR ("99" NEAR/3 "year" NEAR/3 "old") OR ("90" NEAR/3 "years" NEAR/3 "old") OR ("91" NEAR/3 "years" NEAR/3 "old") OR ("92" NEAR/3 "years" NEAR/3 "old") OR ("93" NEAR/3 "years" NEAR/3 "old") OR ("94" NEAR/3 "years" NEAR/3 "old") OR ("95" NEAR/3 "years" NEAR/3 "old") OR ("96" NEAR/3 "years" NEAR/3 "old") OR ("97" NEAR/3 "years" NEAR/3 "old") OR ("98" NEAR/3 "years" NEAR/3 "old") OR ("99" NEAR/3 "years" NEAR/3 "old") OR ("100" NEAR/3 "year" NEAR/3 "old") OR ("101" NEAR/3 "year" NEAR/3 "old") OR ("102" NEAR/3 "year" NEAR/3 "old") OR ("103" NEAR/3 "year" NEAR/3 "old") OR ("104" NEAR/3 "year" NEAR/3 "old") OR ("105" NEAR/3 "year" NEAR/3 "old") OR ("106" NEAR/3 "year" NEAR/3 "old") OR ("107" NEAR/3 "year" NEAR/3 "old") OR ("108" NEAR/3 "year" NEAR/3 "old") OR ("109" NEAR/3 "year" NEAR/3 "old") OR ("100" NEAR/3 "years" NEAR/3 "old") OR ("101" NEAR/3 "years" NEAR/3 "old") OR ("102" NEAR/3 "years" NEAR/3 "old") OR ("103" NEAR/3 "years" NEAR/3 "old") OR ("104" NEAR/3 "years" NEAR/3 "old") OR ("105" NEAR/3 "years" NEAR/3 "old") OR ("106" NEAR/3 "years" NEAR/3 "old") OR ("107" NEAR/3 "years" NEAR/3 "old") OR ("108" NEAR/3 "years" NEAR/3 "old") OR ("109" NEAR/3 "years" NEAR/3 "old"**)))) OR** ((TI("Physical Activity" OR "Exercise" OR "Exercise" OR "Exercises" OR "Exercising" OR "Exercis*" OR "physical activity" OR "physical activities" OR "Endurance Training" OR "Exergaming" OR "Gymnastics" OR "Gymnastic" OR "Interval Training" OR "Jogging" OR "Motor Activity" OR "Movement" OR "Muscle Stretching"OR "Physical Conditioning" OR "Resistance Training" OR "Running" OR "Stair Climbing" OR "Swimming" OR "Training" OR "Walking" OR "Kinesiotherapy" OR "Physical Exertion" OR "Physical Exertions" OR "Physical Effort" OR "Physical Efforts" OR "Sport" OR "Sports" OR "Sport" OR "Athletic Performance" OR "Baseball" OR "Basketball" OR "Bicycling" OR "Boxing" OR "Cardiorespiratory Fitness" OR "Cardiorespiratory Fitness" OR "Cricket Sport" OR "Diving" OR "Football" OR "Golf" OR "Gymnastics" OR "Hockey" OR "Jogging" OR "Marathon Running" OR "Martial Arts" OR "Mountaineering" OR "Nordic Walking" OR "Physical Endurance" OR "Physical Fitness" OR "Racquet Sports" OR "Return to Sport" OR "Rugby" OR "Running" OR "Skating" OR "Skiing" OR "Snow Sports" OR "Soccer" OR "Swimming" OR "Tai Ji" OR "Team Sports" OR "Tennis" OR "Track and Field" OR "Volleyball" OR "Walking" OR "Water Sports" OR "Weight Lifting" OR "Wrestling" OR "Youth Sports" OR "Qigong" OR "Dance Therapy" OR "Tai Ji" OR "Yoga" OR "Physiotherapy" OR "Physical Therapy" OR "physiotherapy" OR "physiotherapy*" OR "Dry Needling" OR "Electrotherapy" OR "Shock wave Therapy" OR "Balneotherapy" OR "Musculoskeletal Manipulation" OR "Dry Needling" OR "Electric Stimulation Therapy" OR "Electroacupuncture" OR "Pulsed Radiofrequency Treatment" OR "Spinal Cord Stimulation" OR "Transcutaneous Electric Nerve Stimulation" OR "Extracorporeal Shockwave Therapy" OR "Hydrotherapy" OR "Aquatic Therapy" OR "Therapeutic Irrigation" OR "Musculoskeletal Manipulations" OR "Applied Kinesiology" OR "Orthopedic Manipulation" OR "Orthopaedic Manipulation" OR "Osteopathic Manipulation" OR "Spinal Manipulation" OR "Continuous Passive Motion Therapy" OR "Soft Tissue Therapy" OR "Acupressure" OR "Massage" OR "Manual Lymphatic Drainage" OR "Myofascial Release Therapy" OR "mobilization" OR "mobilization" OR "mobilisation" OR "mobilizing" OR "mobilising" OR "mobilize" OR "mobilise" OR "mobilized" OR "mobilised" OR "mobility") OR SU("Physical Activity" OR "Exercise" OR "Exercise" OR "Exercises" OR "Exercising" OR "Exercis*" OR "physical activity" OR "physical activities" OR "Endurance Training" OR "Exergaming" OR "Gymnastics" OR "Gymnastic" OR "Interval Training" OR "Jogging" OR "Motor Activity" OR "Movement" OR "Muscle Stretching"OR "Physical Conditioning" OR "Resistance Training" OR "Running" OR "Stair Climbing" OR "Swimming" OR "Training" OR "Walking" OR "Kinesiotherapy" OR "Physical Exertion" OR "Physical Exertions" OR "Physical Effort" OR "Physical Efforts" OR "Sport" OR "Sports" OR "Sport" OR "Athletic Performance" OR "Baseball" OR "Basketball" OR "Bicycling" OR "Boxing" OR "Cardiorespiratory Fitness" OR "Cardiorespiratory Fitness" OR "Cricket Sport" OR "Diving" OR "Football" OR "Golf" OR "Gymnastics" OR "Hockey" OR "Jogging" OR "Marathon Running" OR "Martial Arts" OR "Mountaineering" OR "Nordic Walking" OR "Physical Endurance" OR "Physical Fitness" OR "Racquet Sports" OR "Return to Sport" OR "Rugby" OR "Running" OR "Skating" OR "Skiing" OR "Snow Sports" OR "Soccer" OR "Swimming" OR "Tai Ji" OR "Team Sports" OR "Tennis" OR "Track and Field" OR "Volleyball" OR "Walking" OR "Water Sports" OR "Weight Lifting" OR "Wrestling" OR "Youth Sports" OR "Qigong" OR "Dance Therapy" OR "Tai Ji" OR "Yoga" OR "Physiotherapy" OR "Physical Therapy" OR "physiotherapy" OR "physiotherapy*" OR "Dry Needling" OR "Electrotherapy" OR "Shock wave Therapy" OR "Balneotherapy" OR "Musculoskeletal Manipulation" OR "Dry Needling" OR "Electric Stimulation Therapy" OR "Electroacupuncture" OR "Pulsed Radiofrequency Treatment" OR "Spinal Cord Stimulation" OR "Transcutaneous Electric Nerve Stimulation" OR "Extracorporeal Shockwave Therapy" OR "Hydrotherapy" OR "Aquatic Therapy" OR "Therapeutic Irrigation" OR "Musculoskeletal Manipulations" OR "Applied Kinesiology" OR "Orthopedic Manipulation" OR "Orthopaedic Manipulation" OR "Osteopathic Manipulation" OR "Spinal Manipulation" OR "Continuous Passive Motion Therapy" OR "Soft Tissue Therapy" OR "Acupressure" OR "Massage" OR "Manual Lymphatic Drainage" OR "Myofascial Release Therapy" OR "mobilization" OR "mobilization" OR "mobilisation" OR "mobilizing" OR "mobilising" OR "mobilize" OR "mobilise" OR "mobilized" OR "mobilised" OR "mobility") OR MW("Physical Activity" OR "Exercise" OR "Exercise" OR "Exercises" OR "Exercising" OR "Exercis*" OR "physical activity" OR "physical activities" OR "Endurance Training" OR "Exergaming" OR "Gymnastics" OR "Gymnastic" OR "Interval Training" OR "Jogging" OR "Motor Activity" OR "Movement" OR "Muscle Stretching"OR "Physical Conditioning" OR "Resistance Training" OR "Running" OR "Stair Climbing" OR "Swimming" OR "Training" OR "Walking" OR "Kinesiotherapy" OR "Physical Exertion" OR "Physical Exertions" OR "Physical Effort" OR "Physical Efforts" OR "Sport" OR "Sports" OR "Sport" OR "Athletic Performance" OR "Baseball" OR "Basketball" OR "Bicycling" OR "Boxing" OR "Cardiorespiratory Fitness" OR "Cardiorespiratory Fitness" OR "Cricket Sport" OR "Diving" OR "Football" OR "Golf" OR "Gymnastics" OR "Hockey" OR "Jogging" OR "Marathon Running" OR "Martial Arts" OR "Mountaineering" OR "Nordic Walking" OR "Physical Endurance" OR "Physical Fitness" OR "Racquet Sports" OR "Return to Sport" OR "Rugby" OR "Running" OR "Skating" OR "Skiing" OR "Snow Sports" OR "Soccer" OR "Swimming" OR "Tai Ji" OR "Team Sports" OR "Tennis" OR "Track and Field" OR "Volleyball" OR "Walking" OR "Water Sports" OR "Weight Lifting" OR "Wrestling" OR "Youth Sports" OR "Qigong" OR "Dance Therapy" OR "Tai Ji" OR "Yoga" OR "Physiotherapy" OR "Physical Therapy" OR "physiotherapy" OR "physiotherapy*" OR "Dry Needling" OR "Electrotherapy" OR "Shock wave Therapy" OR "Balneotherapy" OR "Musculoskeletal Manipulation" OR "Dry Needling" OR "Electric Stimulation Therapy" OR "Electroacupuncture" OR "Pulsed Radiofrequency Treatment" OR "Spinal Cord Stimulation" OR "Transcutaneous Electric Nerve Stimulation" OR "Extracorporeal Shockwave Therapy" OR "Hydrotherapy" OR "Aquatic Therapy" OR "Therapeutic Irrigation" OR "Musculoskeletal Manipulations" OR "Applied Kinesiology" OR "Orthopedic Manipulation" OR "Orthopaedic Manipulation" OR "Osteopathic Manipulation" OR "Spinal Manipulation" OR "Continuous Passive Motion Therapy" OR "Soft Tissue Therapy" OR "Acupressure" OR "Massage" OR "Manual Lymphatic Drainage" OR "Myofascial Release Therapy" OR "mobilization" OR "mobilization" OR "mobilisation" OR "mobilizing" OR "mobilising" OR "mobilize" OR "mobilise" OR "mobilized" OR "mobilised" OR "mobility") OR AB("Physical Activity" OR "Exercise" OR "Exercise" OR "Exercises" OR "Exercising" OR "Exercis*" OR "physical activity" OR "physical activities" OR "Endurance Training" OR "Exergaming" OR "Gymnastics" OR "Gymnastic" OR "Interval Training" OR "Jogging" OR "Motor Activity" OR "Movement" OR "Muscle Stretching"OR "Physical Conditioning" OR "Resistance Training" OR "Running" OR "Stair Climbing" OR "Swimming" OR "Training" OR "Walking" OR "Kinesiotherapy" OR "Physical Exertion" OR "Physical Exertions" OR "Physical Effort" OR "Physical Efforts" OR "Sport" OR "Sports" OR "Sport" OR "Athletic Performance" OR "Baseball" OR "Basketball" OR "Bicycling" OR "Boxing" OR "Cardiorespiratory Fitness" OR "Cardiorespiratory Fitness" OR "Cricket Sport" OR "Diving" OR "Football" OR "Golf" OR "Gymnastics" OR "Hockey" OR "Jogging" OR "Marathon Running" OR "Martial Arts" OR "Mountaineering" OR "Nordic Walking" OR "Physical Endurance" OR "Physical Fitness" OR "Racquet Sports" OR "Return to Sport" OR "Rugby" OR "Running" OR "Skating" OR "Skiing" OR "Snow Sports" OR "Soccer" OR "Swimming" OR "Tai Ji" OR "Team Sports" OR "Tennis" OR "Track and Field" OR "Volleyball" OR "Walking" OR "Water Sports" OR "Weight Lifting" OR "Wrestling" OR "Youth Sports" OR "Qigong" OR "Dance Therapy" OR "Tai Ji" OR "Yoga" OR "Physiotherapy" OR "Physical Therapy" OR "physiotherapy" OR "physiotherapy*" OR "Dry Needling" OR "Electrotherapy" OR "Shock wave Therapy" OR "Balneotherapy" OR "Musculoskeletal Manipulation" OR "Dry Needling" OR "Electric Stimulation Therapy" OR "Electroacupuncture" OR "Pulsed Radiofrequency Treatment" OR "Spinal Cord Stimulation" OR "Transcutaneous Electric Nerve Stimulation" OR "Extracorporeal Shockwave Therapy" OR "Hydrotherapy" OR "Aquatic Therapy" OR "Therapeutic Irrigation" OR "Musculoskeletal Manipulations" OR "Applied Kinesiology" OR "Orthopedic Manipulation" OR "Orthopaedic Manipulation" OR "Osteopathic Manipulation" OR "Spinal Manipulation" OR "Continuous Passive Motion Therapy" OR "Soft Tissue Therapy" OR "Acupressure" OR "Massage" OR "Manual Lymphatic Drainage" OR "Myofascial Release Therapy" OR "mobilization" OR "mobilization" OR "mobilisation" OR "mobilizing" OR "mobilising" OR "mobilize" OR "mobilise" OR "mobilized" OR "mobilised" OR "mobility")) AND TI("Ankle Fracture" OR "Femur Fracture" OR "Fibula Fracture" OR "Knee Fracture" OR "Tibia Fracture" OR "Ankle Fractures" OR "Femoral Fractures" OR "Hip Fractures" OR "Femoral Neck Fractures" OR "Proximal Femoral Fractures" OR "Hoffa Fracture" OR "Proximal Femoral Fractures" OR "Fibula Fractures" OR "Knee Fractures" OR "Hoffa Fracture" OR "Patella Fracture" OR "Tibial Plateau Fractures" OR "Tibial Fractures" OR "Tibial Plateau Fractures" OR "Ankle Fracture" OR "Femoral Fracture" OR "Hip Fracture" OR "Femoral Neck Fracture" OR "Proximal Femoral Fracture" OR "Hoffa Fractures" OR "Proximal Femoral Fracture" OR "Fibula Fracture" OR "Knee Fracture" OR "Hoffa Fractures" OR "Patella Fractures" OR "Tibial Plateau Fracture" OR "Tibial Fracture" OR "Tibial Plateau Fracture" OR (("Lower Limb" OR "lower limb" OR "lower limbs" OR "lower extremity" OR "lower extremities" OR "Ankle" OR "Foot" OR "Heel" OR "Hip" OR "Knee" OR "Leg" OR "Ankles" OR "Feet" OR "Heels" OR "Hips" OR "Knees" OR "Legs") AND ("Fracture" OR "fracture" OR "fractures" OR "fractur*")) OR **"acutely ill"**) AND (TI("immobility" OR "immobility" OR "immobile" OR "immobil*" OR "temporary" OR "non weight bearing" OR "non weight bearing" OR "nonweight bearing" OR "non weight bear*" OR "nonweight bear*" OR "physically dependent" OR "physically disabled" OR **"physically dependent" OR "physically disabled" OR "disuse" OR "bed rest" OR "bed rest" OR "bedrest" OR "leg immobilization" OR "leg immobilisation" OR "Weightlessness" OR "deconditioning" OR "deconditioning" OR "permissive weight-bearing"**) OR SU("immobility" OR "immobility" OR "immobile" OR "immobil*" OR "temporary" OR "non weight bearing" OR "non weight bearing" OR "nonweight bearing" OR "non weight bear*" OR "nonweight bear*" OR "physically dependent" OR "physically disabled" OR **"physically dependent" OR "physically disabled" OR "disuse" OR "bed rest" OR "bed rest" OR "bedrest" OR "leg immobilization" OR "leg immobilisation" OR "Weightlessness" OR "deconditioning" OR "deconditioning" OR "permissive weight-bearing"**) OR MW("immobility" OR "immobility" OR "immobile" OR "immobil*" OR "temporary" OR "non weight bearing" OR "non weight bearing" OR "nonweight bearing" OR "non weight bear*" OR "nonweight bear*" OR "physically dependent" OR "physically disabled" OR **"physically dependent" OR "physically disabled" OR "disuse" OR "bed rest" OR "bed rest" OR "bedrest" OR "leg immobilization" OR "leg immobilisation" OR "Weightlessness" OR "deconditioning" OR "deconditioning" OR "permissive weight-bearing"**) OR AB("immobility" OR "immobility" OR "immobile" OR "immobil*" OR "temporary" OR "non weight bearing" OR "non weight bearing" OR "nonweight bearing" OR "non weight bear*" OR "nonweight bear*" OR "physically dependent" OR "physically disabled" OR **"physically dependent" OR "physically disabled" OR "disuse" OR "bed rest" OR "bed rest" OR "bedrest" OR "leg immobilization" OR "leg immobilisation" OR "Weightlessness" OR "deconditioning" OR "deconditioning" OR "permissive weight-bearing"**))**) OR** (TI("Physical Activity" OR "Exercise" OR "Exercise" OR "Exercises" OR "Exercising" OR "Exercis*" OR "physical activity" OR "physical activities" OR "Endurance Training" OR "Exergaming" OR "Gymnastics" OR "Gymnastic" OR "Interval Training" OR "Jogging" OR "Motor Activity" OR "Movement" OR "Muscle Stretching"OR "Physical Conditioning" OR "Resistance Training" OR "Running" OR "Stair Climbing" OR "Swimming" OR "Training" OR "Walking" OR "Kinesiotherapy" OR "Physical Exertion" OR "Physical Exertions" OR "Physical Effort" OR "Physical Efforts" OR "Sport" OR "Sports" OR "Sport" OR "Athletic Performance" OR "Baseball" OR "Basketball" OR "Bicycling" OR "Boxing" OR "Cardiorespiratory Fitness" OR "Cardiorespiratory Fitness" OR "Cricket Sport" OR "Diving" OR "Football" OR "Golf" OR "Gymnastics" OR "Hockey" OR "Jogging" OR "Marathon Running" OR "Martial Arts" OR "Mountaineering" OR "Nordic Walking" OR "Physical Endurance" OR "Physical Fitness" OR "Racquet Sports" OR "Return to Sport" OR "Rugby" OR "Running" OR "Skating" OR "Skiing" OR "Snow Sports" OR "Soccer" OR "Swimming" OR "Tai Ji" OR "Team Sports" OR "Tennis" OR "Track and Field" OR "Volleyball" OR "Walking" OR "Water Sports" OR "Weight Lifting" OR "Wrestling" OR "Youth Sports" OR "Qigong" OR "Dance Therapy" OR "Tai Ji" OR "Yoga" OR "Physiotherapy" OR "Physical Therapy" OR "physiotherapy" OR "physiotherapy*" OR "Dry Needling" OR "Electrotherapy" OR "Shock wave Therapy" OR "Balneotherapy" OR "Musculoskeletal Manipulation" OR "Dry Needling" OR "Electric Stimulation Therapy" OR "Electroacupuncture" OR "Pulsed Radiofrequency Treatment" OR "Spinal Cord Stimulation" OR "Transcutaneous Electric Nerve Stimulation" OR "Extracorporeal Shockwave Therapy" OR "Hydrotherapy" OR "Aquatic Therapy" OR "Therapeutic Irrigation" OR "Musculoskeletal Manipulations" OR "Applied Kinesiology" OR "Orthopedic Manipulation" OR "Orthopaedic Manipulation" OR "Osteopathic Manipulation" OR "Spinal Manipulation" OR "Continuous Passive Motion Therapy" OR "Soft Tissue Therapy" OR "Acupressure" OR "Massage" OR "Manual Lymphatic Drainage" OR "Myofascial Release Therapy" OR "mobilization" OR "mobilization" OR "mobilisation" OR "mobilizing" OR "mobilising" OR "mobilize" OR "mobilise" OR "mobilized" OR "mobilised" OR "mobility") AND (TI(**"disuse"**) OR SU(**"disuse"**) OR MW(**"disuse"**) OR AB(**"disuse"**)) AND TI**("elderly" OR "elder" OR "elders" OR "geriatr*" OR "Home for the Aged" OR "Elderly care" OR "Senior Center" OR "Nonagenarians" OR "Nonagenarian" OR "Octogenarians" OR "Octogenarian" OR "Centenarians" OR "Centenarian" OR "septuagenarian" OR "septuagenarians" OR "geront*" OR "older person" OR "old person" OR "older patient" OR "old patient" OR "older persons" OR "old persons" OR "older patients" OR "old patients" OR "older women" OR "old women" OR "older men" OR "old men" OR "old adult" OR "older adult" OR "old adults" OR "older adults" OR "Older individual" OR "Older individuals" OR "old people" OR "older people" OR "Oldest Old" OR "older population" OR "aging population" OR "aging population" OR "old aged" OR "old age" OR (("older" NEAR/3 "person") OR ("old" NEAR/3 "person") OR ("older" NEAR/3 "patient") OR ("old" NEAR/3 "patient") OR ("older" NEAR/3 "persons") OR ("old" NEAR/3 "persons") OR ("older" NEAR/3 "patients") OR ("old" NEAR/3 "patients") OR ("older" NEAR/3 "women") OR ("old" NEAR/3 "women") OR ("older" NEAR/3 "men") OR ("old" NEAR/3 "men") OR ("old" NEAR/3 "adult") OR ("older" NEAR/3 "adult") OR ("old" NEAR/3 "adults") OR ("older" NEAR/3 "adults") OR ("Older" NEAR/3 "individual") OR ("Older" NEAR/3 "individuals") OR ("old" NEAR/3 "people") OR ("older" NEAR/3 "people") OR ("Oldest" NEAR/3 "Old") OR ("older" NEAR/3 "population") OR ("aging" NEAR/3 "population") OR ("aging" NEAR/3 "population") OR ("old" NEAR/3 "aged") OR ("old" NEAR/3 "age") OR (**"60" NEAR/3 "year" NEAR/3 "old") OR ("61" NEAR/3 "year" NEAR/3 "old") OR ("62" NEAR/3 "year" NEAR/3 "old") OR ("63" NEAR/3 "year" NEAR/3 "old") OR ("64" NEAR/3 "year" NEAR/3 "old") OR ("65" NEAR/3 "year" NEAR/3 "old") OR ("66" NEAR/3 "year" NEAR/3 "old") OR ("67" NEAR/3 "year" NEAR/3 "old") OR ("68" NEAR/3 "year" NEAR/3 "old") OR ("69" NEAR/3 "year" NEAR/3 "old") OR ("60" NEAR/3 "years" NEAR/3 "old") OR ("61" NEAR/3 "years" NEAR/3 "old") OR ("62" NEAR/3 "years" NEAR/3 "old") OR ("63" NEAR/3 "years" NEAR/3 "old") OR ("64" NEAR/3 "years" NEAR/3 "old") OR ("65" NEAR/3 "years" NEAR/3 "old") OR ("66" NEAR/3 "years" NEAR/3 "old") OR ("67" NEAR/3 "years" NEAR/3 "old") OR ("68" NEAR/3 "years" NEAR/3 "old") OR ("69" NEAR/3 "years" NEAR/3 "old") OR ("70" NEAR/3 "year" NEAR/3 "old") OR ("71" NEAR/3 "year" NEAR/3 "old") OR ("72" NEAR/3 "year" NEAR/3 "old") OR ("73" NEAR/3 "year" NEAR/3 "old") OR ("74" NEAR/3 "year" NEAR/3 "old") OR ("75" NEAR/3 "year" NEAR/3 "old") OR ("76" NEAR/3 "year" NEAR/3 "old") OR ("77" NEAR/3 "year" NEAR/3 "old") OR ("78" NEAR/3 "year" NEAR/3 "old") OR ("79" NEAR/3 "year" NEAR/3 "old") OR ("70" NEAR/3 "years" NEAR/3 "old") OR ("71" NEAR/3 "years" NEAR/3 "old") OR ("72" NEAR/3 "years" NEAR/3 "old") OR ("73" NEAR/3 "years" NEAR/3 "old") OR ("74" NEAR/3 "years" NEAR/3 "old") OR ("75" NEAR/3 "years" NEAR/3 "old") OR ("76" NEAR/3 "years" NEAR/3 "old") OR ("77" NEAR/3 "years" NEAR/3 "old") OR ("78" NEAR/3 "years" NEAR/3 "old") OR ("79" NEAR/3 "years" NEAR/3 "old") OR ("80" NEAR/3 "year" NEAR/3 "old") OR ("81" NEAR/3 "year" NEAR/3 "old") OR ("82" NEAR/3 "year" NEAR/3 "old") OR ("83" NEAR/3 "year" NEAR/3 "old") OR ("84" NEAR/3 "year" NEAR/3 "old") OR ("85" NEAR/3 "year" NEAR/3 "old") OR ("86" NEAR/3 "year" NEAR/3 "old") OR ("87" NEAR/3 "year" NEAR/3 "old") OR ("88" NEAR/3 "year" NEAR/3 "old") OR ("89" NEAR/3 "year" NEAR/3 "old") OR ("80" NEAR/3 "years" NEAR/3 "old") OR ("81" NEAR/3 "years" NEAR/3 "old") OR ("82" NEAR/3 "years" NEAR/3 "old") OR ("83" NEAR/3 "years" NEAR/3 "old") OR ("84" NEAR/3 "years" NEAR/3 "old") OR ("85" NEAR/3 "years" NEAR/3 "old") OR ("86" NEAR/3 "years" NEAR/3 "old") OR ("87" NEAR/3 "years" NEAR/3 "old") OR ("88" NEAR/3 "years" NEAR/3 "old") OR ("89" NEAR/3 "years" NEAR/3 "old") OR ("90" NEAR/3 "year" NEAR/3 "old") OR ("91" NEAR/3 "year" NEAR/3 "old") OR ("92" NEAR/3 "year" NEAR/3 "old") OR ("93" NEAR/3 "year" NEAR/3 "old") OR ("94" NEAR/3 "year" NEAR/3 "old") OR ("95" NEAR/3 "year" NEAR/3 "old") OR ("96" NEAR/3 "year" NEAR/3 "old") OR ("97" NEAR/3 "year" NEAR/3 "old") OR ("98" NEAR/3 "year" NEAR/3 "old") OR ("99" NEAR/3 "year" NEAR/3 "old") OR ("90" NEAR/3 "years" NEAR/3 "old") OR ("91" NEAR/3 "years" NEAR/3 "old") OR ("92" NEAR/3 "years" NEAR/3 "old") OR ("93" NEAR/3 "years" NEAR/3 "old") OR ("94" NEAR/3 "years" NEAR/3 "old") OR ("95" NEAR/3 "years" NEAR/3 "old") OR ("96" NEAR/3 "years" NEAR/3 "old") OR ("97" NEAR/3 "years" NEAR/3 "old") OR ("98" NEAR/3 "years" NEAR/3 "old") OR ("99" NEAR/3 "years" NEAR/3 "old") OR ("100" NEAR/3 "year" NEAR/3 "old") OR ("101" NEAR/3 "year" NEAR/3 "old") OR ("102" NEAR/3 "year" NEAR/3 "old") OR ("103" NEAR/3 "year" NEAR/3 "old") OR ("104" NEAR/3 "year" NEAR/3 "old") OR ("105" NEAR/3 "year" NEAR/3 "old") OR ("106" NEAR/3 "year" NEAR/3 "old") OR ("107" NEAR/3 "year" NEAR/3 "old") OR ("108" NEAR/3 "year" NEAR/3 "old") OR ("109" NEAR/3 "year" NEAR/3 "old") OR ("100" NEAR/3 "years" NEAR/3 "old") OR ("101" NEAR/3 "years" NEAR/3 "old") OR ("102" NEAR/3 "years" NEAR/3 "old") OR ("103" NEAR/3 "years" NEAR/3 "old") OR ("104" NEAR/3 "years" NEAR/3 "old") OR ("105" NEAR/3 "years" NEAR/3 "old") OR ("106" NEAR/3 "years" NEAR/3 "old") OR ("107" NEAR/3 "years" NEAR/3 "old") OR ("108" NEAR/3 "years" NEAR/3 "old") OR ("109" NEAR/3 "years" NEAR/3 "old"**))))) NOT TI("veterinary" OR "rabbit" OR "rabbits" OR "animal" OR "animals" OR "mouse" OR "mice" OR "rodent" OR "rodents" OR "rat" OR "rats" OR "pig" OR "pigs" OR "porcine" OR "horse" OR "horses" OR "equine" OR "cow" OR "cows" OR "bovine" OR "goat" OR "goats" OR "sheep" OR "ovine" OR "canine" OR "dog" OR "dogs" OR "feline" OR "cat" OR "cats")** NOT TI(("Case Report" OR "review") NOT ("Clinical Trial" OR "trial" OR "RCT" OR "systematic"))
